# Asymmetric C–H functionalization of cyclopropanes using an isoleucine-NH_2_ bidentate directing group†
†Electronic supplementary information (ESI) available. CCDC 1053232. For ESI and crystallographic data in CIF or other electronic format see DOI: 10.1039/c5sc01137j


**DOI:** 10.1039/c5sc01137j

**Published:** 2015-04-16

**Authors:** Jinhee Kim, Mikyung Sim, Namhoon Kim, Sungwoo Hong

**Affiliations:** a Department of Chemistry , Korea Advanced Institute of Science and Technology , Daejeon , 305-701 , Korea; b Center for Catalytic Hydrocarbon Functionalization , Institute for Basic Science (IBS) , Daejeon , 305-701 , Korea . Email: hongorg@kaist.ac.kr

## Abstract

The systematic investigation of substrate-bound α-amino acid auxiliaries has resulted in catalytic asymmetric C–H functionalization of cyclopropanes enabled by amino acid amides as chiral bidentate directing groups. The use of an Ile-NH_2_ auxiliary embedded in the substrate provided excellent levels of asymmetric induction (diastereomeric ratio of up to 72 : 1) in the Pd(ii)-catalyzed β-methylene C(sp^3^)–H bond activation of cyclopropanes and cross-coupling with aryl iodides.

## Abstract 2

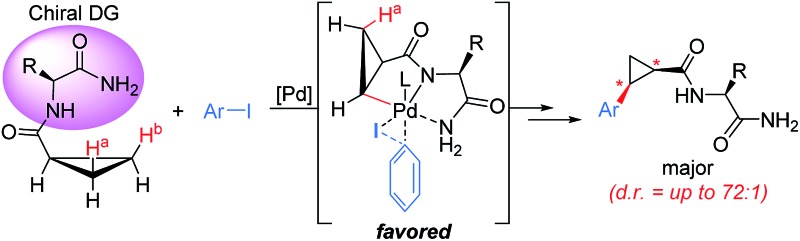
The use of an Ile-NH_2_ auxiliary can provide excellent levels of asymmetric induction in the Pd(ii)-catalyzed C(sp^3^)–H functionalization of cyclopropanes.

## Introduction

Transition-metal-catalyzed direct and regioselective C–H bond activation/functionalization is a highly efficient and straightforward tool that is useful in the field of organic synthesis and total synthesis.^1^ To achieve a highly selective C–H activation, the directing group embedded in the substrate must coordinate to a transition-metal center in a configuration that allows for the cleavage of a specific C–H bond, generally *via* the efficient formation of 5- or 6-membered metallacyclic intermediates.^2^ Since the novel discovery by Daugulis' group, powerful bidentate directing groups, such as 8-aminoquinoline and picolinamide auxiliaries, have been widely used in the activation of both C(sp^2^)–H and C(sp^3^)–H bonds.^3^ Bidentate directing groups have received considerable attention by granting favorable properties to the metalated complex, thus enabling new types of catalytic transformations that are known to be difficult with conventional monodentate systems.^4^ For improving the convenience and efficiency, the development of new types of bidentate directing groups has been the subject of intensive research in many groups.^5^ Recently, Chatani's group accomplished *ortho*-C(sp^2^)–H activation using α-amino ester moieties as bidentate directing groups.^6^ Moreover, Yu and coworkers demonstrated that the amino acid moiety of peptides can promote the functionalization of C(sp^3^)–H bonds in a number of peptides.^7^

**Scheme 1:**
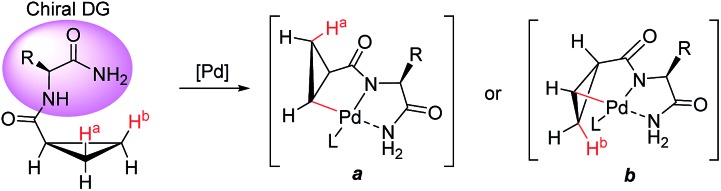
Amino acid auxiliary-controlled asymmetric C–H functionalization of cyclopropanes.

Elegant examples of the diastereoselective functionalization of unactivated C(sp^3^)–H in chiral substrates were revealed by the Corey,3*b* Daugulis3*q* and Chen groups.3d,3m Moreover, remarkable achievements in catalytic asymmetric C–H activation have been demonstrated using a chiral auxiliary^8^ and a chiral ligand.^9^ We hypothesized that an appropriate chiral bidentate directing group embedded in the substrate could induce high levels of stereocontrol during C–H functionalization *via* a steric repulsion model. As an outgrowth of these studies, we selected α-amino acid moieties as chiral bidentate directing groups in the context of the diastereotopic β-methylene C(sp^3^)–H bond functionalization/arylation of cyclopropanes, enabling the stereoselective installation of an aryl group and the construction of new stereogenic centers (Scheme 1). Cyclopropanes are a privileged class of structures found in many biologically active molecules.^10^ In this regard, the transition-metal-catalyzed asymmetric C–H arylation of cyclopropanes has been the focus of great research interest.^9^,^11^ Moreover, functionalized cyclopropanes bearing amino acid moieties are widely used as a conformationally restricting linker in medicinal chemistry.10d,e Herein, we report the first example of chiral bidentate auxiliary-controlled diastereoselective C(sp^3^)–H bond activation/cross-coupling of cyclopropanes. From a conceptual viewpoint, the ability of a substrate-bound α-amino acid auxiliary to promote diastereoselective C–H functionalization is intriguing because the amino acid moiety derived from readily available chiral pools not only plays the role of a bidentate directing group, but also of a chiral auxiliary to provide efficient stereocontrol during C(sp^3^)–H bond functionalization. As a consequence, high levels of asymmetric induction could be expected in the C(sp^3^)–H functionalization of cyclopropanes controlled by a chiral bidentate directing group.

## Results and discussion

**Fig. 1:**
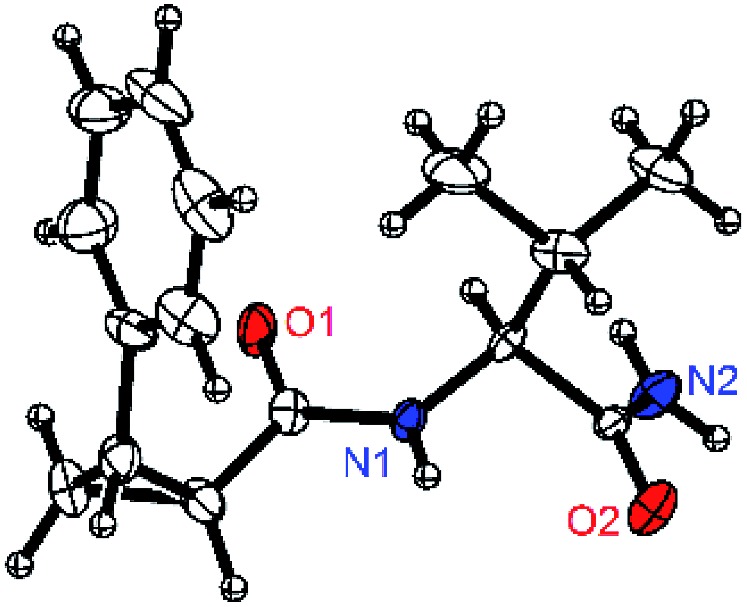
X-ray crystal structure for compound **2a** (Val–NH_2_ derivative).

To test these hypotheses, we began our studies by investigating the direct β-methylene C(sp^3^)–H functionalization of a cyclopropane framework bearing a valine moiety as a potential directing group. Initially, we performed a diastereoselective C–H arylation with iodobenzene using Pd(OAc)_2_ (10 mol%) and K_2_CO_3_ as a base in *t*-amyl-OH at 100 °C for 24 h (Table 1). Our early investigations were discouraging because of the lack of reactivity of the l-valine directing group (**1a**) (entry 1). Subsequently, several other auxiliaries, such as ester (**1b**), tetrazole (**1c**), and amino acid amide groups (**1d**, **1e**, **1f**), were investigated for feasibility as directing groups for C(sp^3^)–H arylation. The usage of ester or tetrazole moieties as directing groups was unsuccessful for C–H activation/C–C bond formation (entries 2 and 3). Intriguingly, the C(sp^3^)–H bond activation of a substrate containing a Val–NHMe auxiliary (**1d**) occurred effectively to afford the desired *cis*-substituted phenylcyclopropane product with a 26% yield and high diastereoselectivity (entry 4, d.r = 8.1 : 1). This preliminary work prompted us to scrutinize additional types of amino acid amides as stereogenic directing groups. Further investigations revealed that having the Val–NH_2_ group (**1f**) was crucial for obtaining a higher reactivity and diastereoselectivity (entry 6, 60%, d.r = 8.5 : 1). The Val–NMe_2_ group (**1e**) attached to the substrate led to a complete loss of reactivity (entry 5), indicating the favorable property of an NH_2_ motif on coordination in the active complex. We therefore decided to employ Val–NH_2_ (**1f**) as a directing group for further optimization; representative catalytic systems for the cross-coupling are listed in Table 1 (entries 6–11).^12^ In general, an approximate 4.3 : 1 mixture of mono- and diarylated products were obtained with substrate **1f**, containing Val–NH_2_, at 100 °C. Attempts to improve the conversion at a higher temperature (120 °C, entry 7) resulted in an increased proportion of the diarylated product (81%, *mono*/*di* = 2.5 : 1, d.r = 6.3 : 1) with mixtures of polyarylated by-products^13^ (∼10%). Several solvents were also evaluated and the use of *t*-amyl-OH as the solvent was necessary to achieve a higher conversion. The use of AgOAc (entry 9), which was employed previously in arylation reactions,10d,e provided a lower product yield (20%) along with the recovery of the remaining starting material (58%). Alternative arylating coupling partners, such as diphenyliodonium salt3*o* failed to improve the reaction efficiency (entry 10). With the use of 1.5 equiv. of iodobenzene, the reaction reached full conversion to provide the mono- and diarylated products in yields of 71 and 16%, respectively, with only a negligible amount of the undesired by-products. Thus, the best results were obtained with the substrate (1 equiv.) with iodobenzene (1.5 equiv.), Pd(OAc)_2_ (10 mol%), and K_2_CO_3_ (2 equiv.) in *t*-amyl-OH (0.5 M) at 100 °C to give the monoarylation product (71%) with high diastereoselectivity (d.r = 8.6 : 1) (entry 11). The structure of the major diastereomer **2a** (Val–NH_2_ derivative) was unambiguously verified by H NMR and X-ray crystallographic analyses (Fig. 1).

**Table 1:** Screening of potential bidentate chiral auxiliaries for C–H arylation with iodobenzene^*a*^

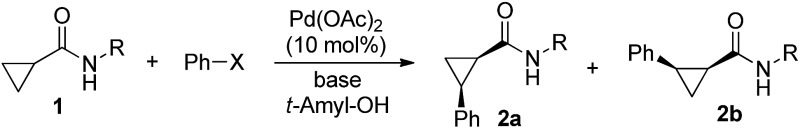					
Entry	R	PhX (equiv.)	Base (equiv.)	Temp. (°C)	Yield^*b*^ (d.r)^*c*^
1	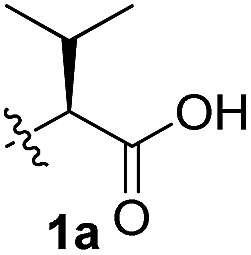	PhI (3)	K_2_CO_3_ (2.5)	100	Trace
2	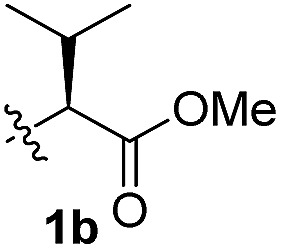	PhI (3)	K_2_CO_3_ (2.5)	100	NR
3	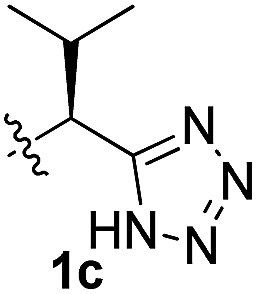	PhI (3)	K_2_CO_3_ (2.5)	100	NR
4	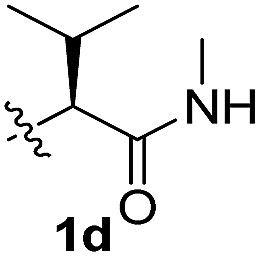	PhI (3)	K_2_CO_3_ (2.5)	100	26% (8.1 : 1)
5	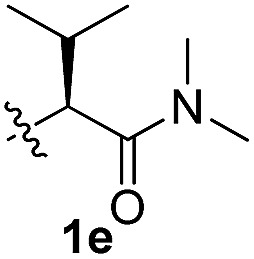	PhI (3)	K_2_CO_3_ (2.5)	100	NR
6	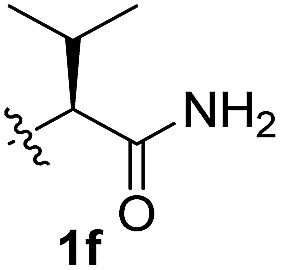	PhI (3)	K_2_CO_3_ (2.5)	100	60% (8.5 : 1)
7		PhI (3)	K_2_CO_3_ (2.5)	120	58% (6.3 : 1)
8		PhI (3)	K_2_CO_3_ (2.5)	80	12%
9		PhI (4)	AgOAc (2.5)	100	20%
10		Ph_2_IOTf (2)	K_2_CO_3_ (3)	100	33%
11^*d*^		PhI (1.5)	K_2_CO_3_ (2)	100	71% (8.6 : 1)

^*a*^Substrate (1.0 equiv.), Pd(OAc)_2_ (10 mol%), K_2_CO_3_ (2.5 equiv.), and ArX in *t*-amyl-OH (0.5 M).

^*b*^Isolated yields of monoarylation products.

^*c*^The d.r was determined by HPLC analysis.

^*d*^Mono- and diarylation products (4.5 : 1) were observed by NMR analysis.

**Scheme 2:**
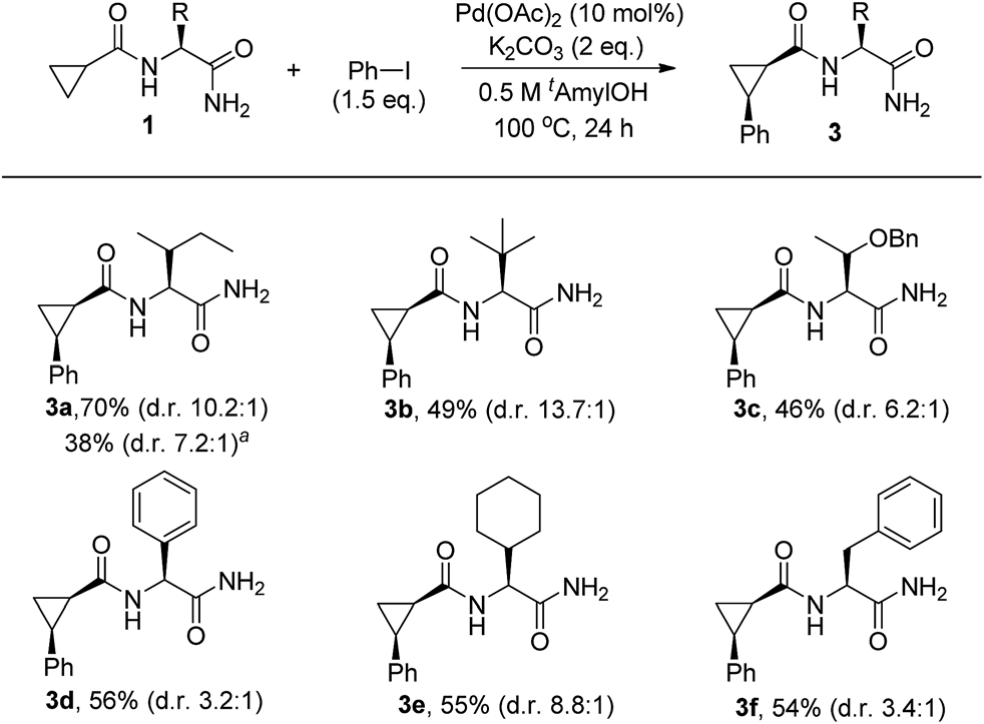
Influence of the side chain on C–H arylation. Substrate (1.0 equiv.), Pd(OAc)_2_ (10 mol%), K_2_CO_3_ (2.0 equiv.), and PhI (1.5 equiv.) in *t*-amyl-OH (0.5 M) at 100 °C for 24 h: isolated yields of monoarylation products after purification by silica gel chromatography. The d.r was determined by HPLC analysis. In general, mixtures of mono- and diarylation products (4.0–4.5 : 1) were observed by NMR analysis. ^*a*^After 1 h: NMR yield of monoarylation products (see Table S4 in the ESI† for more details).

Having identified that the N-nonsubstituted –CONH_2_ moiety was crucial for achieving both high reactivity and diastereoselectivity, we further explored the effect of steric bulk at the α-position of amino acid amides on the selectivity. For this purpose, substrates bearing a range of R substituents were examined and chiral auxiliaries featuring sterically bulky side chains appear to be essential for achieving high levels of stereoselectivity (Scheme 2). Importantly, the replacement of an isopropyl group (d.r = 8.6 : 1) at the α-position of the amino acid amide with sterically demanding isobutyl and *t*-butyl groups produced the corresponding products **3a** and **3b** with higher diastereoselectivity (d.r = 10.2 : 1 for **3a**, d.r = 13.7 : 1 for **3b**). Further studies revealed that the isobutyl and *t*-butyl groups present on the substrates gave superior results to those with benzyloxy ethyl (**3c**), phenyl (**3d**), cyclohexyl (**3e**), or benzyl (**3f**) groups. Of the various chiral auxiliaries that were tested, the Ile-NH_2_ moiety (**3a**) was selected as a bidentate directing group after considering both yield and diastereoselectivity. In order to gain insight into the influence of the diarylation process on the diastereoselectivity, a reaction profile was performed by monitoring the conversion to **3a** under standard conditions (see Table S4 in the ESI† for more details). An increase of d.r from 7.2 : 1 (after 1 h) to 10.2 : 1 (after 24 h) was observed, which suggests that the favorable second arylation of the minor stereoisomer may contribute, in part, to the overall diastereoselectivity observed.

The directing group is removable and the hydrolysis of the amino acid amide group of **3a** occurred smoothly under acidic conditions to afford arylated cyclopropanecarboxylic acid (81%) with the conservation of the stereogenic centers (eqn (1)).^14^,^15^
1
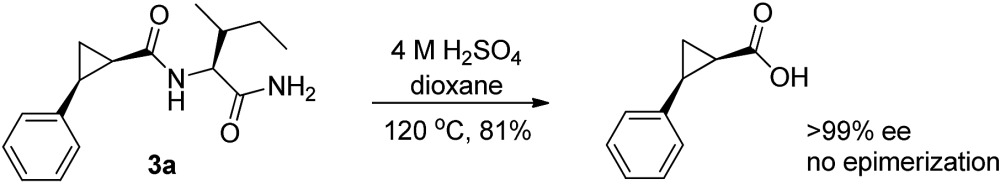



**Scheme 3:**
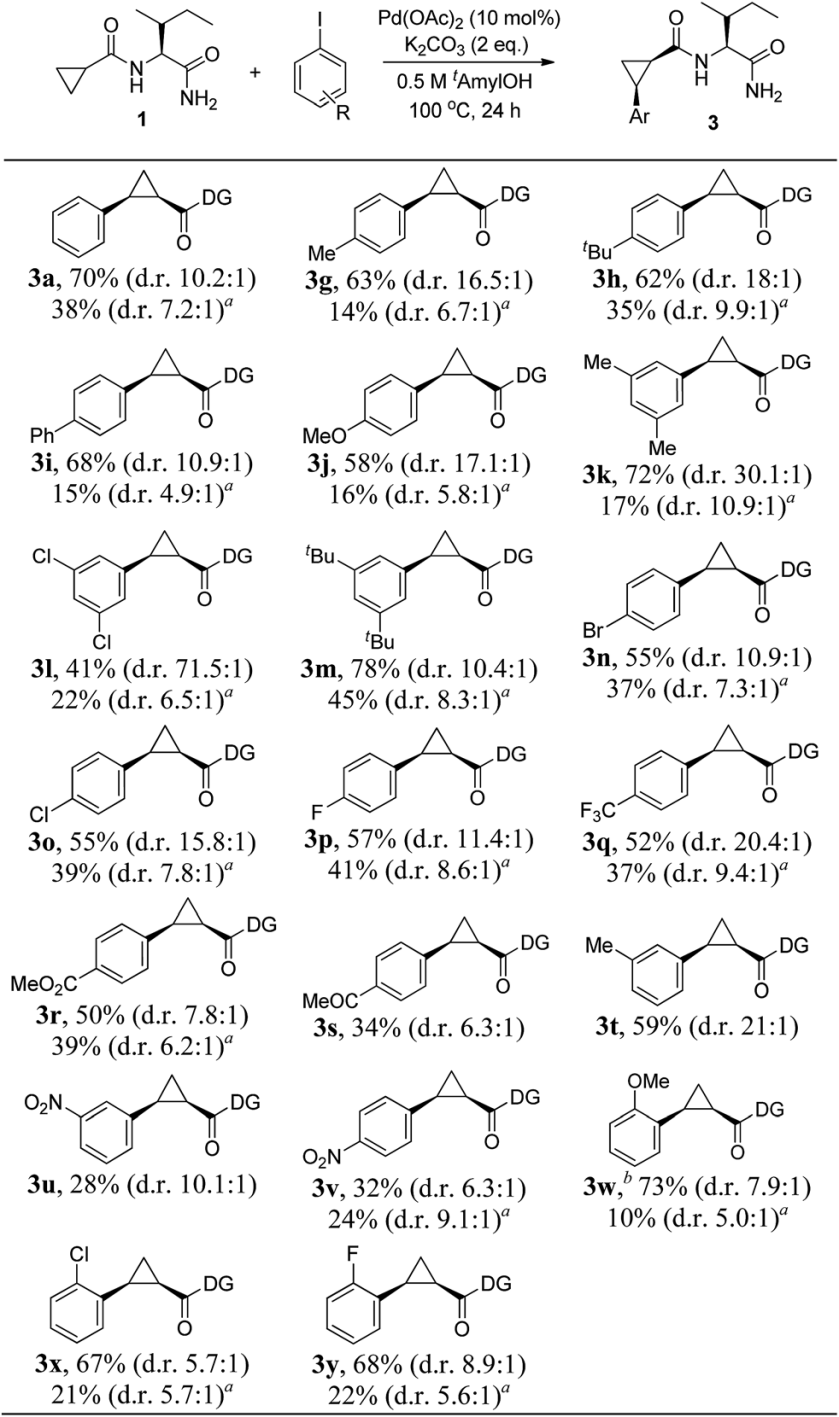
Substrate scope. Substrate (1.0 equiv.), Pd(OAc)_2_ (10 mol%), K_2_CO_3_ (2.0–2.5 equiv.), and Ar–I (1.5–3.0 equiv.) in *t*-amyl-OH (0.5 M) at 100 °C for 24 h: isolated yields of monoarylation products. The d.r was determined by HPLC analysis. In general, mixtures of mono- and diarylation products (4.0–4.6 : 1) were observed by NMR analysis. ^*a*^After 1 h: NMR yield of monoarylation products. ^*b*^120 °C.

Having established that the Pd(ii)-catalyzed highly diastereoselective C–H arylation of cyclopropanes was enabled by the Ile-NH_2_ directing group, the scope of aryl iodides was studied with a range of functional groups such as phenyl, methoxy, ketone, ester, alkyl, halides, and trifluoromethyl, which could be used as a useful synthetic handle for the further transformations. We were delighted to observe that substitution with both electron-donating (Me–, ^*t*^Bu–, and OMe–) and electron-withdrawing groups (F–, Cl–, Br–, CF_3_–, CO_2_Me–, and COMe–) on the aryl iodides were viable under the reaction conditions to afford the corresponding products with high levels of diastereoselectivity, summarized in Scheme 3. In addition, the major isomer could be easily isolated by silica gel chromatography. Outstanding diastereoselectivities could be achieved in the reactions of 3,5-disubstituted iodobenzenes (d.r = 30.1 : 1 for **3k**, d.r = 71.5 : 1 for **3l**). The chemoselective coupling with bromo iodobenzene afforded the synthetically important **3n**, of use for further synthetic elaboration. The position of the substituents in the iodobenzene did not show much change to the reactivity. Notably, aryl iodides with *ortho*-substituents, such as OMe, Cl, and F were also compatible with the reaction conditions to afford the corresponding products (**3w–y**) with good yields (67–73%). Expanding the scope to nitro-substituted iodobenzenes was also possible, but the products were obtained in only around 30% yields (**3u** and **3v**).

**Scheme 4:**
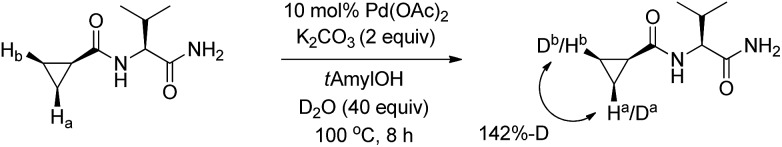
H/D exchange studies.

Although severe peak overlap occurs in the NMR spectra of two diastereotopic hydrogens, a significant level of the di-deuterated product (>40%) was observed when the reaction mixture was treated with D_2_O under the optimized conditions and in the absence of aryl iodide (Scheme 4).^12^ This controlled H/D exchange study implies a facile C–H activation process for both diastereotopic hydrogens of the cyclopropanes.

**Scheme 5:**
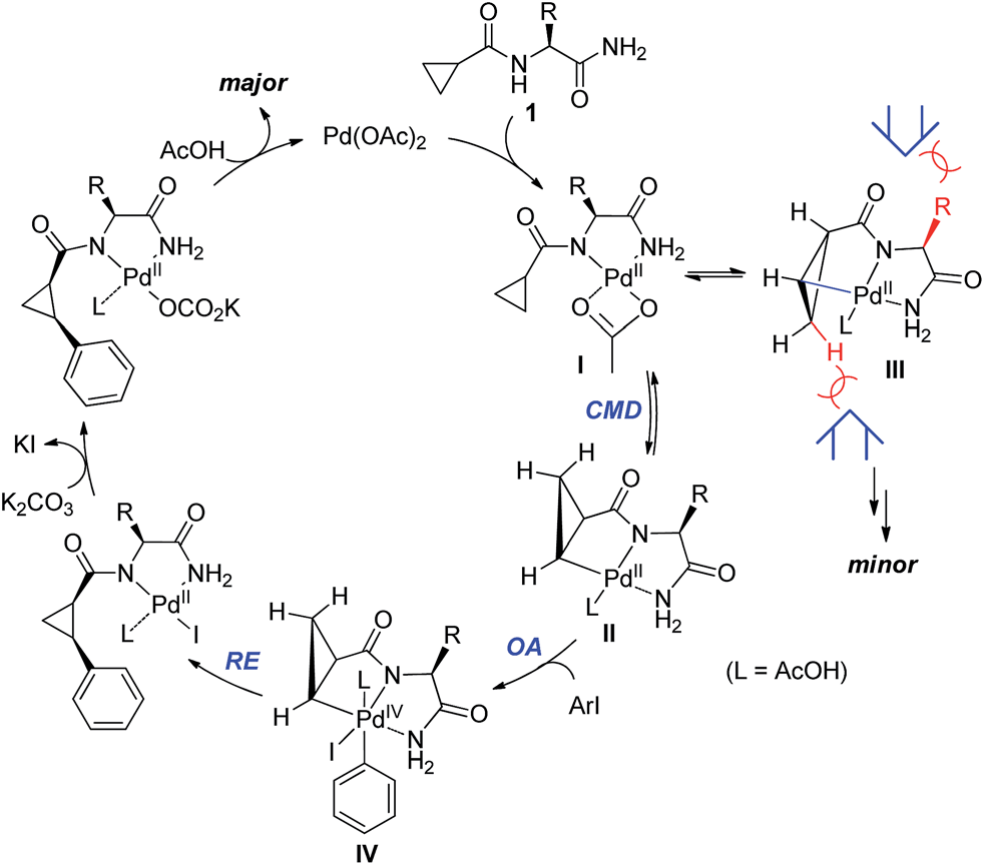
Mechanistic considerations for Pd-catalyzed diastereoselective C–H activation of cyclopropanes.

We proposed one of the possible reaction mechanisms involving Ile-NH_2_ auxiliary-controlled asymmetric C–H functionalization in which a Pd(ii)/Pd(iv)^16^ catalytic cycle is invoked (Scheme 5). The coordination of two nitrogen atoms to the Pd catalyst generates a palladium amide **I**. Subsequently, the cleavage of the C(sp^3^)–H bond on the cyclopropane through a concerted metalation deprotonation (CMD) process produces the palladacycle complexes **II** and **III**, which may exist in equilibrium.^17^ The diastereoselectivity of the arylation could occur either at the C–H activation step or at the oxidative addition step. At this stage, the diastereoselectivity can be assumed to be established at the oxidative addition step under Curtin–Hammett control^18^ because the C–H activation process of both diastereotopic hydrogens is relatively facile (Scheme 4). The oxidative addition of the aryl iodide to **II** affords Pd(iv) intermediate **IV**,^19^ which then undergoes reductive elimination followed by protonation leading to the formation of the arylated product. On the other hand, the oxidative addition process for the insertion of an aryl iodide into Pd complex **III** appears to be less feasible because the R substituent and the methylene group of cyclopropane cause blocking of the top and bottom faces.

**Scheme 6:**
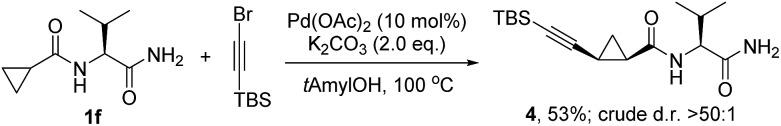
C–H alkynylation.

Furthermore, we preliminarily investigated the diastereoselective C–H alkynylation and observed that **1f** reacted with bromoalkyne under the same reaction conditions,^20^ resulting in the single diastereomer **4** in a 53% yield along with 44% of unreacted starting material (Scheme 6).

## Conclusions

In summary, we have developed the Pd(ii)-catalyzed highly diastereoselective β-methylene C(sp^3^)–H bond activation/C–H arylation of cyclopropanes enabled by the Ile-NH_2_ directing group. This study represents the first systematic investigation of substrate-bound α-amino acid amides as chiral bidentate directing groups in the asymmetric C(sp^3^)–H functionalization of cyclopropanes. The present reactions demonstrated a broad range of substrate scope and allowed for the selective installation of a variety of substituents on cyclopropanes. Further developments to extend this methodology to other substrates and detailed mechanistic studies are underway.

## Supplementary Material

Supplementary information

Crystal structure data
