# Supplementary material for: Asymmetric C–H functionalization of cyclopropanes using an isoleucine-NH2 bidentate directing group
Source: Chem Sci. 2015 Apr 16;6(6):3611–6. doi: 10.1039/c5sc01137j (PMC5659170; doi:10.1039/c5sc01137j)
Supplement: Supplementary file 1 [file SC-006-C5SC01137J-s001.pdf]

Electronic Supplementary Material (ESI) for Chemical Science.
This journal is © The Royal Society of Chemistry 2015

Supporting Information

Asymmetric C–H Functionalization of Cyclopropanes Using Isoleucine-NH_2 Bidentate Directing Group

Jinhee Kim, Mikyung Sim, Namhoon Kim, Sungwoo Hong*

Department of Chemistry, Korea Advance Institute of Science and Technology (KAIST) & Center for Catalytic Hydrocarbon Functionalizations, Institute for Basic Science (IBS), Daejeon 305-701, Korea

General Methods and Materials S2

Experimental Procedure and data S7

^1H- and ^{13}C-NMR Spectra S26

X-Ray-generated images for 2a S57

General Methods and Materials

Commercial grade reagents and solvents were used without further purification except as indicated below. Analytical thin layer chromatography (TLC) was performed on precoated silica gel 60 F^{254} plates and visualization on TLC was achieved by UV light (254 nm) and ninhydrin solution, and heat as developing agents. Flash column chromatography was undertaken on silica gel (400-630 mesh). ^{1}H NMR was recorded on 400 MHz and chemical shifts were quoted in parts per million (ppm) referenced to the appropriate solvent peak or 2.50 ppm for DMSO-d_6. The following abbreviations were used to describe peak splitting patterns when appropriate: s = singlet, d = doublet, t = triplet, q = quartet, h = hexet, m = multiplet, dd = doublet of doublet, td = doublet of triplet, ddd = doublet of doublet of doublet. Coupling constants, *J*, were reported in hertz unit (Hz). ^{13}C NMR was recorded on 100 MHz and was fully decoupled by broad band proton decoupling. Chemical shifts were reported in ppm referenced to the center line of a triplet at 39.5 ppm of DMSO-d_6. Diastereomeric ratios were determined by integration of HPLC. Analytical HPLC was performed with an Agilent 1200 Series HPLC utilizing Poroschell 120 EC-C18 columns (4.6 mm x 50 mm) with visualization at 254 nm. Melting points were determined using an electronthermal IA9000 series melting point apparatus. Mass spectral data were obtained from the KAIST Basic Science Institute by using ESI method.

Experimental Procedure and data

General procedure (GP I) for synthesis of starting materials

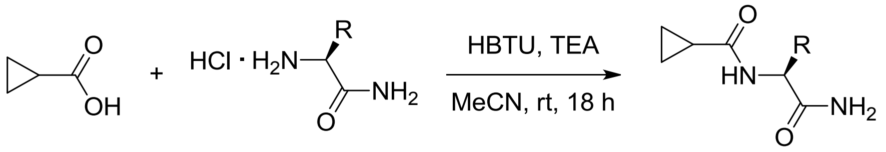

Triethylamine (2.15 equiv.) and HBTU (1.04 equiv.) were added to a solution of cyclopropane carboxylic acid (1.0 equiv.) and amino amide HCl (1.0 equiv.) in acetonitrile (3.5 M). After it was stirred at room temperature for 18 h, the resulting white solid was washed with acetonitrile to give pure amide. (Bach, A.; Eildal, J. N. N.; Stuhr-Hansen, N.; Deeskamp, R.; Gottschalk, M.; , Pedersen, Søren, W.; Kristensen, A. S.; Strømgaard, K. *J. Med. Chem.* **2011**, *54*, 1333.)

Optimization of Reaction Conditions

Initial reaction condition

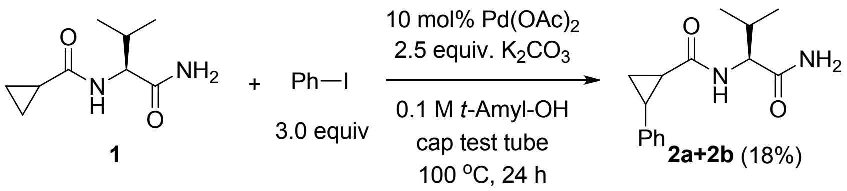

Solvent and Base screening was conducted, but the reaction did not proceeded or only trace amount of product was observed under followed condition (Solvent : Toluene, *t*BuOH, DCE, Dioxane, H_2O, *i*PrOH, DME, HFIP, DMF, DMSO, MeCN, MIBK, nBuOH, THF, 3-Pentanol, 1-pentanol/ Base : Na_2CO_3, Li_2CO_3, Cs_2CO_3, Ag_2CO_3, $CuCO_3.Cu(OH)_2$, $CaCO_3$, $(NH_4)_2CO_3$, $KHCO_3$, $NaHCO_3$, KOAc, KTFA, KOTf, KOtBu, KF, KH_2PO_4, K_3PO_4, CsOAc, $Ba(OAc)_2$, $Co(OAc)_2$, Pyridine, TEA)

Table S1. Concentration Control

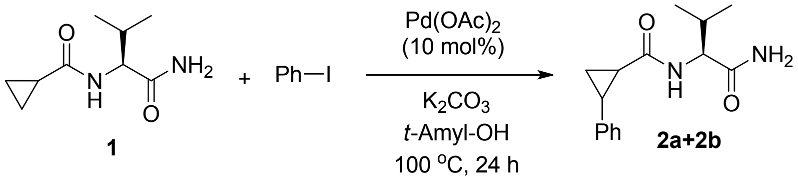

entry	Concentration (M)	Yield (mono + di)
1	0.1	23+ trace%
2	0.25	57 + 6 %
3	0.33	59 + 8 %
**4**	**0.5**	**62 + 10 %**
5	1.0	62 + 13%

[a] Conditions: 0.1 mmol of substrate, 10 mol % $Pd(OAc)_2$, 2.0 equiv of Ph–I, 2.0 equiv of K_2CO_3, *t*-Amyl-OH, 100 °C, cap test tube, 24 h.

Table S2. Equivalent Control

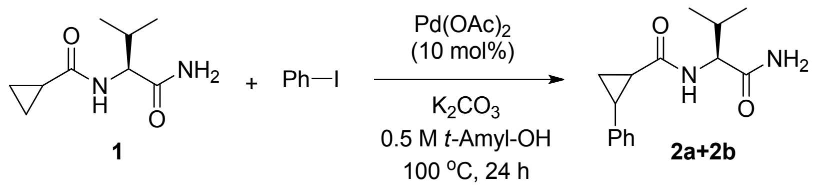

entry	PhI (equiv)	K_2CO_3 (equiv)	Yield (mono + di)
1	4	3.0	46 + 24 %
2	3	3.0	46 + 20 %
3	2	3.0	50 + 17 %
4	2	2.5	57 + 14 %
5	2	2.0	66 + 13 %
6	2	1.5	53 + 7 %
7	2	1.0	49 + 6 %
**9**	**1.5**	**2.0**	**71+16 %**

[a] Conditions: 0.1 mmol of substrate, 10 mol% $Pd(OAc)_2$, Ph–I, K_2CO_3, 0.2 mL of *t*-Amyl-OH, 100 °C, cap test tube, 24 h.

Table S3. Ligand Screening

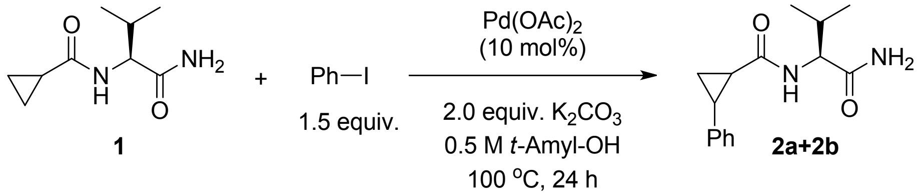

entry	ligand	Conversion (mono + di)	entry	ligand	Conversion (mono + di)
1	PPh_3 (0.2)	56+12 %	11	PivOH (0.2)	54 + 13 %
2	PCy_3 (0.2)	-	12	BzOH (0.2)	57 + 8 %
3	X-Phos (0.2)	trace	13	TFA (0.2)	66 + 11 %
4	DavePhos (0.2)	product ~ sm	14	AdOH (0.2)	-
5	JohnPhos (0.2)	trace	15	$TsOH.H_2O$ (0.2)	product ~ sm
6	pyridine (0.2)	product ~ sm	16	CSA (0.2)	-
7	diMepyr (0.2)	-	17	$(BnO)_2PO_2H$ (0.2)	-
8	Me_2Npyr (0.2)	trace	18	DMSO (0.2)	64%
9	*t*Bu_2pyr (0.2)	67 + 13%	19	$PhSO_2NH_2$ (0.2)	Trace

10	AcOH (0.2)	68 +13 %	20	iPr$_2$S (0.2)	61 + 21 %

[a] Conditions: 0.1 mmol of substrate, 10 mol % Pd(OAc)$_2$, 1.5 equiv of Ph–I, 2.0 equiv of K_2CO_3, 0.2 mL of *t*-Amyl-OH, 100 °C, cap test tube, 24 h. [b] Yield was determined by ^{1}H NMR analysis of the crude product using *p*-anisaldehyde as an internal standard.

Table S4. Reaction Profile[a]

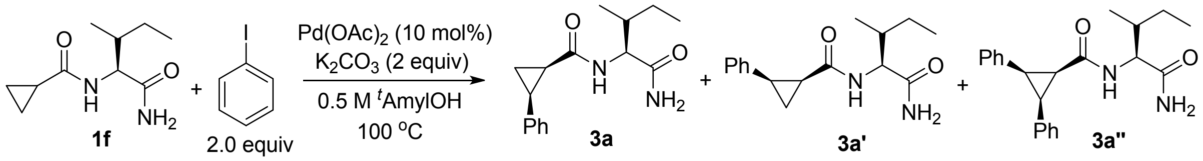

entry	Time	NMR yield[b]			d.r[c]
		3a	3a'	3a''	
1	1 h	33.7%	4.7%	2%	7.2:1
2	2 h	39.5%	5.5%	3%	7.2:1
3	4 h	49.3%	6.7%	7%	7.3:1
4	6 h	57.5%	7.5%	8%	7.7:1
5	9 h	57.4%	7.5%	11%	7.7:1
6	12 h	66.3%	6.7%	16%	9.9:1
7	24 h	66.1%	6.2%	16%	10.7:1

[a] (0.1 mmol), iodobenzene (2.0 equiv), Pd(OAc)$_2$ (10 mol%) and K_2CO_3 (2.0 equiv.) in 0.2 mL of t-Amyl-OH (0.5 M), 100 °C, cap test tube. [b]2-(4-bromophenyl)acetonitrile was used as reference material. [C]diastereomeric ratio was determined by HPLC analysis (C18 column, TFA:MeCN:H_2O = 0.1:17:83)

H/D exchange study

Although two diastereotopic hydrogens do not split each other, significant level of di-deuterated product (>40%) was observed when the reaction mixture was treated with D_2O under the optimized conditions and in the absence of aryl iodide.

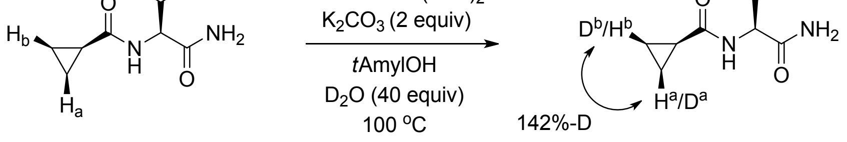

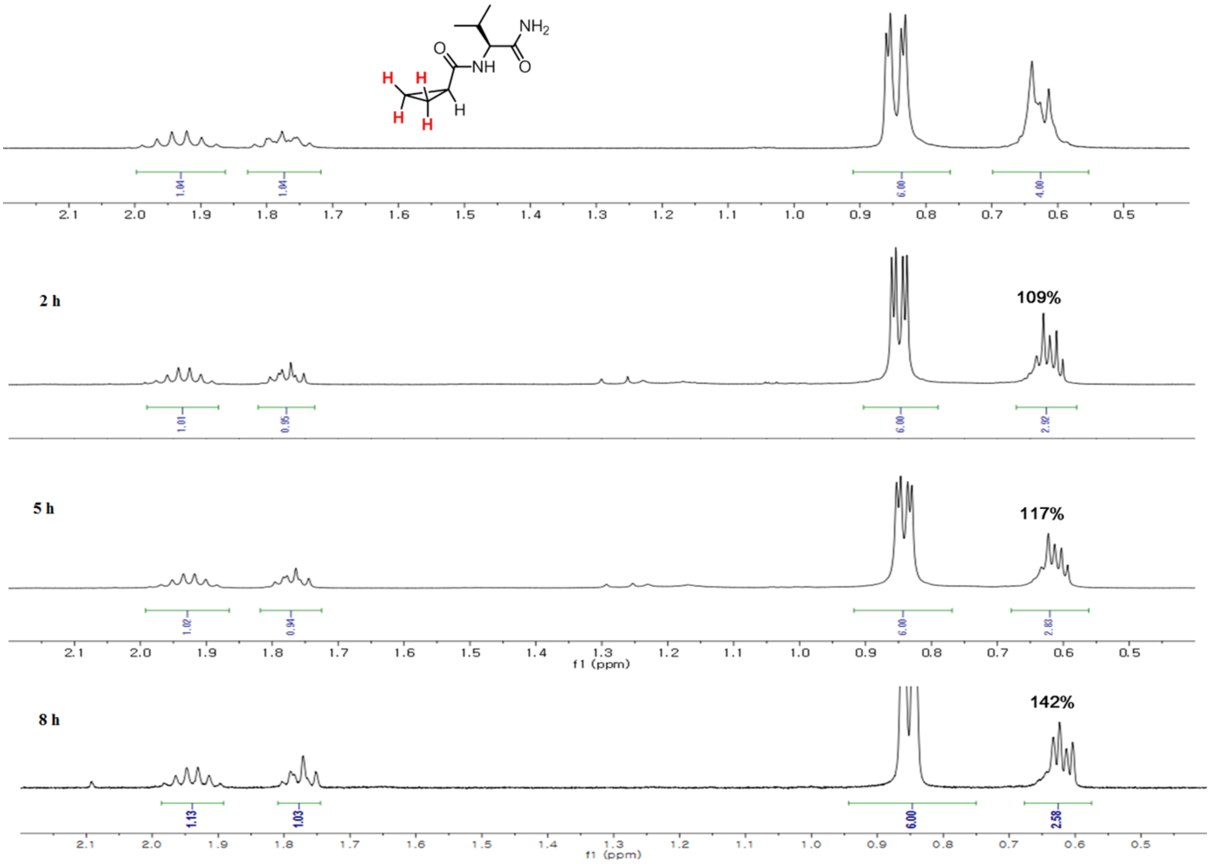

LC data of representative compounds

***cis*-*N*-((2*S*, 3*R*)-1-amino-3-methyl-1-oxopentan-2-yl)-2-phenylcyclopropanecarboxamide**

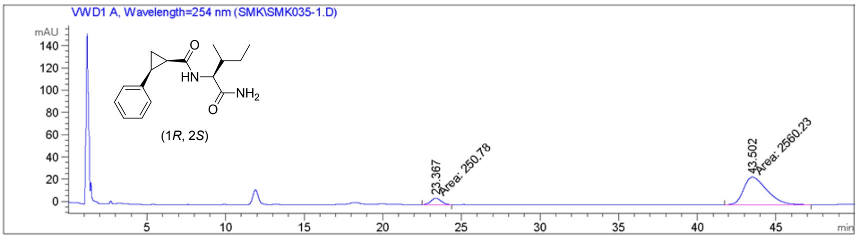

Peak #	RetTime [min]	Type	Width [min]	Area [mAU*s]	Height [mAU]	Area %
1	23.367	MM	0.7393	250.78035	5.65387	8.9214
2	43.502	MM	1.7180	2560.22876	24.83679	91.0786

***cis*-*N*-((2*S*, 3*R*)-1-amino-3-methyl-1-oxopentan-2-yl)-2-(3,5-dimethylphenyl)cyclopropanecarboxamide**

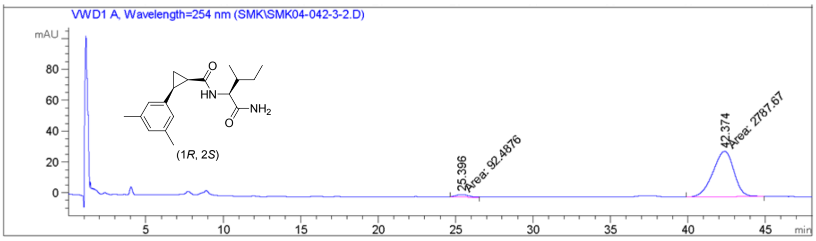

Peak #	RetTime [min]	Type	Width [min]	Area [mAU*s]	Height [mAU]	Area %
1	25.396	MM	0.9158	92.48757	1.68326	3.2112
2	42.374	MM	1.5728	2787.66553	29.53971	96.7888

***cis-N*-((2*S*, 3*R*)-1-amino-3-methyl-1-oxopentan-2-yl)-2-(4-formylphenyl)cyclopropanecarboxamide**

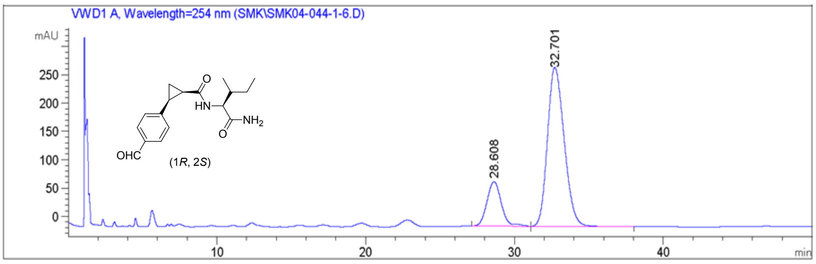

Peak #	RetTime [min]	Type	Width [min]	Area [mAU*s]	Height [mAU]	Area %
1	28.608	BB	1.0328	5233.64014	78.59945	19.2367
2	32.701	BB	1.2207	2.19729e4	281.18927	80.7633

General procedure (GP II) for diastereoselective arylation of cyclopropanes (2a-3z)

The mixture of substrate **1** (0.2 mmol), aryl iodide (1.5-3.0 equiv.), $Pd(OAc)_2$ (0.1 equiv.), K_2CO_3 (2.0-2.5 equiv.) in *tert*-Amylalcohol (0.4 mL) was stirred for 24 h at 100 ^oC. The mixture was monitored by TLC using DCM and MeOH (20:1, 3 times) as the mobile phase. The reaction mixture was cooled to room temperature, filtrated on celite using EtOAc as eluent. After the filtrate was evaporated on reduced pressure, the crude residue was roughly purified by flash chromatography on silica gel (DCM to DCM:MeOH=10:1). The residue was dissolve in DMSO and diastereomeric ratio was determined by HPLC analysis (EC-C18 column, λ = 254 nm). The major diastereomer was separated by flash chromatography on silica gel (DCM:MeOH = 30:1).

***cis-N*-((*S*)-3-methyl-1-(methylamino)-1-oxobutan-2-yl)-2-phenylcyclopropanecarboxamide (2)**

Substrate (0.1 mmol), aryl iodide (3.0 equiv), $Pd(OAc)_2$ (0.1 equiv.), K_2CO_3 (2.5 equiv) in *tert*-Amylalcohol (0.2 mL), 100 °C, 24 h. Yield 26% (d.r. = 8.1:1). The diastereomeric ratio was determined by HPLC analysis on C18 column (TFA:MeCN:H_2O = 0.1:16:84, flow rate 0.5 mL/min, λ = 254 nm, τminor = 17.0 min, τmajor = 26.5 min.). A trace amount of the corresponding minor diastereomer was not isolated.

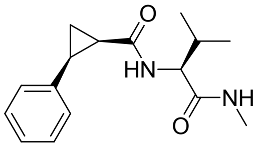

(1*R*, 2*S*)

Yield 23% (6.3 mg). mp 198-200 °C, White solid. ^1H NMR (400 MHz, DMSO-d_6) δ 7.93 (d, *J* = 8.8 Hz, 1H), 7.75 (q, *J* = 4.6 Hz, 1H), 7.20 – 7.11 (m, 4H), 7.14 – 7.05 (m, 1H), 3.80 (dd, *J* = 8.8, 6.8 Hz, 1H), 2.53 (d, *J* = 4.6 Hz, 3H), 2.42 – 2.31 (m, 1H), 2.19 (ddd, *J* = 9.4, 7.8, 5.8 Hz, 1H), 1.76 (h, *J* = 6.8 Hz, 1H), 1.47 (ddd, *J* = 7.2, 5.8, 4.4 Hz, 1H), 1.20 – 1.09 (m, 1H), 0.62 (d, *J* = 6.8 Hz, 3H) 0.58 (d, *J* = 6.8 Hz, 3H). ^{13}C NMR (101 MHz, DMSO-d_6) δ 171.6, 168.3, 137.6, 128.9, 127.3, 125.7, 57.9, 29.9, 25.3, 24.0, 23.1, 18.9, 18.0, 9.3. HRMS (ESI$^+$) m/z calcd. $C_{16}H_{22}N_2NaO_2^+$ $[M+Na]^+$: 297.1573 , found: 297.1577.

***cis N*-((*S*)-1-amino-3-methyl-1-oxobutan-2-yl)-2-phenylcyclopropane-carboxamide (2)**

Substrate (0.3 mmol), aryl iodide (2.0 equiv), $Pd(OAc)_2$ (0.1 equiv), K_2CO_3 (2.0 equiv) in *tert*-Amylalcohol (0.6 mL), 100 °C, 24 h. Yield 71% (d.r. = 8.6:1). The diastereomeric ratio was determined by HPLC analysis on C18 column (TFA:MeCN:H_2O = 0.1:20:80, flow rate 0.5 mL/min, λ = 254 nm, τmajor = 4.1 min, τminor = 5.8 min.) NOE was observed between H^1 and H^2 in both of diastereomers. This result indicates that obtained 2 diastereomers has *cis* configuration. The absolute configuration of major product was confirmed by crystallographic data.

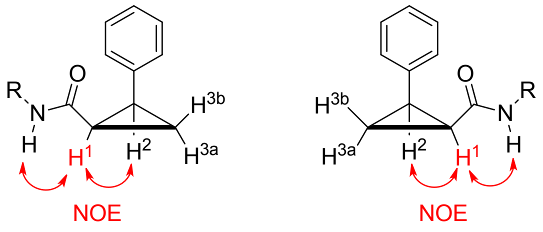

Major ***Minor***

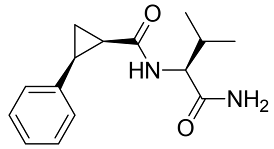

(1*R*, 2*S*)

Yield 63% (49 mg). mp 184-185 °C. White solid. ^{1}H NMR (400 MHz, DMSO-d_6) δ 7.83 (d, *J* = 8.9 Hz, 1H), 7.25 (s, 1H), 7.19 – 7.13 (m, 4H), 7.13 – 7.06 (m, 1H), 6.92 (s, 1H), 3.87 (dd, *J* = 8.9, 6.4 Hz, 1H), 2.42 – 2.31 (m, 1H), 2.20 (ddd, *J* = 9.4, 7.8, 5.7 Hz, 1H), 1.78 (h, *J* = 6.8 Hz, 1H), 1.48 (ddd, *J* = 7.3, 5.7, 4.3 Hz, 1H), 1.15 (ddd, *J* = 8.6, 7.8, 4.3 Hz, 1H), 0.63 (d, *J* = 6.8 Hz, 3H), 0.57 (d, *J* = 6.8 Hz, 3H). ^{13}C NMR (101 MHz, DMSO-d_6) δ 173.6, 168.7, 138.1, 129.4, 127.8, 126.2, 57.9, 30.4, 24.4, 23.6, 19.4, 18.2, 9.7. HRMS (ESI$^+$) m/z calcd. $C_{15}H_{20}N_2NaO_2^+$ [M+Na]$^+$: 283.1417, found: 283.1400.

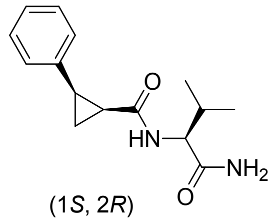

(1*S*, 2*R*)

Minor product. mp 195-197 °C. White solid. ^{1}H NMR (400 MHz, DMSO-d_6) δ 7.89 (d, *J* = 9.2 Hz, 1H), 7.23 – 7.02 (m, 6H), 6.90 (s, 1H), 3.98 (dd, *J* = 9.2, 6.7 Hz, 1H), 2.37 (q, *J* = 8.5 Hz, 1H), 2.27 – 2.16 (m, 1H), 1.88 (h, *J* = 6.7 Hz, 1H), 1.43 (ddd, *J* = 7.2, 5.7, 4.2 Hz, 1H), 1.16 (td, *J* = 8.2, 4.2 Hz, 1H), 0.78 (d, *J* = 6.7 Hz, 3H), 0.78 (d, *J* = 6.7 Hz, 3H). ^{13}C NMR (101 MHz, DMSO-d_6) δ 173.0, 168.7, 137.8, 129.2, 127.5, 125.8, 57.2, 30.5, 24.0, 22.4, 19.3, 17.9, 9.9. HRMS (ESI$^+$) m/z calcd. $C_{15}H_{20}N_2NaO_2^+$ [M+Na]$^+$: 283.1417, found: 283.1397.

(1*s*, 2*R*, 3*S*)-*N*-((*S*)-1-amino-3-methyl-1-oxobutan-2-yl)-2,3-diphenylcyclopropanecarboxamide (2)

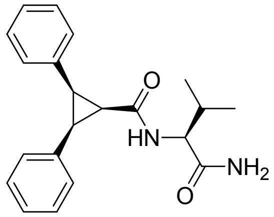

Diarylated product. mp 90-92 °C. White solid. ^{1}H NMR (400 MHz, DMSO-d_6) δ 7.92 (d, *J* = 8.9 Hz, 1H), 7.26 (s, 1H), 7.22 – 7.00 (m, 10H), 7.00 – 6.88 (m, 1H), 3.99 (dd, *J* = 8.9, 6.5 Hz, 1H), 2.91 – 2.76 (m, 2H), 2.59 (t, *J*

= 9.3 Hz, 1H), 1.93 – 1.84 (m, 1H), 0.74 (d, *J* = 6.7 Hz, 6H). ^{13}C NMR (101 MHz, DMSO-d_6) δ 173.1, 168.1, 135.7, 135.3, 131.2, 130.7, 126.8, 126.8, 125.5, 125.5, 57.4, 30.1, 28.2, 28.1, 26.4, 19.2, 17.9. HRMS (ESI$^+$) m/z calcd. $C_{21}H_{24}N_2NaO_2^+$ [M+Na]$^+$: 359.1730, found: 359.1733.

***cis-N*-((2*S*, 3*R*)-1-amino-3-methyl-1-oxopentan-2-yl)-2-phenylcyclopropanecarboxamide (3a)**

Substrate (0.3 mmol), aryl iodide (2.0 equiv), $Pd(OAc)_2$ (0.1 equiv), K_2CO_3 (2.0 equiv) in *tert*-Amylalcohol (0.6 mL), 100 ^{o}C, 24 h. Yield 70% (d.r. = 10.2:1). The diastereomeric ratio was determined by HPLC analysis on C18 column (TFA:MeCN:H_2O = 0.1:17:83, flow rate 0.5 mL/min, λ = 254 nm, τminor = 23.4 min, τmajor = 43.5 min.)

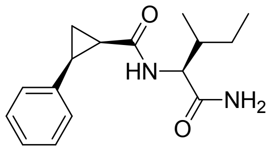

(1*R*, 2*S*)

Yield 64% (52.8 mg). mp 193-195 ^{o}C. White solid**.** ^{1}H NMR (400 MHz, DMSO-d_6) δ 7.85 (d, *J* = 9.0 Hz, 1H), 7.25 (s, 1H), 7.19 – 7.04 (m, 5H), 6.90 (s, 1H), 3.86 (dd, *J* = 9.0, 7.4 Hz, 1H), 2.40 – 2.29 (m, 1H), 2.17 (ddd, *J* = 9.4, 7.8, 5.7 Hz, 1H), 1.57 – 1.42 (m, 2H), 1.23 – 1.08 (m, 2H), 0.90 – 0.77 (m, 1H), 0.62 (t, *J* = 7.4 Hz, 3H), 0.59 (d, *J* = 6.8 Hz, 3H). ^{13}C NMR (101 MHz, DMSO-d_6) δ 173.3, 168.1, 137.6, 128.9, 127.3, 125.7, 56.5, 36.1, 24.0, 24.0, 23.2, 15.2, 10.8, 9.2. HRMS (ESI$^+$) m/z calcd. $C_{16}H_{22}N_2NaO_2^+$ [M+Na]$^+$: 297.1573, found: 297.1574.

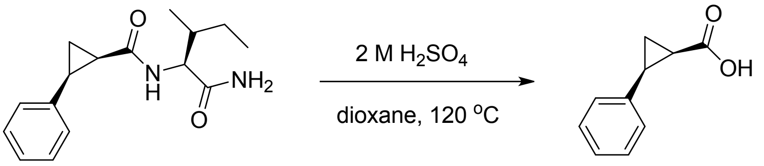

The Suspension of the amide (0.1 mmol) in 4 M H_2SO_4(aq) and 1,4-dioxane (1:1) was heated to 120 ^{o}C for 20 h. The resulting solution was cooled to room temperature, diluted with H_2O and extracted 3 times with ether. The combined organic layer was dried over Mg_2SO_4 and concentrated. The residue was purified by flash chromatography on silica gel (DCM:MeOH = 15:1) to give (1*R*,2*S*)-2-phenylcyclopropanecarboxylic acid (13 mg, 81%). Observed $[\alpha]^D$ = -28.9 ^{o} (c = 0.4, $CHCl_3$, 20 ^{o}C) , lit. for >98% ee [α] ^{D} = -28 ^{o} (c = 1.02, $CHCl_3$) (Elling, G. R.; Hahn, R. C.; Schwab, G. *J. Am. Chem. Soc.* **1973**, *95*, 5659.)

***cis-N*-((*S*)-1-amino-3,3-dimethyl-1-oxobutan-2-yl)-2-phenylcyclopropanecarboxamide (3b)**

Substrate (0.3 mmol), aryl iodide (2.0 equiv), $Pd(OAc)_2$ (0.1 equiv), K_2CO_3 (2.0 equiv) in *tert*-Amylalcohol (0.6 mL), 100 °C, 24 h. Yield 49%. (d.r. = 13.7:1). The diastereomeric ratio was determined by HPLC analysis on C18 column (TFA:MeCN:H_2O = 0.1:20:80, flow rate 0.5 mL/min, λ = 254 nm, τminor = 12.3 min, τmajor = 23.0 min.)

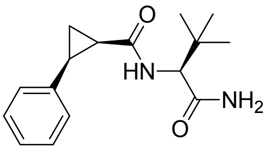

(1*R*, 2*S*)

Yield 46% (38.2 mg). mp 178-180°C. White solid. ^{1}H NMR (400 MHz, DMSO-d_6) δ 7.75 (d, *J* = 9.5 Hz, 1H), 7.33 (s, 1H), 7.22 – 7.10 (m, 4H), 7.13 – 7.03 (m, 1H), 6.93 (s, 1H), 3.92 (d, *J* = 9.5 Hz, 1H), 2.42 – 2.31 (m, 1H), 2.27 (ddd, *J* = 9.4, 7.8, 5.8 Hz, 1H), 1.49 (ddd, *J* = 7.2, 5.8, 4.3 Hz, 1H), 1.14 (td, *J* = 8.1, 4.3 Hz, 1H), 0.68 (s, 9H). ^{13}C NMR (101 MHz, DMSO-d_6) δ 172.4, 168.1, 137.6, 129.0, 127.3, 125.7, 59.6, 33.3, 26.5, 24.1, 23.2, 9.2. HRMS (ESI$^+$) m/z calcd. $C_{16}H_{22}N_2NaO_2^+$ [M+Na]$^+$: 297.1573, found: 297.1581.

***cis-N*-((2*S*, 3*S*)-1-amino-3-(benzyloxy)-1-oxobutan-2-yl)-2-phenylcyclopropanecarboxamide (3c)**

Substrate (0.1 mmol), aryl iodide (5.0 equiv), $Pd(OAc)_2$ (0.1 equiv), K_2CO_3 (3.0 equiv) in *tert*-Amylalcohol (0.6 mL), 100 °C, 6 h. Yield 46%. (d.r. = 6.2:1) The diastereomeric ratio was determined by HPLC analysis on C18 column (TFA:MeCN:H_2O = 0.1:20:80, flow rate 0.5 mL/min, λ = 254 nm, τminor = 53.8 min, τmajor = 58.9 min.)

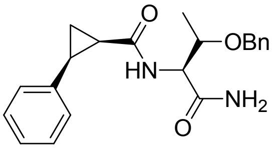

(1*R*, 2*S*)

Yield 40% (13.8 mg). colorless gum. ^{1}H NMR (400 MHz, DMSO-d_6) δ 7.84 (d, *J* = 9.1 Hz, 1H), 7.37 – 7.23 (m, 5H), 7.22 – 7.04 (m, 7H), 4.43 (d, *J* = 12.0 Hz, 1H), 4.37 (d, *J* = 12.0 Hz, 1H), 4.11 (dd, *J* = 9.1, 3.4 Hz, 1H), 3.74 (qd, *J* = 6.2, 3.1 Hz, 1H), 2.45 – 2.27 (m, 2H), 1.50 (ddd, *J* = 7.1, 5.7, 4.2 Hz, 1H), 1.17 (td, *J* = 8.2, 4.2 Hz, 1H), 0.71 (d, *J* = 6.2 Hz, 3H). ^{13}C NMR (101 MHz, DMSO-d_6) δ 172.1, 168.6, 138.7, 137.5, 128.9, 128.1, 127.4, 127.3, 127.2, 125.8, 74.5, 70.1, 56.5, 24.1, 23.3, 15.8, 9.4. HRMS (ESI$^+$) m/z calcd. $C_{21}H_{24}N_2NaO_3^+$ [M+Na]$^+$:

375.1679, found: 375.1681.

***cis-N*-((*S*)-2-amino-2-oxo-1-phenylethyl)-2-phenylcyclopropanecarboxamide (3d)**

Substrate (0.1 mmol), aryl iodide (2.0 equiv), $Pd(OAc)_2$ (0.1 equiv), K_2CO_3 (2.0 equiv) in *tert*-Amylalcohol (0.2 mL), 100 °C, 24 h. Yield 56% (d.r. = 3.2:1). The diastereomeric ratio was determined by HPLC analysis on C18 column (TFA:MeCN:H_2O = 0.1:15:85, flow rate 0.5 mL/min, λ = 254 nm, τminor = 43.9 min, τmajor = 68.1 min.)

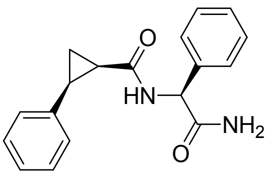

(1*R*,2*S*)

0.1 mmol scale. Yield 42% (12.3 mg). mp 178-180 °C. White solid. ^1H NMR (400 MHz, DMSO-d_6) δ 8.46 (d, *J* = 8.0 Hz, 1H), 7.61 (s, 1H), 7.27 – 7.20 (m, 3H), 7.17 – 7.15 (m, 2H), 7.13 – 7.03 (m, 6H), 5.19 (d, *J* = 8.0 Hz, 1H), 2.41 – 2.32 (m, 1H), 2.30 – 2.25 (m, 1H), 1.48 –1.44 (m, 1H), 1.20 – 1.15 (m, 1H). ^{13}C NMR (101 MHz, DMSO-d_6) δ 171.8, 168.3, 138.8, 137.5, 129.0, 128.0, 127.4, 127.1, 126.8, 125.8, 56.1, 24.1, 22.7, 9.5 . HRMS (ESI^+) m/z calcd. $C_{18}H_{18}N_2NaO_2^+$ $[M+Na]^+$: 317.1260, found: 317.1232.

***cis-N*-((*S*)-2-amino-1-cyclohexyl-2-oxoethyl)-2-phenylcyclopropanecarboxamide (3e)**

Substrate (0.3 mmol), aryl iodide (2.0 equiv), $Pd(OAc)_2$ (0.1 equiv), K_2CO_3 (2.0 equiv) in *tert*-Amylalcohol (0.6 mL), 100 °C, 24 h. Yield 55% (d.r. = 8.8:1). The diastereomeric ratio was determined by HPLC analysis on C18 column (TFA:MeCN:H_2O = 0.1:20:80, flow rate 0.5 mL/min, λ = 254 nm, τminor = 14.3 min, τmajor = 21.9 min.)

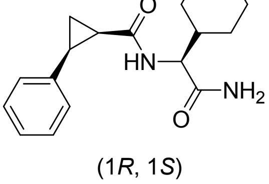

(1*R*, 1*S*)

Yield 49% (44.3 mg). mp 244-245°C. White solid. ^1H NMR (400 MHz, DMSO-d_6) δ 7.81 (d, *J* = 8.9 Hz, 1H),

7.27 (s, 1H), 7.22 – 7.11 (m, 4H), 7.14 – 7.06 (m, 1H), 6.94 (s, 1H), 3.89 (dd, J = 8.9, 6.7 Hz, 1H), 2.41 – 2.30 (m, 1H), 2.19 (ddd, J = 9.4, 7.7, 5.7 Hz, 1H), 1.58 – 1.44 (m, 4H), 1.45 – 1.21 (m, 3H), 1.13 (td, J = 8.1, 4.4 Hz, 1H), 1.09 – 0.88 (m, 3H), 0.74 – 0.55 (m, 2H). ^{13}C NMR (101 MHz, DMSO-d_6) δ 173.0, 168.0, 137.6, 128.9, 127.3, 125.7, 56.8, 28.9, 27.6, 25.7, 25.5, 23.9, 23.4, 9.0. HRMS (ESI$^+$) m/z calcd. $C_{18}H_{24}N_2NaO_2^+$ [M+Na]$^+$: 323.1730, found: 323.1737.

***cis*-*N*-((*S*)-1-amino-1-oxo-3-phenylpropan-2-yl)-2-phenylcyclopropanecarboxamide (3f)**

Substrate (0.3 mmol), aryl iodide (2.0 equiv), $Pd(OAc)_2$ (0.1 equiv), K_2CO_3 (2.0 equiv) in *tert*-Amylalcohol (0.6 mL), 100 °C, 24 h. Yield 54%. (d.r. = 3.4:1) The diastereomeric ratio was determined by HPLC analysis on C18 column (TFA:MeCN:H_2O = 0.1:25:75, flow rate 0.5 mL/min, λ = 254 nm, τminor = 26.0 min, τmajor = 42.4 min.)

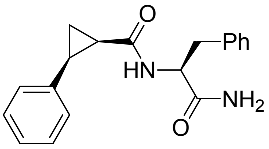

(1*R*, 2*S*)

Yield 41% (36.7 mg). mp 216-218 °C. White solid. ^1H NMR (400 MHz, DMSO-d_6) δ 8.16 (d, J = 8.1 Hz, 1H), 7.34 (s, 1H), 7.29 – 7.18 (m, 3H), 7.15 – 7.08 (m, 2H), 7.08 – 7.01 (m, 3H), 6.98 (s, 1H), 6.92 – 6.83 (m, 2H), 4.24 (ddd, J = 9.6, 8.1, 4.6 Hz, 1H), 2.84 (dd, J = 13.9, 4.6 Hz, 1H), 2.67 (dd, J = 13.9, 9.6 Hz, 1H), 2.34 – 2.22 (m, 1H), 2.06 (ddd, J = 9.2, 7.8, 5.6 Hz, 1H), 1.34 (ddd, J = 7.2, 5.6, 4.1 Hz, 1H), 1.13 (ddd, J = 8.8, 7.8, 4.1 Hz, 1H). ^{13}C NMR (101 MHz, DMSO-d_6) δ 173.4, 168.6, 138.1, 137.7, 129.0, 129.0, 128.0, 127.4, 126.1, 125.6, 53.8, 37.4, 24.0, 22.1, 9.9. HRMS (ESI$^+$) m/z calcd. $C_{19}H_{20}N_2NaO_2^+$ [M+Na]$^+$: 331.1417, found: 331.1413.

***cis*-*N*-((*S*)-1-amino-3-methyl-1-oxopentan-2-yl)-2-(*p*-tolyl)cyclopropanecarboxamide (3g)**

Substrate (0.2 mmol), aryl iodide (1.5 equiv), $Pd(OAc)_2$ (0.1 equiv), K_2CO_3 (2.0 equiv) in *tert*-Amylalcohol (0.4 mL), 100 °C, 24 h. Yield 63% (d.r. = 16.5:1). The diastereomeric ratio was determined by HPLC analysis on C18 column (TFA:MeCN:H_2O = 0.1:30:70, flow rate 0.5 mL/min, λ = 254 nm, τminor = 7.34 min, τmajor = 10.2 min.)

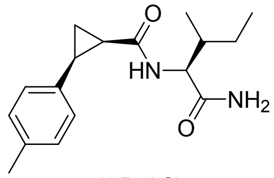

(1*R*, 2*S*)

Yield 59% (34 mg). mp 202-203 °C. White solid. ^{1}H NMR (400 MHz, DMSO-d_6) δ 7.81 (d, *J* = 8.9 Hz, 1H), 7.24 (s, 1H), 7.04 (d, *J* = 7.8 Hz, 2H), 6.96 (d, *J* = 7.7 Hz, 2H), 6.90 (s, 1H), 3.85 (t, *J* = 8.2 Hz, 1H), 2.33 – 2.27 (m, 1H), 2.21 (s, 3H), 2.13 (ddd, *J* = 9.6, 7.6, 5.7 Hz, 1H), 1.55 – 1.38 (m, 2H), 1.32 – 1.06 (m, 2H), 0.91 – 0.77 (m, 1H), 0.69 – 0.58 (m, 6H). ^{13}C NMR (101 MHz, DMSO-d_6) δ 173.8, 168.7, 135.0, 135.0, 129.3, 128.4, 57.1, 36.6, 24.5, 24.1, 23.5, 21.1, 15.7, 9.6 HRMS (ESI$^+$) m/z calcd. $C_{17}H_{24}N_2NaO_2^+$ [M+Na]$^+$: 311.1730, found: 311.1707.

***cis*-*N*-((2*S*, 3*R*)-1-amino-3-methyl-1-oxopentan-2-yl)-2-(4-(tert-butyl)phenyl)cyclopropanecarboxamide(3h)**

Substrate (0.2 mmol), aryl iodide (2.0 equiv), Pd(OAc)$_2$ (0.1 equiv), K_2CO_3 (2.0 equiv) in *tert*-Amylalcohol (0.4 mL), 100 °C, 24 h. Yield 62% (d.r. = 18:1) The diastereomeric ratio was determined by HPLC analysis on C18 column (TFA:MeCN:H_2O = 0.1:40:60, flow rate 0.5 mL/min, λ = 254 nm, τminor = 10.0 min, τmajor = 12.7 min.)

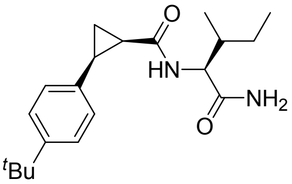

(1*R*, 2*S*)

Yield 58% (38.5 mg). mp 198-199 °C. White solid. ^{1}H NMR (400 MHz, DMSO-d_6) δ 7.80 (d, *J* = 9.0 Hz, 1H), 7.26 (s, 1H), 7.17 (d, *J* = 8.2 Hz, 2H), 7.08 (d, *J* = 8.2 Hz, 2H), 6.92 (s, 1H), 3.88 (dd, *J* = 9.0, 7.2 Hz, 1H), 2.34 – 2.27 (m, 1H), 2.15 (ddd, *J* = 9.5, 7.7, 5.7 Hz, 1H), 1.54 – 1.42 (m, 2H), 1.23 (s, 9H), 1.20 – 1.05 (m, 2H), 0.80 (ddd, *J* = 13.6, 9.0, 7.2 Hz, 1H), 0.61 (t, *J* = 7.4 Hz, 3H), 0.55 (d, *J* = 6.8 Hz, 3H). ^{13}C NMR (101 MHz, DMSO-d_6) δ 173.7, 168.4, 148.3, 134.8, 128.9, 124.5, 56.9, 36.6, 34.4, 31.6, 24.4, 23.9, 23.8, 15.6, 11.2, 9.4. HRMS (ESI$^+$) m/z calcd. $C_{20}H_{30}N_2NaO_2^+$ [M+Na]$^+$: 353.2199, found: 353.2211.

***cis*-*N*-((*S*)-1-amino-3-methyl-1-oxopentan-2-yl) -2 -([1,1'-biphenyl]-4-yl)cyclopropanecarboxamide (3i)**

Substrate (0.2 mmol), aryl iodide (1.5 equiv), $Pd(OAc)_2$ (0.1 equiv), K_2CO_3 (2.0 equiv) in *tert*-Amylalcohol (0.4 mL), 100 °C, 24 h. Yield 68% (d.r. = 10.9:1). The diastereomeric ratio was determined by HPLC analysis on C18 column (TFA:MeCN:H_2O = 0.1:40:60, flow rate 0.5 mL/min, λ = 254 nm, τminor = 7.8 min, τmajor = 8.7 min.)

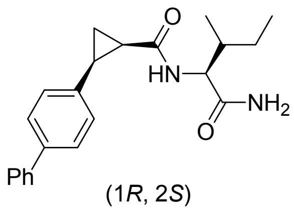

(1*R*, 2*S*)

Yield 63% (44 mg). mp 217 °C. White solid. ^{1}H NMR (400 MHz, DMSO-d_6) δ 7.88 (d, *J* = 8.9 Hz, 1H), 7.59 (d, *J* = 7.7 Hz, 2H), 7.50 – 7.39 (m, 4H), 7.37 – 7.22 (m, 4H), 6.91 (s, 1H), 3.88 (t, *J* = 8.2 Hz, 1H), 2.43 – 2.37 (m, 1H), 2.22 (td, *J* = 8.4, 5.7 Hz, 1H), 1.55 – 1.47 (m, 2H), 1.23 – 1.11 (m, 2H), 0.92 – 0.76 (m, 1H), 0.62 – 0.58 (m, 6H). ^{13}C NMR (101 MHz, DMSO-d_6) δ 173.2, 168.0, 140.1, 137.6, 137.0, 129.4, 128.9, 127.1, 126.3, 125.6, 56.6, 40.1, 39.9, 39.7, 39.5, 39.3, 39.1, 38.9, 36.1, 24.0, 23.6, 23.4, 15.2, 10.7, 9.3. HRMS (ESI$^+$) m/z calcd. $C_{22}H_{26}N_2NaO_2^+$ [M+Na]$^+$: 373.1886, found: 373.1875.

***cis*-*N*-((*S*)-1-amino-3-methyl-1-oxopentan-2-yl)-2-(4-methoxyphenyl)cyclopropanecarboxamide (3j)**

Substrate (0.2 mmol), aryl iodide (1.5 equiv), $Pd(OAc)_2$ (0.1 equiv), K_2CO_3 (2.0 equiv) in *tert*-Amylalcohol (0.4 mL), 100 °C, 24 h. Yield 58% (d.r. = 17.1:1) The diastereomeric ratio was determined by HPLC analysis on C18 column (TFA:MeCN:H_2O = 0.1:20:80, flow rate 0.5 mL/min, λ = 254 nm, τminor = 4.5 min, τmajor = 5.1 min.)

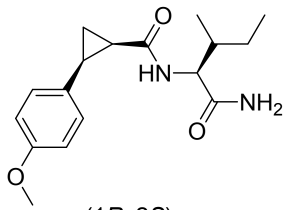

(1*R*, 2*S*)

Yield 55% (33.6 mg). mp 186-187 °C. White solid. ^{1}H NMR (400 MHz, DMSO-d_6) δ 7.82 (d, *J* = 8.9 Hz, 1H), 7.26 (s, 1H), 7.07 (d, *J* = 8.4 Hz, 2H), 6.91 (s, 1H), 6.73 (d, *J* = 8.4 Hz, 2H), 3.88 (dd, *J* = 8.9, 7.2 Hz, 1H), 3.68 (s, 3H), 2.32 – 2.26 (m, 1H), 2.11 (ddd, *J* = 9.7, 7.7, 5.7 Hz, 1H), 1.57 – 1.38 (m, 2H), 1.26 – 1.05 (m, 2H), 0.92

– 0.78 (m, 1H), 0.69 – 0.59 (m, 6H). ^{13}C NMR (101 MHz, DMSO-d_6) δ 173.3, 168.2, 157.5, 129.9, 129.4, 112.8, 56.6, 54.9, 36.2, 24.1, 23.3, 23.0, 15.2, 10.8, 9.2. HRMS (ESI$^+$) m/z calcd. $C_{17}H_{24}N_2NaO_3^+$ [M+Na]$^+$: 327.1679, found: 327.1678.

***cis*-*N*-((2*S*, 3*R*)-1-amino-3-methyl-1-oxopentan-2-yl)-2-(3,5-dimethylphenyl)cyclopropanecarboxamide (3k)**

Substrate (0.2 mmol), aryl iodide (3.0 equiv), $Pd(OAc)_2$ (0.1 equiv), K_2CO_3 (2.5 equiv) in *tert*-Amylalcohol (0.4 mL), 100 °C, 24 h. Yield 72% (d.r. = 30.1:1). The diastereomeric ratio was determined by HPLC analysis on C18 column (TFA:MeCN:H_2O = 0.1:25:75, flow rate 0.5 mL/min, λ = 254 nm, τminor = 25.4 min, τmajor = 42.4 min.)

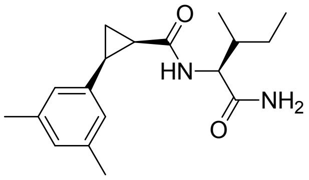

(1*R*, 2*S*)

Yield 69% (41.8 mg). mp 184-185 °C. White solid. ^1H NMR (400 MHz, DMSO-d_6) δ 7.85 (d, *J* = 8.9 Hz, 1H), 7.27 (s, 1H), 6.93 (s, 1H), 6.76 (s, 2H), 6.73 (s, 1H), 3.91 (dd, *J* = 8.9, 7.2 Hz, 1H), 2.33 – 2.22(m, 1H), 2.17-2.10 (m, 7H), 1.62 – 1.47 (m, 1H), 1.41 (ddd, *J* = 7.1, 5.6, 4.1 Hz, 1H), 1.31 – 1.21 (m, 1H), 1.08 (td, *J* = 8.1, 4.2 Hz, 1H), 0.96 – 0.82 (m, 1H), 0.75 – 0.59 (m, 6H). ^{13}C NMR (101 MHz, DMSO-d_6) δ 173.3, 168.2, 137.5, 136.0, 127.3, 126.9, 56.6, 36.3, 24.1, 23.8, 22.6, 20.9, 15.2, 11.0, 9.1. HRMS (ESI$^+$) m/z calcd. : $C_{18}H_{26}N_2NaO_2^+$ [M+Na]$^+$: 325.1886, found: 325.1886.

***cis*-*N*-((2*S*, 3*R*)-1-amino-3-methyl-1-oxopentan-2-yl)-2-(3,5-dichlorophenyl)cyclopropanecarboxamide (3l)**

Substrate (0.2 mmol), aryl iodide (1.5 equiv), $Pd(OAc)_2$ (0.1 equiv), K_2CO_3 (2.0 equiv) in *tert*-Amylalcohol (0.4 mL), 100 °C, 24 h. Yield 41% (d.r. = 71.5:1) The diastereomeric ratio was determined by HPLC analysis on C18 column (TFA:MeCN:H_2O = 0.1:30:70, flow rate 0.5 mL/min, λ = 254 nm, τminor = 16.3 min, τmajor = 28.8 min.)

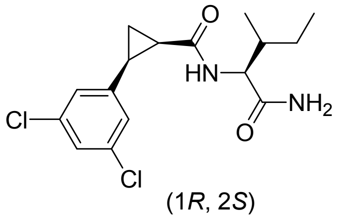

(1*R*, 2*S*)

Yield 40% (27.5 mg). mp 212-214 °C. White solid. ^{1}H NMR (400 MHz, DMSO-d_6) δ 8.01 (d, *J* = 9.0 Hz, 1H), 7.34-7.33 (m, 2H), 7.18 (d, *J* = 2.0 Hz, 2H), 6.93 (s, 1H), 3.92 (dd, *J* = 9.0, 7.4 Hz, 1H), 2.44 – 2.38 (m, 1H), 2.24 (ddd, *J* = 9.5, 7.7, 5.7 Hz, 1H), 1.59 – 1.44 (m, 2H), 1.23 – 1.15 (m, 2H), 0.89 – 0.82 (m, 1H), 0.71 – 0.56 (m, 6H). ^{13}C NMR (101 MHz, DMSO-d_6) δ 173.5, 168.1, 142.8, 133.4, 128.2, 125.9, 56.9, 36.6, 24.5, 23.9, 23.6, 15.5, 11.2, 10.0. HRMS (ESI$^+$) m/z calcd. $C_{16}H_{20}Cl_2N_2NaO_2^+$ [M+Na]$^+$: 365.0794, found: 365.0799.

***cis-N*-((2*S*, 3*R*)-1-amino-3-methyl-1-oxopentan-2-yl)-2-(3,5-di-*tert*-butylphenyl)cyclopropanecarboxamide (3m)**

Substrate (0.2 mmol), aryl iodide (3.0 equiv), $Pd(OAc)_2$ (0.1 equiv), K_2CO_3 (2.5 equiv) in *tert*-Amylalcohol (0.4 mL), 100 °C, 24 h. Yield 78% (d.r. = 10.4:1). The diastereomeric ratio was determined by HPLC analysis on C18 column (TFA:MeCN:H_2O = 0.1:50:50, flow rate 0.5 mL/min, λ = 254 nm, τminor = 12.7 min, τmajor = 16.2 min.)

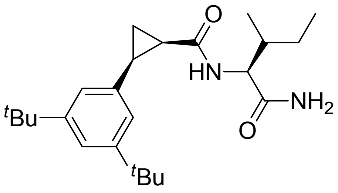

(1*R*, 2*S*)

Yield 72% (55 mg). mp 62-64 °C. White solid. ^{1}H NMR (400 MHz, DMSO-d_6) δ 7.71 (d, *J* = 9.0 Hz, 1H), 7.25 (s, 1H), 7.13 (t, J = 1.8 Hz, 1H), 6.98 (d, *J* = 1.8 Hz, 2H), 6.92 (s, 1H), 3.93 (dd, *J* = 9.0, 6.8 Hz, 1H), 2.51 – 2.49 (m, 1H), 2.17 (ddd, *J* = 9.4, 7.8, 5.7 Hz, 1H), 1.54 – 1.41 (m, 2H), 1.24 (s, 18H), 1.20 – 1.07 (m, 2H), 0.80 – 0.73 (m, 1H), 0.59 (t, *J* = 7.3 Hz, 3H), 0.51 (d, *J* = 6.7 Hz, 3H). ^{13}C NMR (101 MHz, DMSO-d_6) δ 173.6, 168.5, 149.3, 136.8, 123.1, 119.8, 57.0, 36.8, 34.7, 31.7, 25.1, 24.6, 24.1, 15.5, 11.5, 9.9. HRMS (ESI$^+$) m/z calcd. $C_{24}H_{38}N_2NaO_2^+$ [M+Na]$^+$: 409.2825, found: 409.2877.

***cis-N*-((*S*)-1-amino-3-methyl-1-oxopentan-2-yl)-2-(4-bromophenyl)cyclopropanecarboxamide (3n)**

Substrate (0.2 mmol), aryl iodide (1.5 equiv), $Pd(OAc)_2$ (0.1 equiv), K_2CO_3 (2.0 equiv) in *tert*-Amylalcohol (0.4 mL), 100 °C, 24 h. Yield 55% (d.r. =10.9:1) The diastereomeric ratio was determined by HPLC analysis on C18 column (TFA:MeCN:H_2O = 0.1:40:60, flow rate 0.5 mL/min, λ = 254 nm, τminor = 4.10 min, τmajor = 4.94 min.)

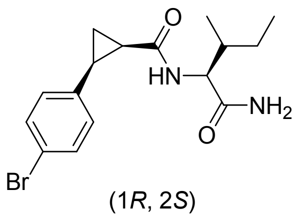

(1*R*, 2*S*)

Yield 51% (35 mg). mp 214-215 °C. White solid. ^1H NMR (400 MHz, DMSO-d_6) δ 7.89 (d, *J* = 8.9 Hz, 1H), 7.34 (d, *J* = 8.2 Hz, 2H), 7.31 – 7.25 (m, 1H), 7.11 (d, *J* = 8.2 Hz, 2H), 6.91 (s, 1H), 3.87 (dd, *J* = 8.9, 7.5 Hz, 1H), 2.37 – 2.31 (m, 1H), 2.19 (ddd, *J* = 9.8, 7.7, 5.6 Hz, 1H), 1.53 – 1.45 (m, 2H), 0.90 – 0.74 (m, 1H), 0.67 – 0.57 (m, 6H). ^{13}C NMR (101 MHz, DMSO-d_6) δ 173.2, 167.9, 137.2, 131.1, 130.1, 118.8, 56.5, 36.1, 24.0, 23.4, 23.3, 15.2, 10.7, 9.3. HRMS (ESI$^+$) m/z calcd. $C_{16}H_{21}BrN_2NaO_2^+$ [M+Na]$^+$: 375.0679, found: 375.0688.

***cis*-*N*-((2*S*, 3*R*)-1-amino-3-methyl-1-oxopentan-2-yl)-2-(4-chlorophenyl)cyclopropanecarboxamide (3o)**

Substrate (0.2 mmol), aryl iodide (2.5 equiv), $Pd(OAc)_2$ (0.1 equiv), K_2CO_3 (2.0 equiv) in *tert*-Amylalcohol (0.4 mL), 100 °C, 24 h. Yield 55% (d.r. = 15.8:1) The diastereomeric ratio was determined by HPLC analysis on C18 column (TFA:MeCN:H_2O = 0.1:20:80 to 0.1:30:70, flow rate 0.5 mL/min, λ = 254 nm, τminor = 36.4 min, τmajor = 69.5 min.)

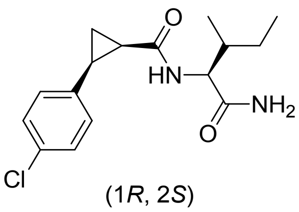

(1*R*, 2*S*)

Yield 52% (32 mg). mp 217-218 °C. White solid. ^1H NMR (400 MHz, DMSO-d_6) δ 7.90 (d, *J* = 9.0 Hz, 1H), 7.29 (s, 1H), 7.21 (d, *J* = 8.6 Hz, 2H), 7.17 (d, *J* = 8.6 Hz, 2H), 6.92 (s, 1H), 3.87 (dd, *J* = 9.0, 7.5 Hz, 1H), 2.39 – 2.33 (m, 1H), 2.19 (ddd, *J* = 9.4, 7.8, 5.7 Hz, 1H), 1.55 – 1.43 (m, 2H), 1.21 – 1.06 (m, 2H), 0.90 – 0.74 (m, 1H), 0.67 – 0.54 (m, 6H). ^{13}C NMR (101 MHz, DMSO-d_6) δ 173.7, 168.3, 137.2, 131.1, 130.8, 127.64, 57.0, 36.5, 24.4, 23.8, 23.7, 15.6, 11.2, 9.8. HRMS (ESI$^+$) m/z calcd. $C_{16}H_{21}ClN_2NaO_2^+$ [M+Na]$^+$: 331.1184, found:

331.1171.

***cis*-N-((2*S*, 3*R*)-1-amino-3-methyl-1-oxopentan-2-yl)-2-(4-fluorophenyl)cyclopropanecarboxamide (3p)**

Substrate (0.2 mmol), aryl iodide (3.0 equiv), $Pd(OAc)_2$ (0.1 equiv), K_2CO_3 (2.5 equiv) in *tert*-Amylalcohol (0.4 mL), 100 °C, 24 h. Yield 57% (d.r. = 11.4:1). The diastereomeric ratio was determined by HPLC analysis on C18 column (TFA:MeCN:H_2O = 0.1:20:80, flow rate 0.5 mL/min, λ = 254 nm, τminor = 10.9 min, τmajor = 21.4 min.)

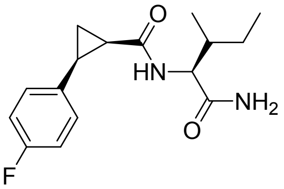

(1*R*, 2*S*)

Yield 53% (31 mg). mp 206-207 °C. White solid. ^{1}H NMR (400 MHz, DMSO-d_6) δ 7.88 (d, *J* = 9.0 Hz, 1H), 7.28 (s, 1H), 7.22 – 7.14 (m, 2H), 7.04 – 6.93 (m, 2H), 6.92 (s, 1H), 3.88 (dd, *J* = 9.0, 7.4 Hz, 1H), 2.38 – 2.32 (m, 1H), 2.17 (ddd, *J* = 9.4, 7.8, 5.7 Hz, 1H), 1.57 – 1.42 (m, 2H), 1.20 – 1.08 (m, 2H), 0.91 – 0.77 (m, 1H), 0.68 – 0.56 (m, 6H). ^{13}C NMR (101 MHz, DMSO-d_6) δ 173.7 , 168.4 , 161.1 (d, *J* = 241 Hz), 134.1 (d, *J* = 3 Hz), 131.03 (d, *J* = 8 Hz), 114.39 (d, *J* = 21 Hz), 57.0, 36.5, 24.4, 23.6, 15.6, 11.2, 9.8. HRMS (ESI$^+$) m/z calcd. $C_{16}H_{21}FN_2NaO_2^+$ $[M+Na]^+$: 315.1479, found: 315.1465.

***cis*-*N*-((2*S*, 3*R*)-1-amino-3-methyl-1-oxopentan-2-yl)-2-(4-(trifluoromethyl)phenyl)cyclopropanecarbox-amide (3q)**

Substrate (0.2 mmol), aryl iodide (2.0 equiv), $Pd(OAc)_2$ (0.1 equiv), K_2CO_3 (2.0 equiv) in *tert*-Amylalcohol (0.4 mL), 100 °C, 24 h. Yield 52% (d.r. = 20.4:1) The diastereomeric ratio was determined by HPLC analysis on C18 column (TFA:MeCN:H_2O = 0.1:25:75, flow rate 0.5 mL/min, λ = 254 nm, τminor = 31.0 min, τmajor = 51.9 min.)

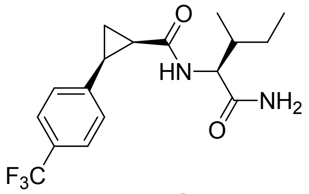

(1*R*, 2*S*)

Yield 50% (34 mg). mp 202-203 °C. White solid. ^{1}H NMR (400 MHz, DMSO-d_6) δ 7.94 (d, *J* = 9.0 Hz, 1H), 7.51 (d, *J* = 7.8 Hz, 2H), 7.37 (d, *J* = 7.8 Hz, 2H), 7.30 (s, 1H), 6.93 (s, 1H), 3.86 (dd, *J* = 9.0, 7.5 Hz, 1H), 2.48 – 2.43 (m, 1H), 2.27 (ddd, *J* = 9.5, 7.8, 5.8 Hz, 1H), 1.58 (ddd, *J* = 7.2, 5.8, 4.5 Hz, 1H), 1.50 – 1.42 (m, 1H), 1.22 (td, *J* = 8.1, 4.5 Hz, 1H), 1.13 – 1.03 (m, 1H), 0.83 – 0.68 (m, 1H), 0.61 – 0.50 (m, 6H). ^{13}C NMR (101 MHz, DMSO-d_6) δ 173.6, 168.1, 143.2, 130.0, 126.9 (q, *J* = 31 Hz), 124.9 (q, *J* = 272 Hz), 124.5 (q, *J* = 4 Hz), 56.9, 36.5, 24.3, 24.1, 15.5, 11.0, 9.8. HRMS (ESI$^+$) m/z calcd. $C_{17}H_{21}F_3N_2NaO_2^+$ [M+Na]$^+$: 365.1447, found: 365.1433.

methyl 4-(*cis*-2-(((2*S*, 3*R*)-1-amino-3-methyl-1-oxopentan-2-yl)carbamoyl)cyclopropyl)benzoate (3r)

Substrate (0.2 mmol), aryl iodide (2.0 equiv), Pd(OAc)$_2$ (0.1 equiv), K_2CO_3 (2.0 equiv) in *tert*-Amylalcohol (0.4 mL), 100 °C, 24 h. Yield 50% (d.r. = 7.8:1). The diastereomeric ratio was determined by HPLC analysis on C18 column (TFA:MeCN:H_2O = 0.1:15:85 to 0.1:20:80, flow rate 0.5 mL/min, λ = 254 nm, τminor = 51.1 min, τmajor = 56.9 min.)

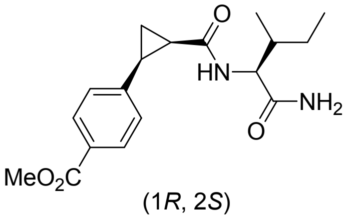

(1*R*, 2*S*)

Yield 44% (26.9 mg). mp 186-187 °C. White solid. ^{1}H NMR (400 MHz, DMSO-d_6) δ 7.93 (d, *J* = 9.0 Hz, 1H), 7.77 (d, *J* = 8.3 Hz, 1H), 7.31 – 7.29 (m, 3H), 6.92 (s, 1H), 3.85 (dd, *J* = 9.0, 7.5 Hz, 1H), 3.81 (s, 3H), 2.51 – 2.49 (m, 1H), 2.27 (ddd, *J* = 9.5, 7.7, 5.8 Hz, 1H), 1.56 (ddd, *J* = 7.2, 5.8, 4.4 Hz, 1H), 1.51 – 1.44 (m, 1H), 1.24 – 1.19 (m, 1H), 1.15 – 1.05 (m, 1H), 0.86 – 0.70 (m, 1H), 0.60 – 0.56 (m, 6H). ^{13}C NMR (101 MHz, DMSO-d_6) δ 173.6, 168.2, 166.7, 144.2, 129.6, 128.7, 127.5, 57.0, 52.4, 36.5, 24.4, 24.3, 15.6, 11.1, 10.0. HRMS (ESI$^+$) m/z calcd. $C_{18}H_{24}N_2NaO_4^+$ [M+Na]$^+$: 355.1628, found: 355.1638.

***cis*-2-(4-acetylphenyl)-*N*-((2*S*, 3*R*)-1-amino-3-methyl-1-oxopentan-2-yl)cyclopropanecarboxamide (3s)**

Substrate (0.2 mmol), aryl iodide (1.5 equiv), $Pd(OAc)_2$ (0.1 equiv), K_2CO_3 (2.0 equiv) in *tert*-Amylalcohol (0.4 mL), 100 °C, 24 h. Yield 34% (d.r. = 6.3:1) The diastereomeric ratio was determined by HPLC analysis on C18 column (TFA:IPA:H_2O = 0.1:10:90, flow rate 0.5 mL/min, λ = 254 nm, τminor = 12.1 min, τmajor = 14.1 min.)

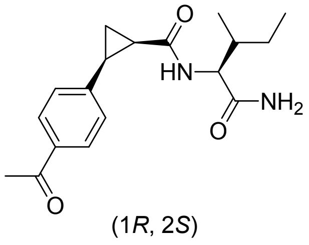

(1*R*, 2*S*)

Yield 30% (18.7 mg). mp 197-198 °C. White solid. ^1H NMR (400 MHz, Methanol-d_4) δ 7.89 – 7.81 (m, 2H), 7.39 – 7.32 (m, 2H), 3.95 (d, *J* = 7.5 Hz, 1H), 2.60 – 2.53 (m, 4H), 2.28 (ddd, *J* = 9.5, 7.8, 5.8 Hz, 1H), 1.75 (ddd, *J* = 7.3, 5.8, 5.1 Hz, 1H), 1.64 – 1.57 (m, 1H), 1.32 (ddd, *J* = 8.5, 7.8, 5.1 Hz, 1H), 1.28 – 1.16 (m, 1H), 0.94 – 0.81 (m, 1H), 0.74 – 0.64 (m, 6H). ^{13}C NMR (101 MHz, Methanol-d_4) δ 200.2, 176.5, 171.3, 145.0, 136.5, 130.3, 129.0, 58.8, 37.8, 26.6, 25.6, 25.5, 25.3, 15.7, 11.3, 10.3. HRMS (ESI$^+$) m/z calcd. $C_{18}H_{24}N_2NaO_3^+$ $[M+Na]^+$: 339.1679, found: 339.1678.

***cis*-*N*-((2*S*, 3*R*)-1-amino-3-methyl-1-oxopentan-2-yl)-2-(m-tolyl)cyclopropanecarboxamide (3t)**

Substrate (0.2 mmol), aryl iodide (2.0 equiv), $Pd(OAc)_2$ (0.1 equiv), K_2CO_3 (2.0 equiv) in *tert*-Amylalcohol (0.4 mL), 100 °C, 24 h. Yield 59% (d.r. = 17.9 :1). The diastereomeric ratio was determined by HPLC analysis on C18 column (TFA:MeCN:H_2O = 0.1:25:75, flow rate 0.5 mL/min, λ = 254 nm, τminor = 10.0 min, τmajor = 12.7 min.)

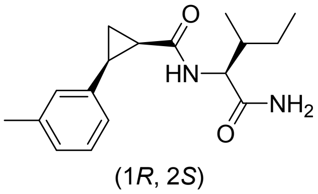

(1*R*, 2*S*)

Yield 57% (33 mg). mp 172-173 °C. White solid. ^1H NMR (400 MHz, DMSO-d_6) δ 7.85 (d, *J* = 9.0 Hz, 1H), 7.27 (s, 1H), 7.05 (t, *J* = 7.5 Hz, 1H), 7.00 – 6.87 (m, 4H), 3.89 (dd, *J* = 9.0, 7.3 Hz, 1H), 2.36 – 2.26 (m, 1H), 2.21 (s, 3H), 2.15 (ddd, *J* = 9.4, 7.7, 5.7 Hz, 1H), 1.56 – 1.50 (m, 1H), 1.44 (ddd, *J* = 7.2, 5.7, 4.2 Hz, 1H), 1.27

– 1.17 (m, 1H), 1.14 – 1.09 (m, 1H), 0.95 – 0.79 (m, 1H), 0.70 – 0.58 (m, 6H). ^{13}C NMR (101 MHz, DMSO-d_6) δ 173.7, 168.58, 138.0, 136.6, 130.1, 127.7, 126.9, 126.5, 57.0, 36.6, 24.5, 24.3, 23.3, 21.4, 15.6, 11.3, 9.6. HRMS (ESI$^+$) m/z calcd. $C_{17}H_{24}N_2NaO_2^+$ [M+Na]$^+$: 311.1730, found: 311.1725.

***cis*-*N*-((2*S*, 3*R*)-1-amino-3-methyl-1-oxopentan-2-yl)-2-(3-nitrophenyl)cyclopropanecarboxamide (3u)**

Substrate (0.2 mmol), aryl iodide (3.0 equiv), $Pd(OAc)_2$ (0.1 equiv), K_2CO_3 (2.5 equiv) in *tert*-Amylalcohol (0.4 mL), 100 °C, 24 h. Yield 28% (d.r. 10.1:1). The diastereomeric ratio was determined by HPLC analysis on C18 column (TFA:MeCN:H_2O = 0.1:25:75, flow rate 0.5 mL/min, λ = 254 nm, τminor = 16.2 min, τmajor = 26.4 min.)

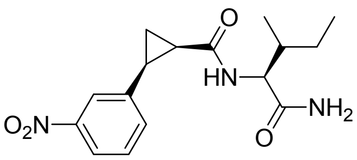

(1*R*, 2*S*)

Yield 25% (15.9 mg). mp 185-187 °C. White solid. ^{1}H NMR (400 MHz, DMSO-d_6) δ 8.06 – 7.96 (m, 3H), 7.64 (d, *J* = 7.7 Hz, 1H), 7.49 (td, *J* = 7.7, 0.8 Hz, 1H), 7.30 (s, 1H), 6.92 (s, 1H), 3.86 (dd, *J* = 9.0, 7.5 Hz, 1H), 2.58 – 2.52 (m, 1H), 2.29 (ddd, *J* = 9.4, 7.7, 5.7 Hz, 1H), 1.59 (ddd, *J* = 7.2, 5.7, 4.6 Hz, 1H), 1.51 – 1.43 (m, 1H), 1.26 (td, *J* = 8.1, 4.6 Hz, 1H), 1.13 – 1.05 (m, 1H), 0.84 – 0.68 (m, 1H), 0.60 – 0.49 (m, 6H). ^{13}C NMR (101 MHz, DMSO-d_6) δ 173.5, 168.2, 147.6, 140.7, 136.3, 129.2, 123.9, 121.2, 56.9, 36.5, 24.4, 24.0, 23.8, 15.5, 11.0, 10.0. HRMS (ESI$^+$) m/z calcd. $C_{16}H_{21}N_3NaO_4^+$ [M+Na]$^+$: 342.1424, found: 342.1417.

***cis*-*N*-((2*S*, 3*R*)-1-amino-3-methyl-1-oxopentan-2-yl)-2-(4-nitrophenyl)cyclopropanecarboxamide (3v)**

Substrate (0.2 mmol), aryl iodide (3.0 equiv), $Pd(OAc)_2$ (0.1 equiv), K_2CO_3 (2.5 equiv) in *tert*-Amylalcohol (0.4 mL), 100 °C, 24 h. Yield 32% (d.r. = 6.3:1). The diastereomeric ratio was determined by HPLC analysis on C18 column (TFA:MeCN:H_2O = 0.1:25:75, flow rate 0.5 mL/min, λ = 254 nm, τminor = 16.0 min, τmajor = 23.9 min.)

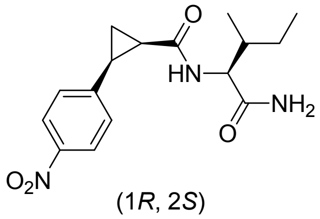

(1*R*, 2*S*)

Yield 28% (17.8 mg). mp 197-198 °C. brown solid. ^{1}H NMR (400 MHz, Methanol-d_4) δ 8.13 – 8.04 (m, 2H), 7.50 – 7.41 (m, 2H), 3.95 (d, *J* = 7.6 Hz, 1H), 2.65 – 2.59 (m, 1H), 2.33 (ddd, *J* = 9.5, 7.8, 5.8 Hz, 1H), 1.78 (ddd, *J* = 7.3, 5.8, 5.1 Hz, 1H), 1.65 – 1.58 (m, 1H), 1.38 (ddd, *J* = 8.5, 7.9, 5.1 Hz, 1H), 1.26 – 1.16 (m, 1H), 0.97 – 0.80 (m, 1H), 0.74 – 0.65 (m, 6H). ^{13}C NMR (101 MHz, Methanol-d_4) δ 176.4, 171.1, 147.9, 147.1, 131.1, 123.79, 58.7, 37.7, 25.6, 25.5, 25.4, 15.7, 11.2, 10.7. HRMS (ESI$^+$) m/z calcd. $C_{16}H_{21}N_3NaO_4^+$ [M+Na]$^+$: 342.1424, found: 342.1395.

***cis*-*N*-((2*S*, 3*R*)-1-amino-3-methyl-1-oxopentan-2-yl)-2-(2-methoxyphenyl)cyclopropanecarboxamide (3w)**

Substrate (0.2 mmol), aryl iodide (3.0 equiv), $Pd(OAc)_2$ (0.1 equiv), K_2CO_3 (2.5 equiv) in *tert*-Amylalcohol (0.4 mL), 120 °C, 24 h. Yield 73% (d.r. = 7.9:1) The diastereomeric ratio was determined by HPLC analysis on C18 column (TFA:MeCN:H_2O = 0.1:20:80, flow rate 0.5 mL/min, λ = 254 nm, τminor = 19.9 min, τmajor = 24.9 min.)

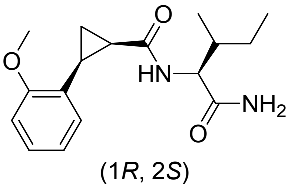

(1*R*, 2*S*)

Yield 65% (40 mg). mp 168-170 °C. White solid. ^{1}H NMR (400 MHz, DMSO-d_6) δ 7.64 (d, *J* = 9.0 Hz, 1H), 7.20 (s, 1H), 7.12 – 7.08 (m, 1H), 7.04 (dd, *J* = 7.5, 1.1 Hz, 1H), 6.90 (s, 1H), 6.83 (dd, *J* = 8.3, 1.1 Hz, 1H), 6.76 (td, *J* = 7.5, 1.1 Hz, 1H), 3.84 (dd, *J* = 9.0, 7.3 Hz, 1H), 3.74 (s, 3H), 2.32 – 2.25 (m, 1H), 2.17 (ddd, *J* = 9.3, 7.8, 5.5 Hz, 1H), 1.58 – 1.39 (m, 1H), 1.28 – 1.16 (m, 1H), 1.10 (td, *J* = 8.1, 4.2 Hz, 1H), 0.92 – 0.79 (m, 1H), 0.66 (t, *J* = 7.4 Hz, 3H), 0.60 (d, *J* = 6.8 Hz, 3H). ^{13}C NMR (101 MHz, DMSO-d_6) δ 173.7, 168.9, 159.0, 129.9, 127.4, 125.8, 119.7, 110.1, 57.1, 55.5, 36.7, 24.4, 22.7, 20.2, 15.6, 11.4, 9.1. HRMS (ESI$^+$) m/z calcd. $C_{17}H_{24}N_2NaO_3^+$ [M+Na]$^+$: 327.1679, found: 327.1687.

***cis-N*-((2*S*, 3*R*)-1-amino-3-methyl-1-oxopentan-2-yl)-2-(2-chlorophenyl)cyclopropanecarboxamide (3x)**

Substrate (0.2 mmol), aryl iodide (3.0 equiv), $Pd(OAc)_2$ (0.1 equiv), K_2CO_3 (2.5 equiv) in *tert*-Amylalcohol (0.4 mL), 100 °C, 24 h. Yield 67% (d.r. = 5.7:1) The diastereomeric ratio was determined by HPLC analysis on C18 column (TFA:MeCN:H_2O = 0.1:25:75, flow rate 0.5 mL/min, λ = 254 nm, τminor = 11.3 min, τmajor = 19.0 min.)

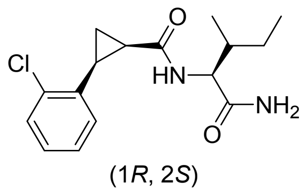

(1*R*, 2*S*)

Yield 57% (35.4 mg). mp 149-151 °C. White solid. ^{1}H NMR (400 MHz, DMSO-d_6) δ 7.88 (d, *J* = 8.9 Hz, 1H), 7.33 – 7.19 (m, 3H), 7.21 – 7.10 (m, 2H), 6.91 (s, 1H), 3.86 (dd, *J* = 8.9, 7.5 Hz, 1H), 2.42 – 2.27 (m, 2H), 1.61 – 1.51 (m, 2H), 1.34 – 1.17 (m, 2H), 1.02 – 0.91 (m, 1H), 0.73 – 0.58 (m, 6H). ^{13}C NMR (101 MHz, DMSO-d_6) δ 173.7, 168.7, 135.8, 135.4, 131.4, 128.6, 127.9, 126.4, 57.0, 36.6, 24.5, 23.4, 22.7, 15.7, 11.2, 9.9. HRMS (ESI$^+$) m/z calcd $C_{16}H_{21}ClN_2NaO_2^+$ [M+Na]$^+$: 331.1184, found: 331.1189.

***cis-N*-((2*S*, 3*R*)-1-amino-3-methyl-1-oxopentan-2-yl)-2-(2-fluorophenyl)cyclopropanecarboxamide (3y)**

Substrate (0.2 mmol), aryl iodide (3.0 equiv), $Pd(OAc)_2$ (0.1 equiv), K_2CO_3 (2.5 equiv) in *tert*-Amylalcohol (0.4 mL), 100 °C, 24 h. Yield 68% (d.r. = 8.9:1). The diastereomeric ratio was determined by HPLC analysis on C18 column (TFA:MeCN:H_2O = 0.1:25:75, flow rate 0.5 mL/min, λ = 254 nm, τminor = 16.4 min, τmajor = 28.7 min.)

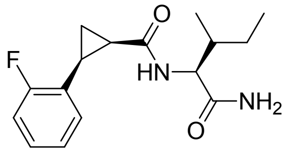

(1*R*, 2*S*)

Yield 62% (35.7 mg). mp 179-181 °C. White solid. ^{1}H NMR (400 MHz, DMSO-d_6) δ 7.92 (d, *J* = 9.0 Hz, 1H), 7.25 (s, 1H), 7.21 – 7.11 (m, 2H), 7.04 – 6.96 (m, 2H), 6.92 (s, 1H), 3.88 (dd, *J* = 9.0, 7.5 Hz, 1H), 2.38 – 2.21 (m, 2H), 1.60 – 1.53 (m, 1H), 1.48 (ddd, *J* = 7.3, 5.7, 4.3 Hz, 1H), 1.29 – 1.16 (m, 2H), 0.99 – 0.88 (m, 1H), 0.71 – 0.61 (m, 6H). ^{13}C NMR (101 MHz, DMSO-d_6) δ 173.7, 168.7, 162.4 (d, *J* = 244 Hz), 131.4 (d, *J* = 4 Hz),

128.1 (d, J = 8 Hz), 125.2 (d, J = 15 Hz), 123.6 (d, J = 3 Hz), 114.6 (d, J = 22 Hz), 57.0, 36.5, 24.4, 22.3, 18.5 (d, J = 4 Hz), 15.6, 11.2, 9.2. HRMS (ESI$^+$) m/z calcd. $C_{16}H_{21}FN_2NaO_2^+$ [M+Na]$^+$: 315.1479, found: 315.1478.

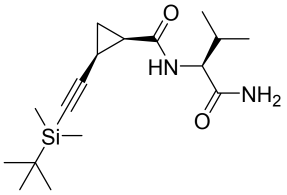

(1*R*, 2*S*)

***cis*-*N*-((*S*)-1-amino-3-methyl-1-oxobutan-2-yl)-2-((*tert*-butyldimethylsilyl)ethynyl)cyclopropane-1-carboxamide (3z)**

Substrate (0.1 mmol), (bromoethynyl)(tert-butyl)dimethylsilane (5.0equiv), $Pd(OAc)_2$ (0.1 equiv), K_2CO_3 (2.0 equiv) in *tert*-Amylalcohol (0.2 mL), 100 ^oC, 24 h. Yield 55. Colorless oil. ^1H NMR (400 MHz, DMSO-d_6) δ 7.97 (d, J = 9.2 Hz, 1H), 7.42 (s, 1H), 7.00 (s, 1H), 4.22 (dd, J = 9.2, 6.3 Hz, 1H), 2.14 – 2.09 (m, 1H), 1.94 (dd, J = 13.3, 6.7 Hz, 1H), 1.78 –1.72 (m, 1H), 1.18 (m, 1H), 1.08 – 1.02 (m, 1H), 0.87 – 0.83 (m, 15H), -0.02(m, 6H). 13C NMR (101 MHz, DMSO-d_6) 173.1,167.4, 106.4, 80.3, 57.3, 30.4, 25.9, 22.2, 19.4, 18.0, 16.2, 12.5, 8.7, -4.5, -4.5. HRMS (ESI$^+$) m/z calcd. $C_{17}H_{30}N_2NaO_2Si^+$ [M+Na]$^+$: 345.1969, found: 345.1980.

Appendix Ⅰ

Spectral Copies of ^{1}H and ^{13}C NMR Data

Obtained in this Study

(1*R*, 2*S*)-*N*-((*S*)-3-methyl-1-(methylamino)-1-oxobutan-2-yl)-2-phenylcyclopropanecarboxamide

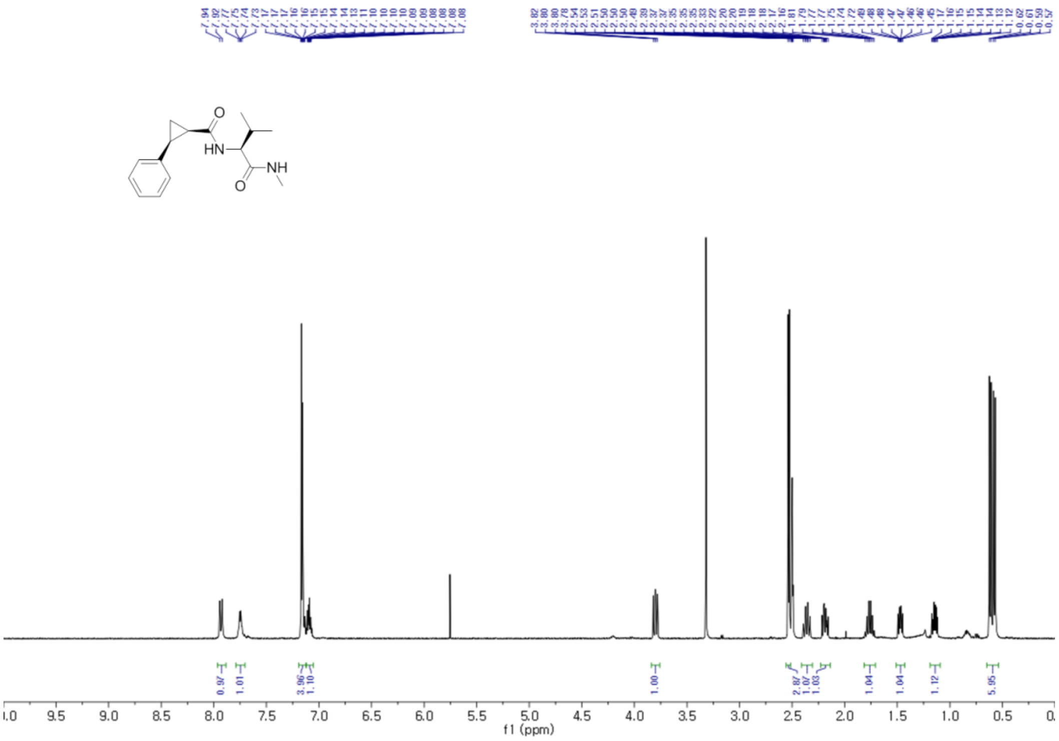

400 MHz, ^{1}H NMR in DMSO-d_6

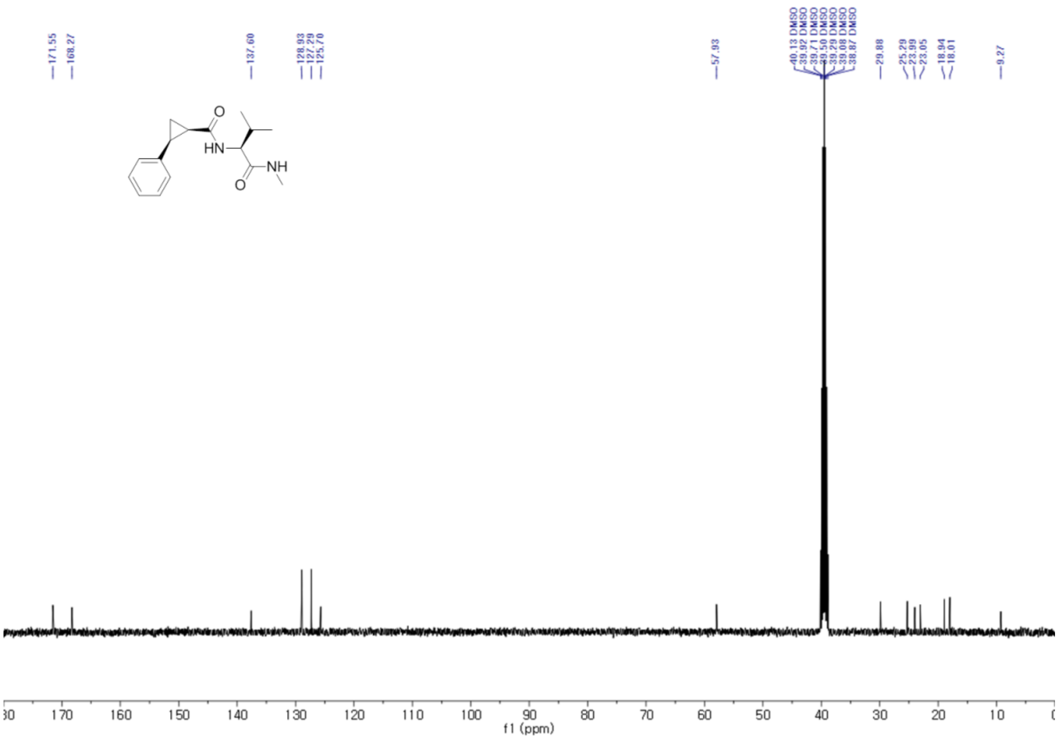

100 MHz, ^{13}C NMR in DMSO-d_6

(1*R*, 2*S*)-*N*-((*S*)-1-amino-3-methyl-1-oxobutan-2-yl)-2-phenylcyclopropanecarboxamide (2a)

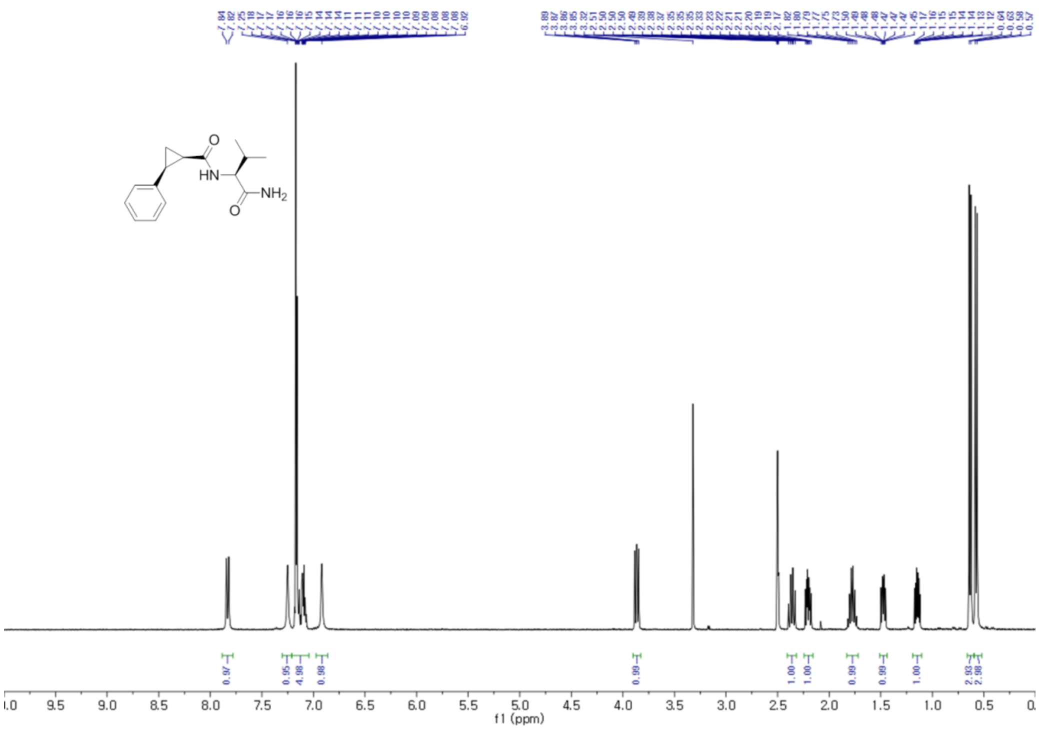

400 MHz, ^{1}H NMR in DMSO-d_6

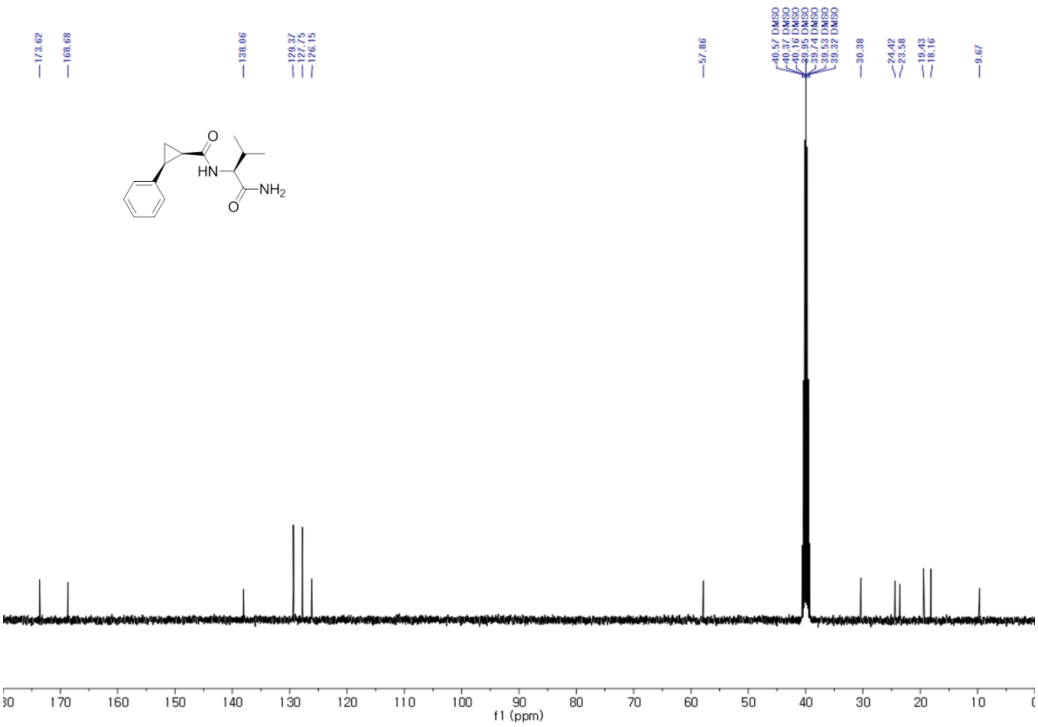

100 MHz, ^{13}C NMR in DMSO-d_6

(1*S*, 2*R*)-*N*-((*S*)-1-amino-3-methyl-1-oxobutan-2-yl)-2-phenylcyclopropanecarboxamide (2b)

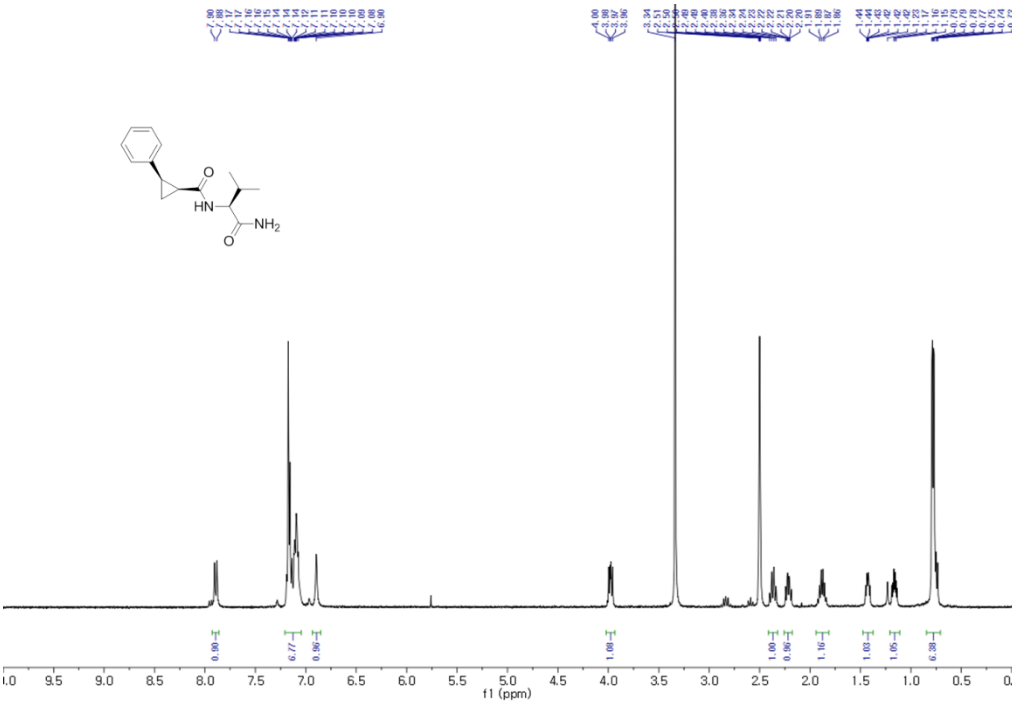

400 MHz, ^{1}H NMR in DMSO-d_6

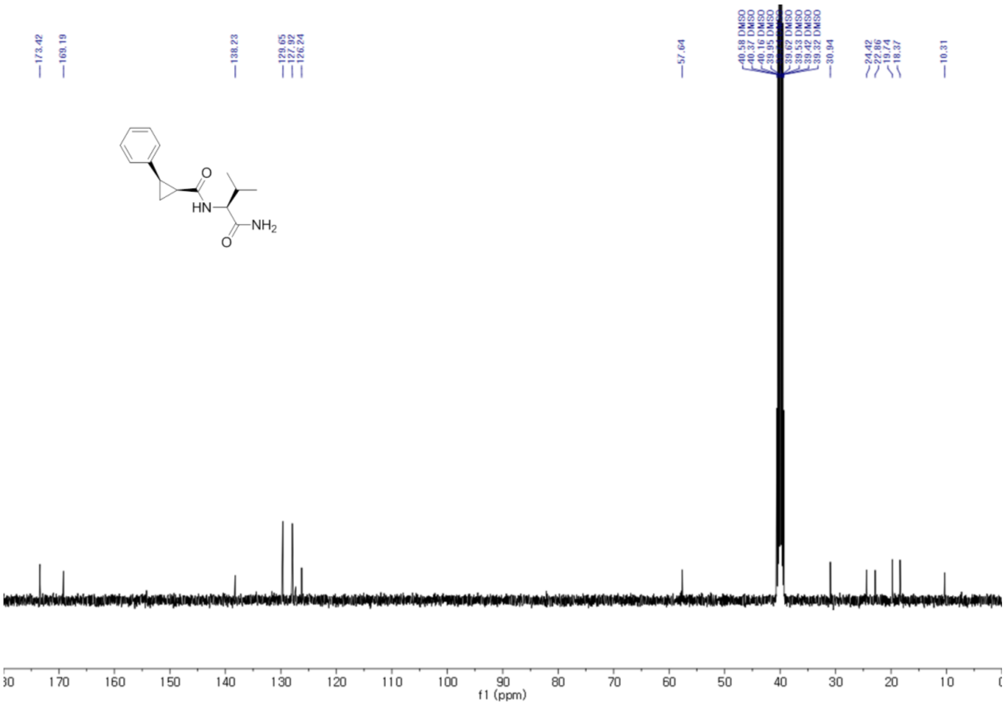

100 MHz, ^{13}C NMR in DMSO-d_6

(1*s*, 2*R*, 3*S*)-*N*-((*S*)-1-amino-3-methyl-1-oxobutan-2-yl)-2,3-diphenylcyclopropanecarboxamide

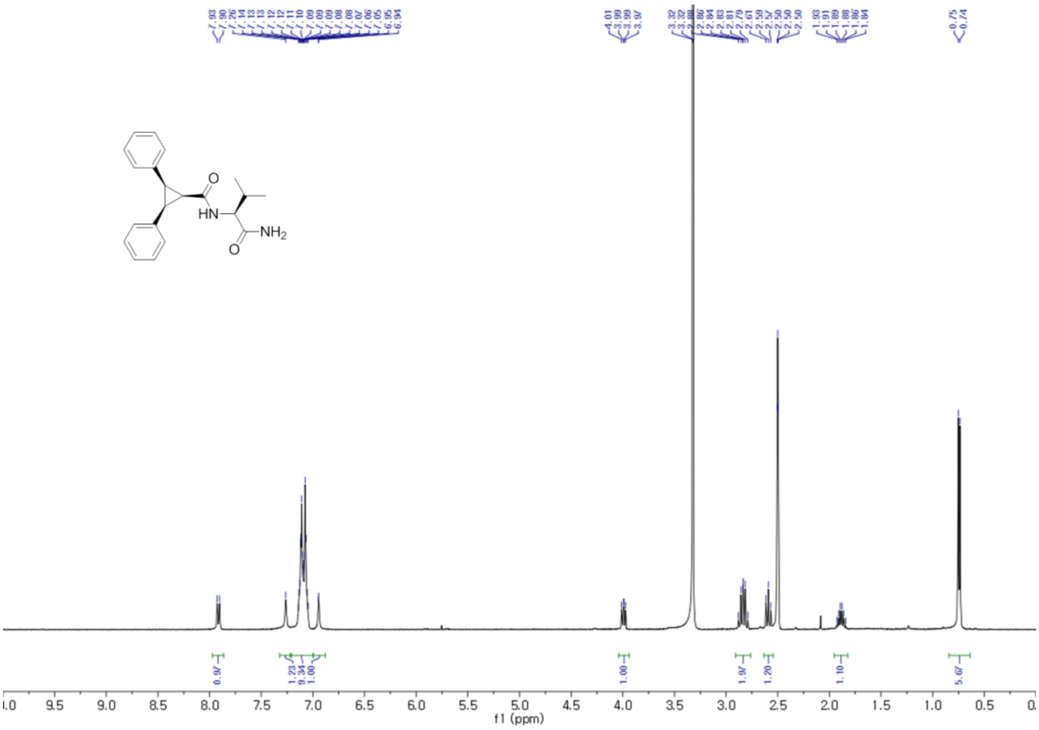

400 MHz, ^{1}H NMR in DMSO-d_6

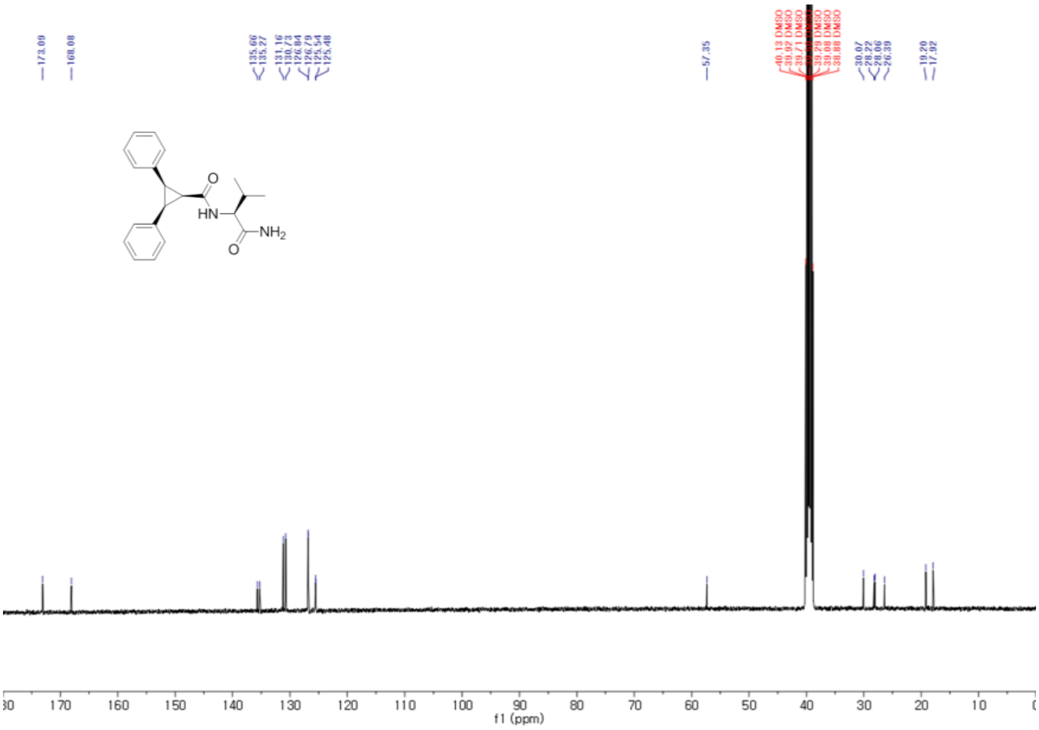

100 MHz, ^{13}C NMR in DMSO-d_6

(1*R*, 2*S*)-*N*-((2*S*, 3*R*)-1-amino-3-methyl-1-oxopentan-2-yl)-2-phenylcyclopropanecarboxamide (3a)

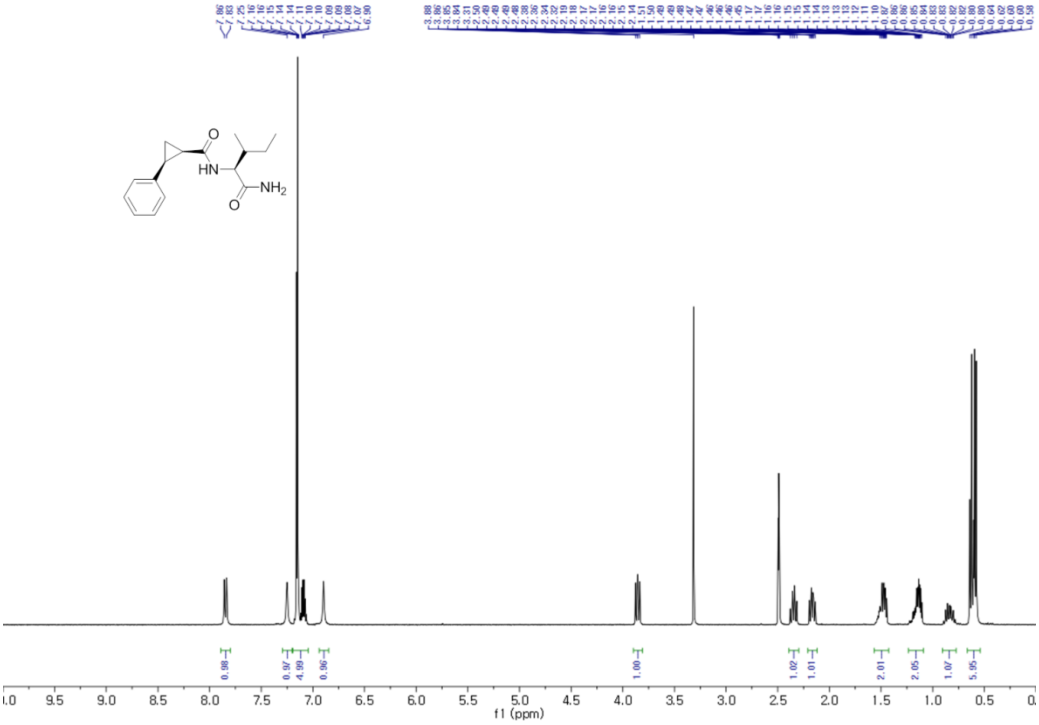

400 MHz, ^{1}H NMR in DMSO-d_6

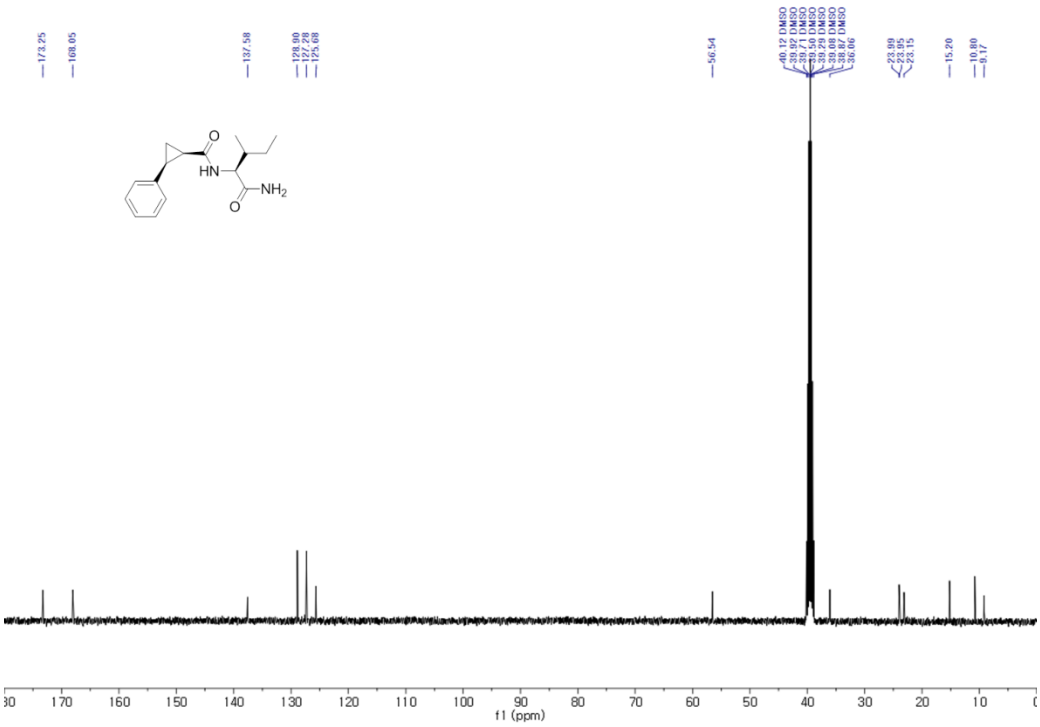

100 MHz, ^{13}C NMR in DMSO-d_6

(1*R*,2*S*)-*N*-((*S*)-1-amino-3,3-dimethyl-1-oxobutan-2-yl)-2-phenylcyclopropanecarboxamide (3b)

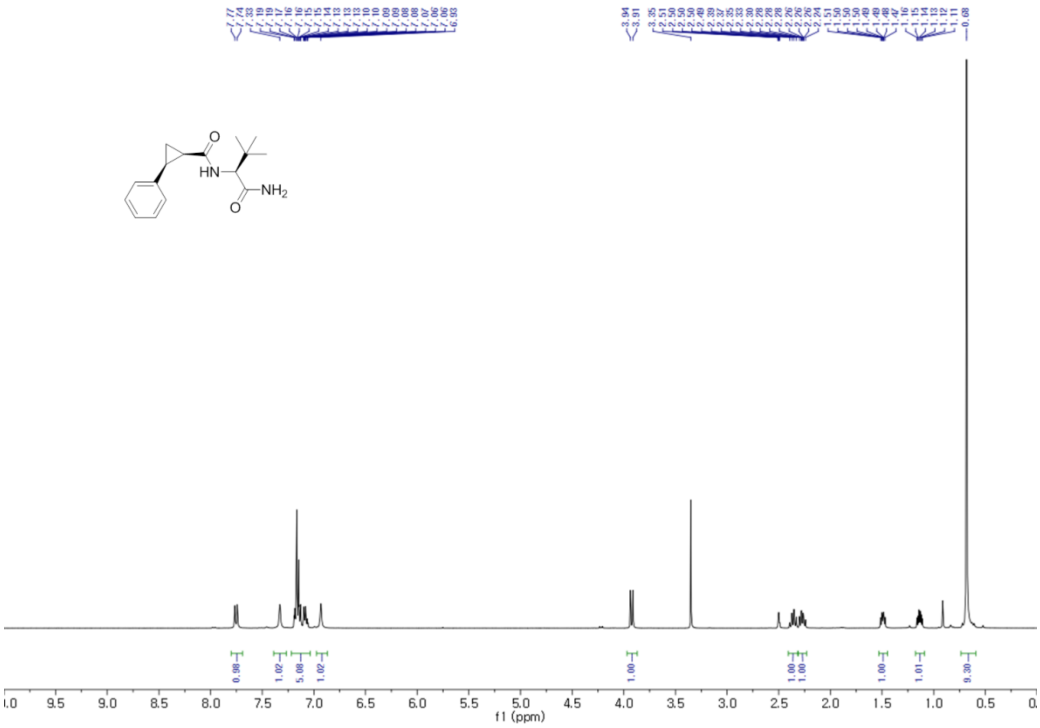

400 MHz, ^{1}H NMR in DMSO-d_6

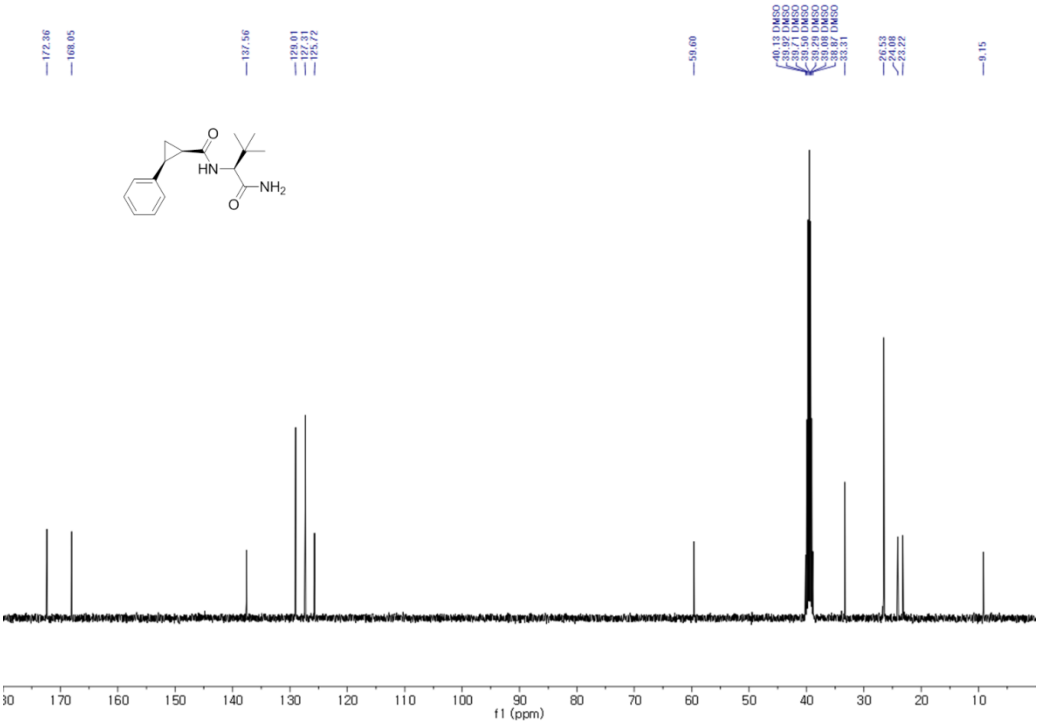

100 MHz, ^{13}C NMR in DMSO-d_6

(1*R*, 2*S*)-*N*-((2*S*, 3*S*)-1-amino-3-(benzyloxy)-1-oxobutan-2-yl)-2-phenylcyclopropanecarboxamide (3c)

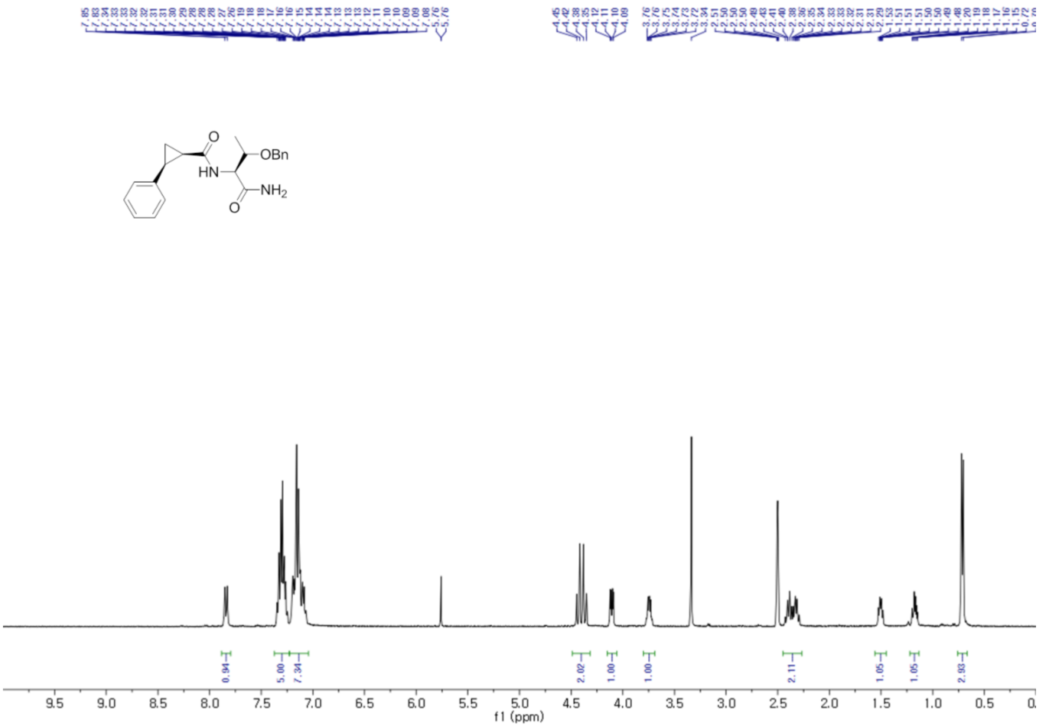

400 MHz, ^{1}H NMR in DMSO-d_6

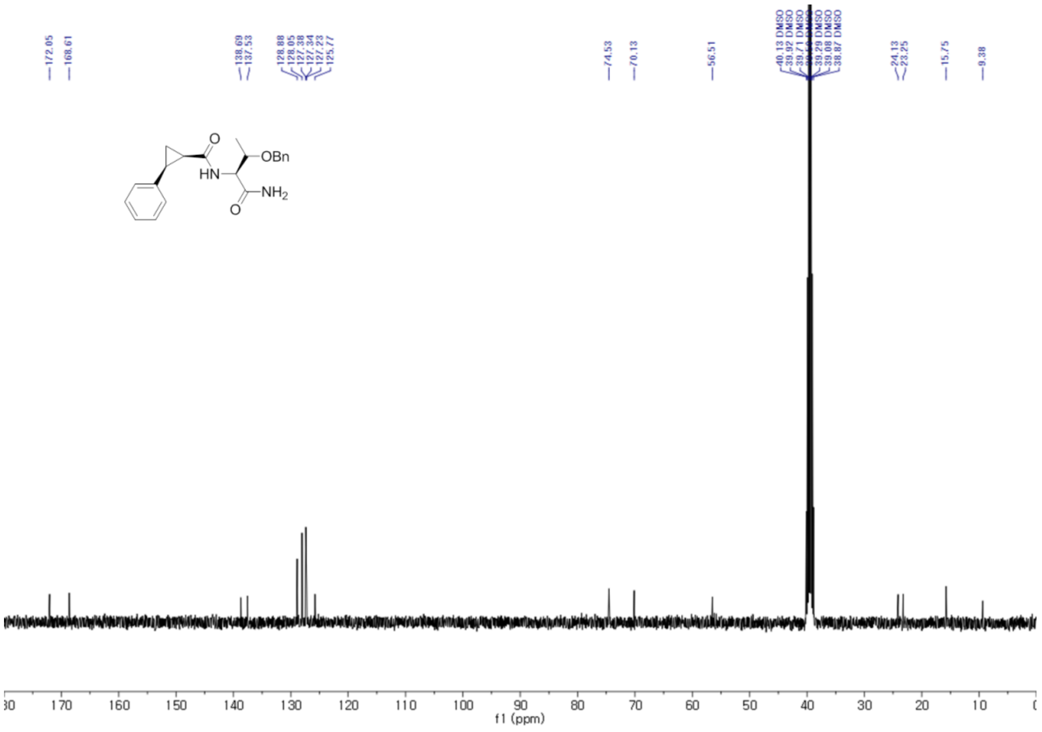

100 MHz, ^{13}C NMR in DMSO-d_6

(1*R*,2*S*)-*N*-((*S*)-2-amino-2-oxo-1-phenylethyl)-2-phenylcyclopropanecarboxamide (3d)

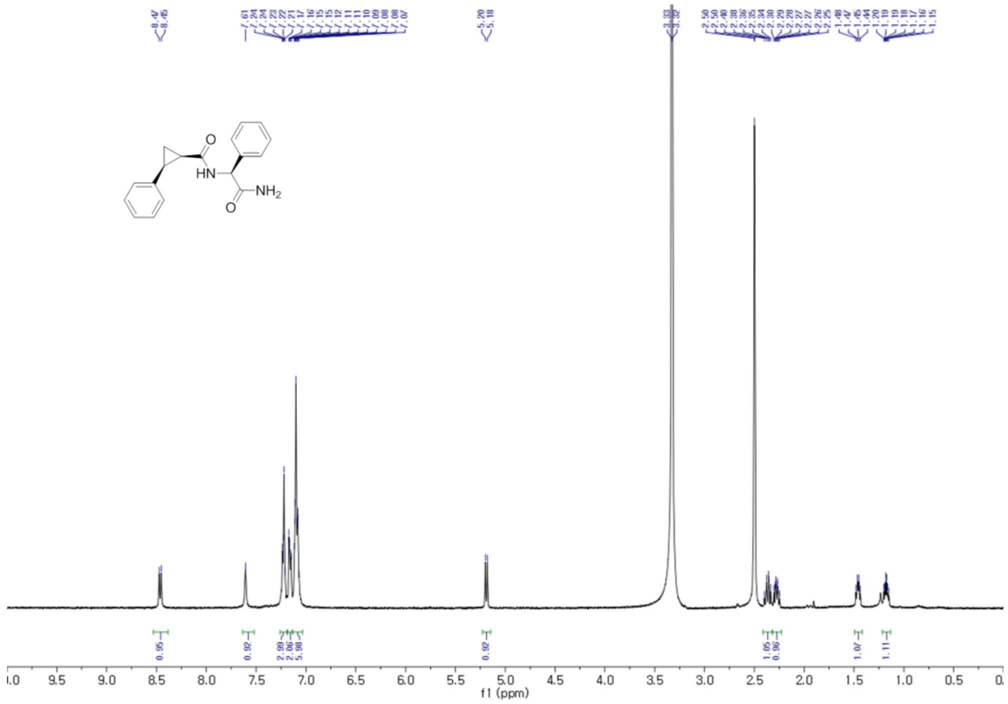

400 MHz, ^{1}H NMR in DMSO-d_6

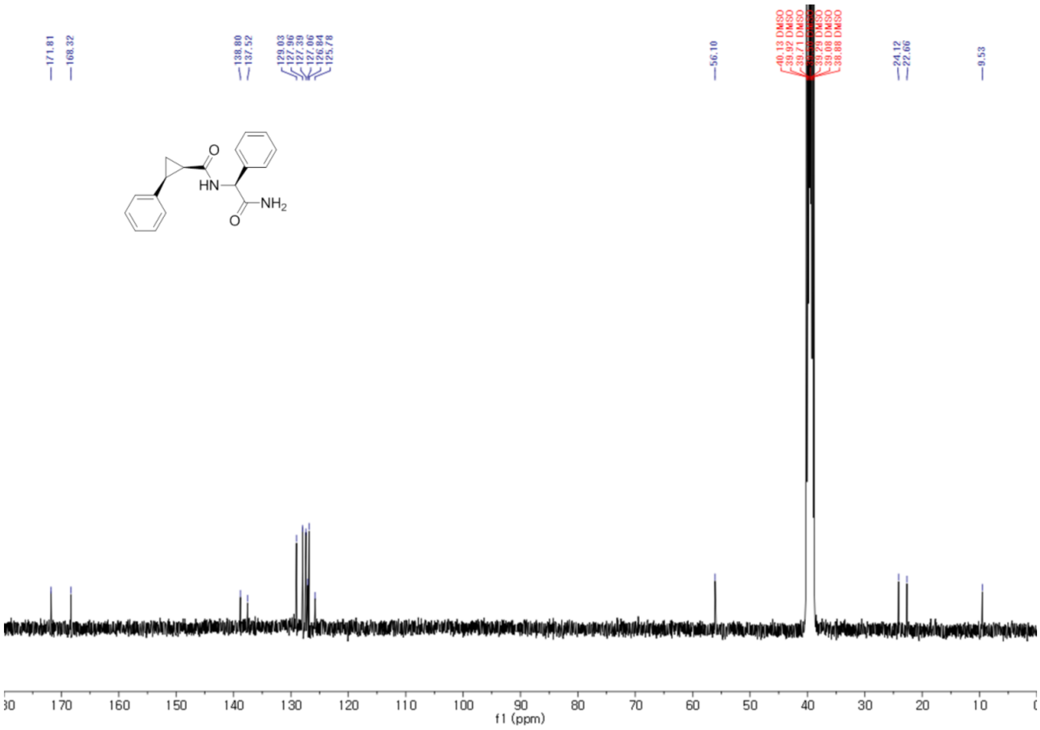

100 MHz, ^{13}C NMR in DMSO-d_6

(1*R*,2*S*)-*N*-((*S*)-2-amino-1-cyclohexyl-2-oxoethyl)-2-phenylcyclopropanecarboxamide (3e)

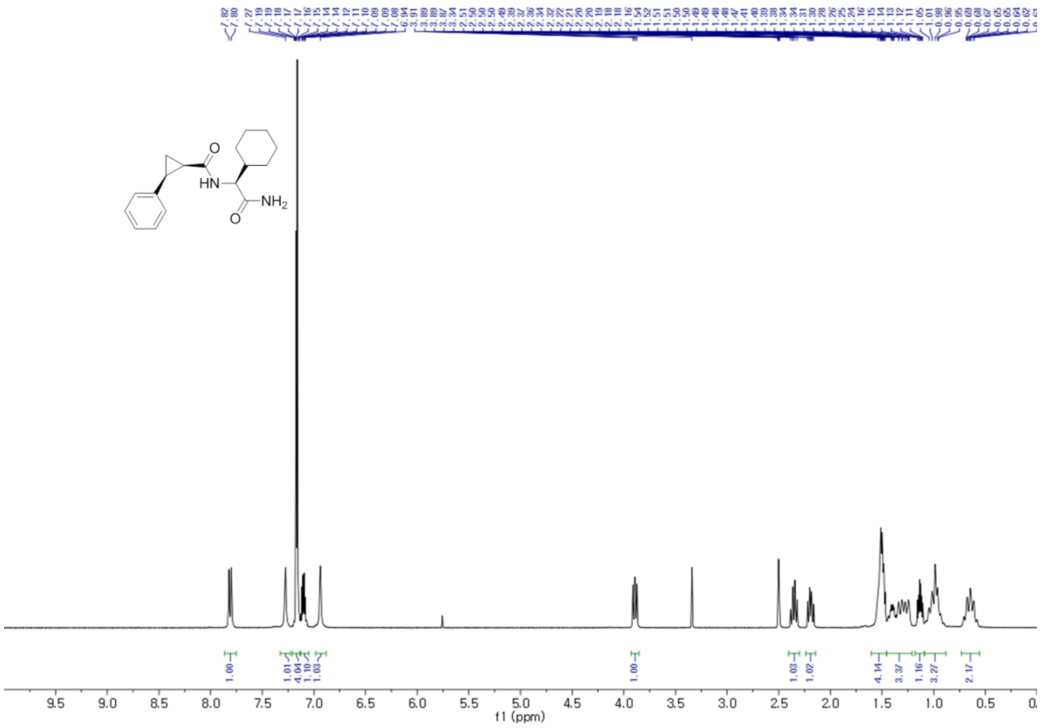

400 MHz, ^{1}H NMR in DMSO-d_6

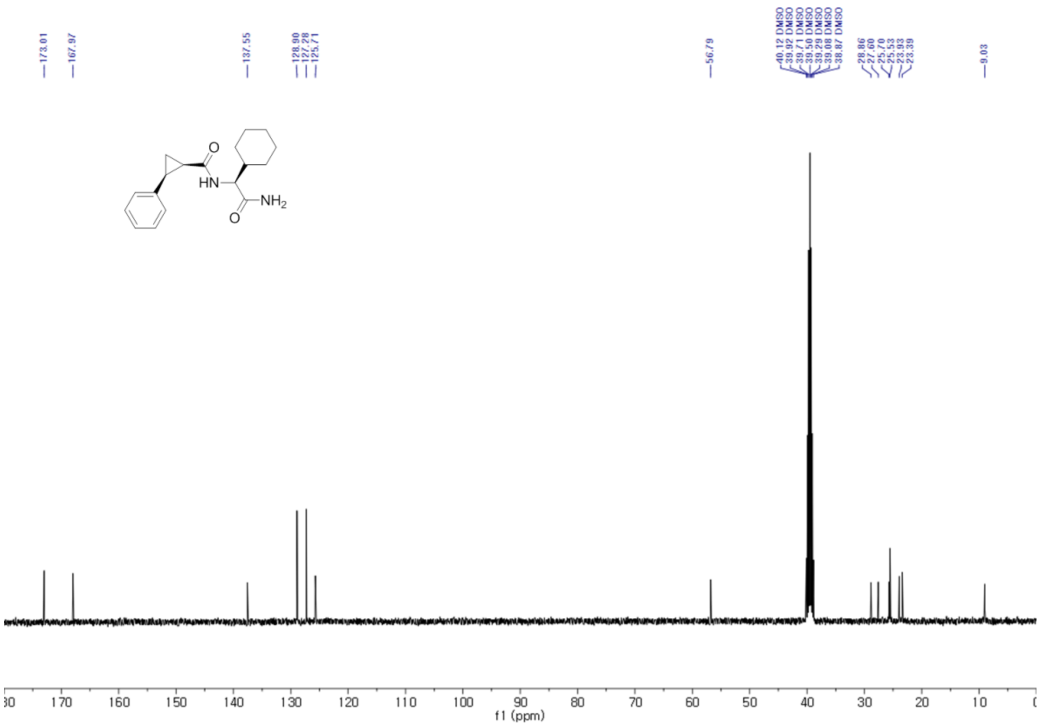

100 MHz, ^{13}C NMR in DMSO-d_6

(1*R*,2*S*)-*N*-((*S*)-1-amino-1-oxo-3-phenylpropan-2-yl)-2-phenylcyclopropanecarboxamide (3f)

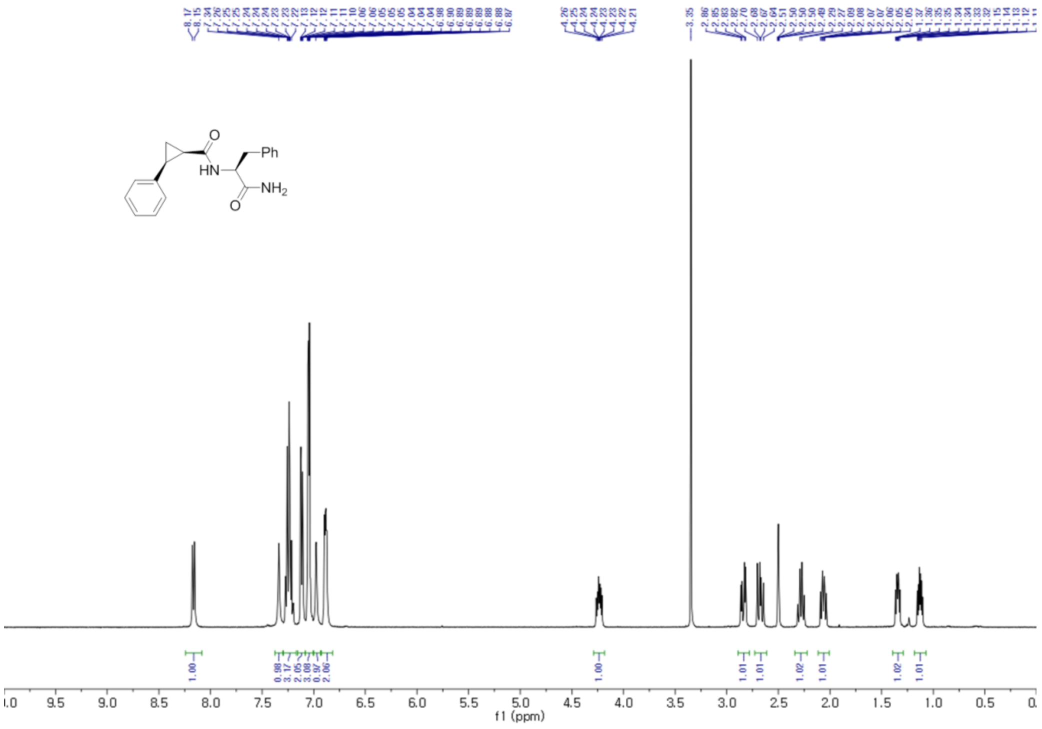

400 MHz, ^{1}H NMR in DMSO-d_6

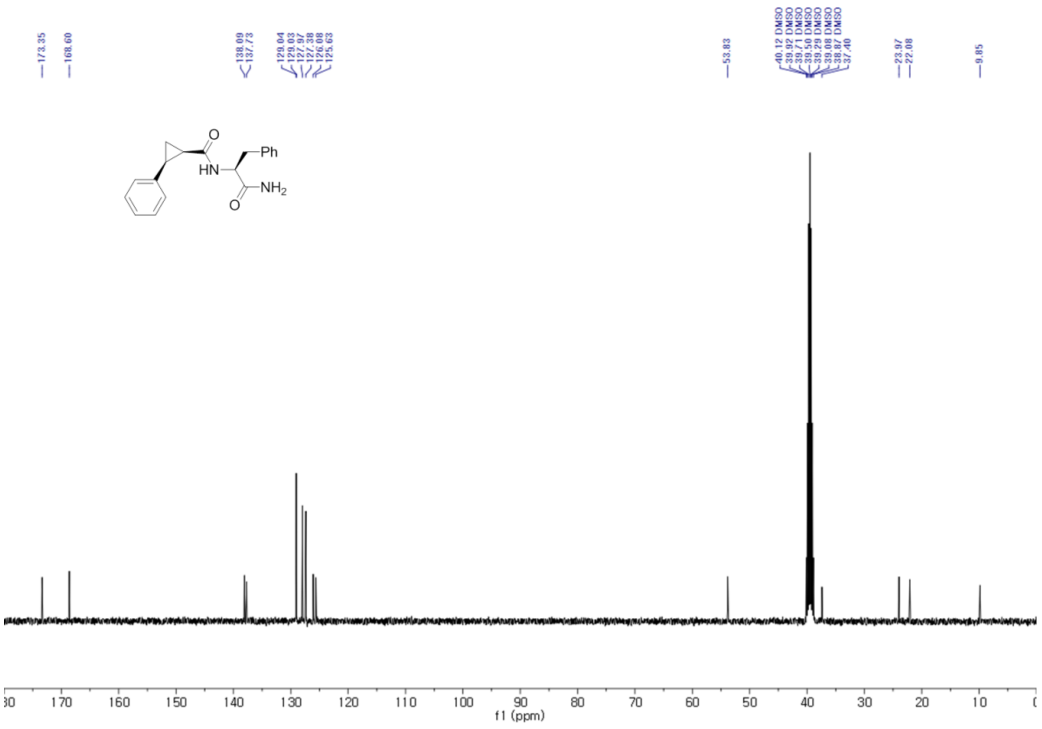

100 MHz, ^{13}C NMR in DMSO-d_6

(1*R*, 2*S*)-*N*-((*S*)-1-amino-3-methyl-1-oxopentan-2-yl)-2-(*p*-tolyl)cyclopropanecarboxamide (3g)

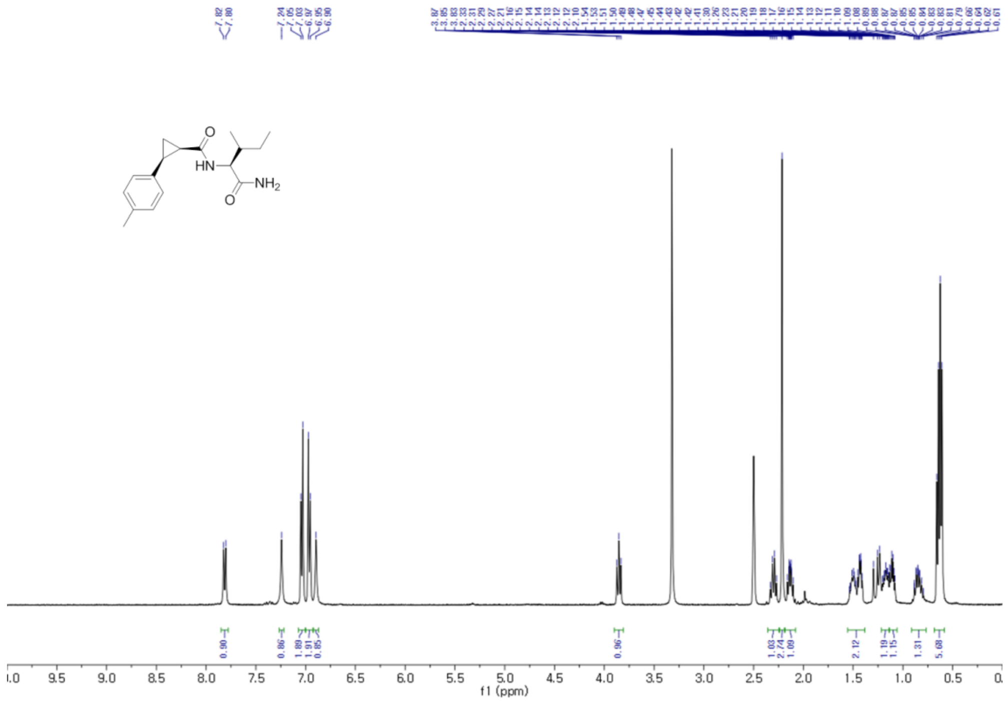

400 MHz, ^{1}H NMR in DMSO-d_6

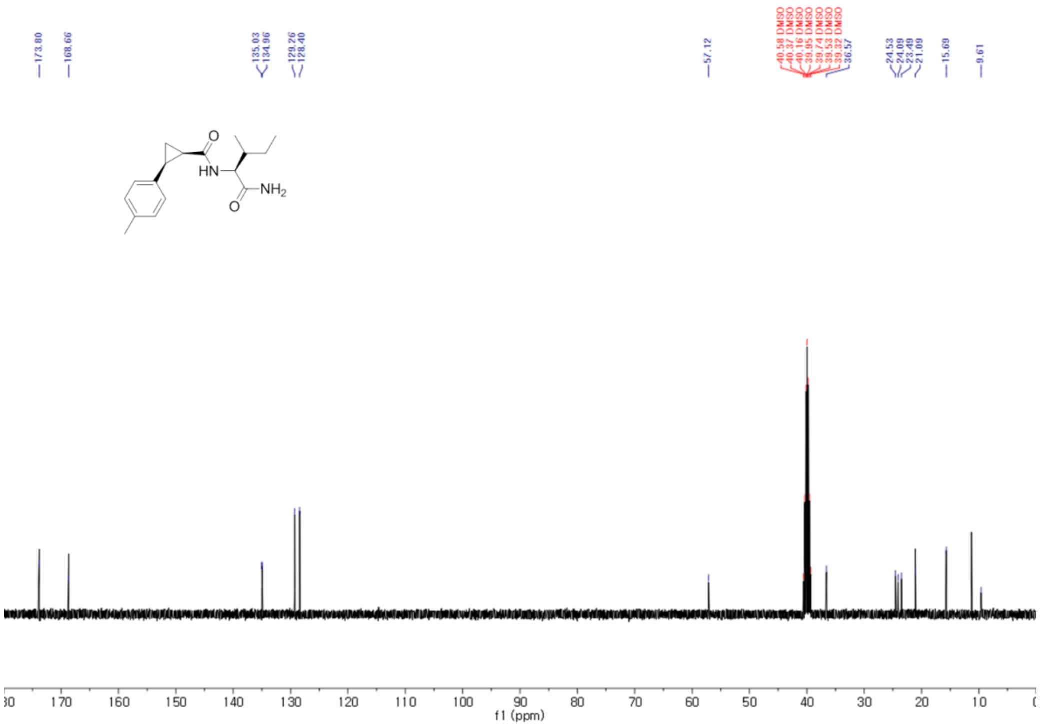

100 MHz, ^{13}C NMR in DMSO-d_6

(1*R*, 2*S*)-*N*-((2*S*, 3*R*)-1-amino-3-methyl-1-oxopentan-2-yl)-2-(4-(*tert*-butyl)phenyl)cyclopropanecarboxamide (3h)

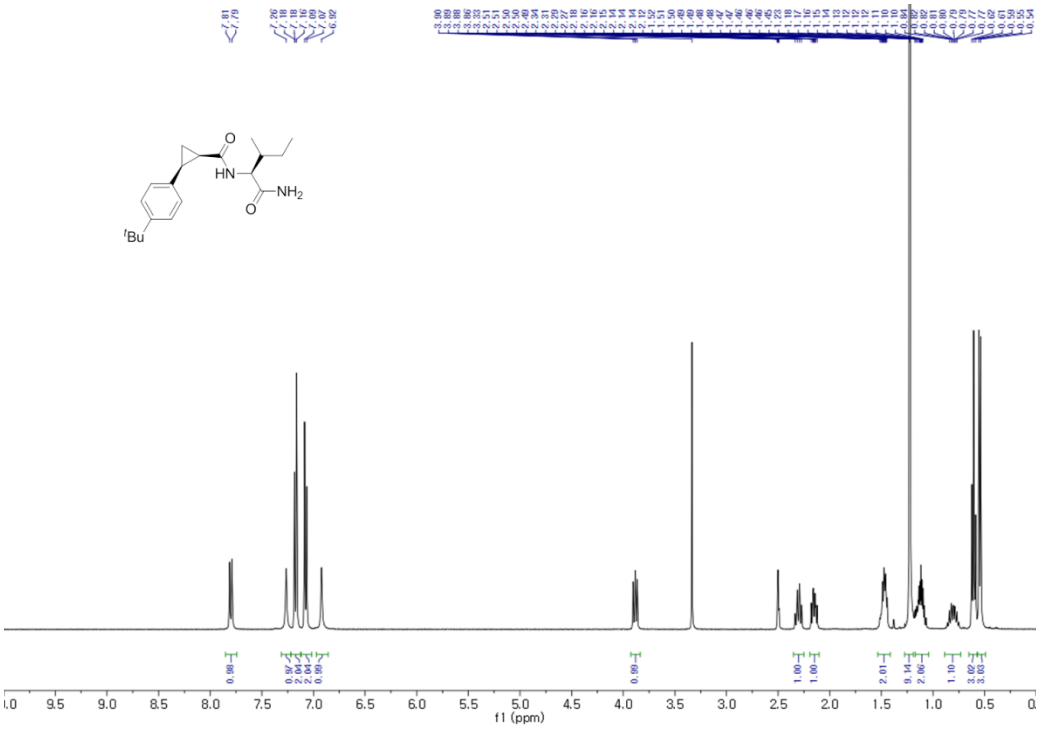

400 MHz, ^{1}H NMR in DMSO-d_6

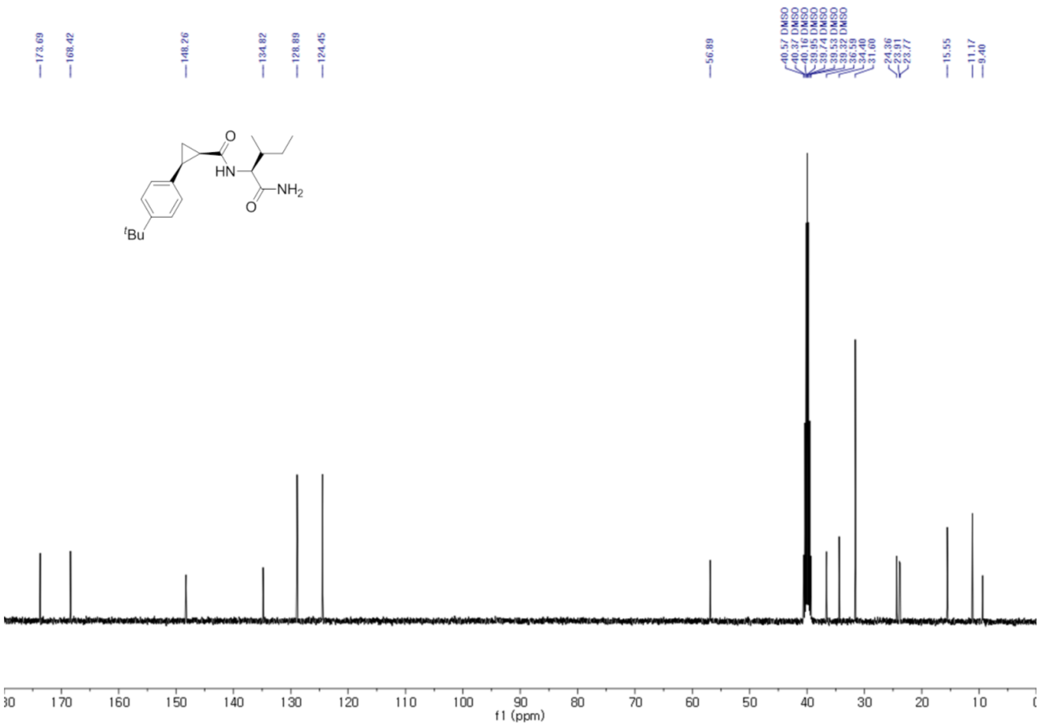

100 MHz, ^{13}C NMR in DMSO-d_6

(1*R*, 2*S*)-*N*-((*S*)-1-amino-3-methyl-1-oxopentan-2-yl) -2 -([1,1'-biphenyl]-4-yl)cyclopropanecarboxamide (3i)

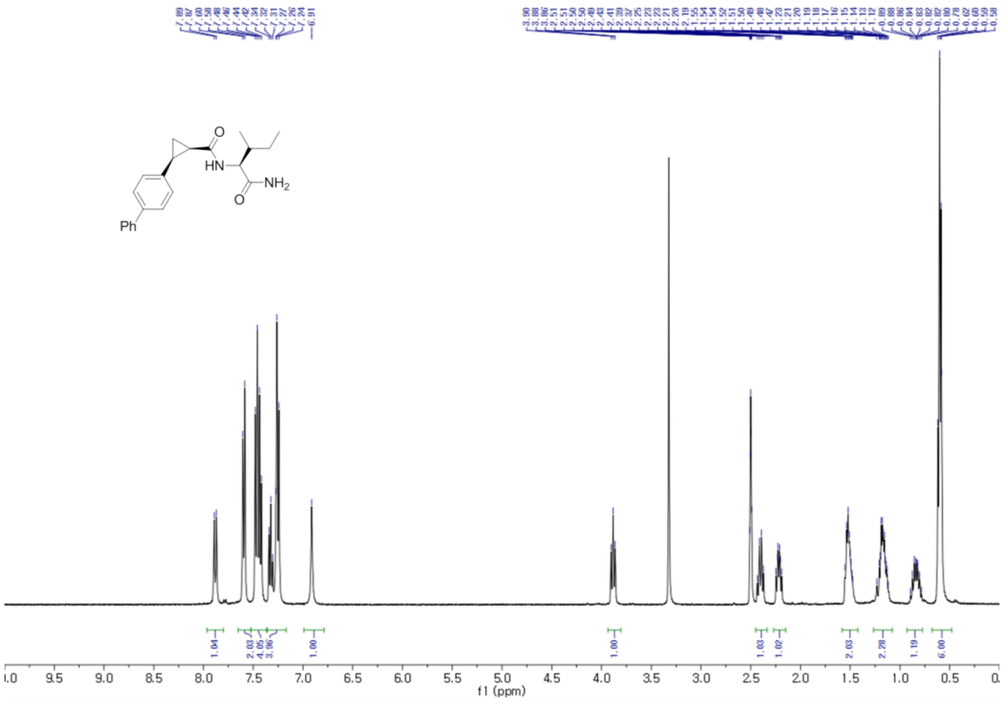

400 MHz, ^{1}H NMR in DMSO-d_6

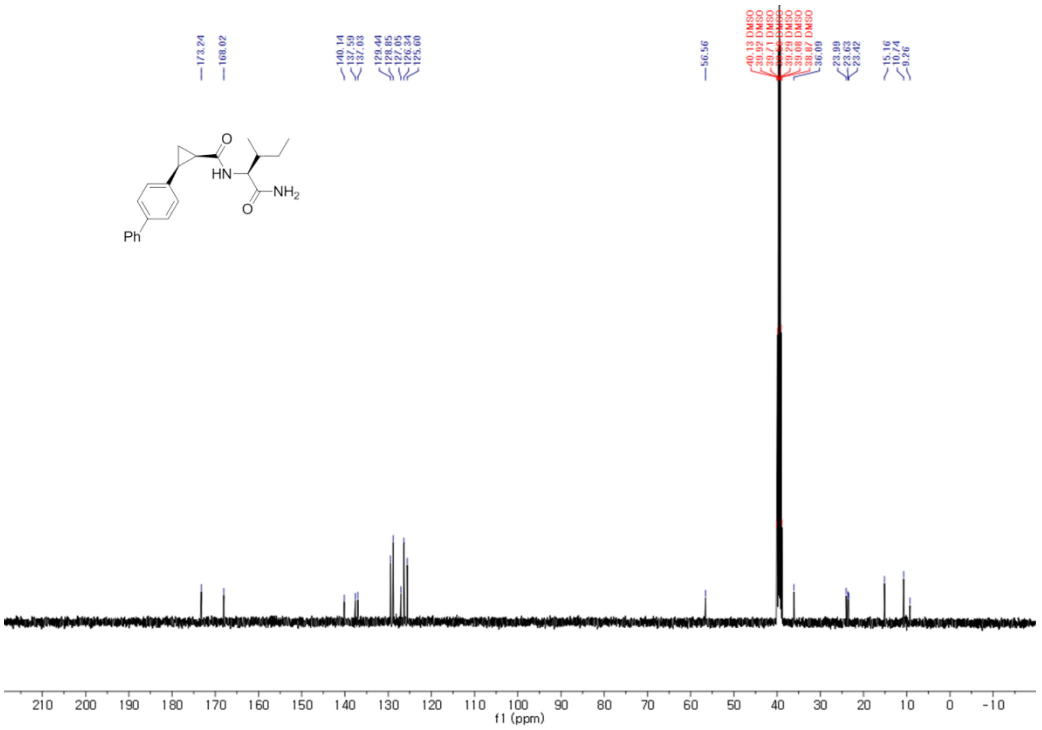

100 MHz, ^{13}C NMR in DMSO-d_6

(1*R*, 2*S*)-*N*-((*S*)-1-amino-3-methyl-1-oxopentan-2-yl)-2-(4-methoxyphenyl)cyclopropanecarboxamide (3j)

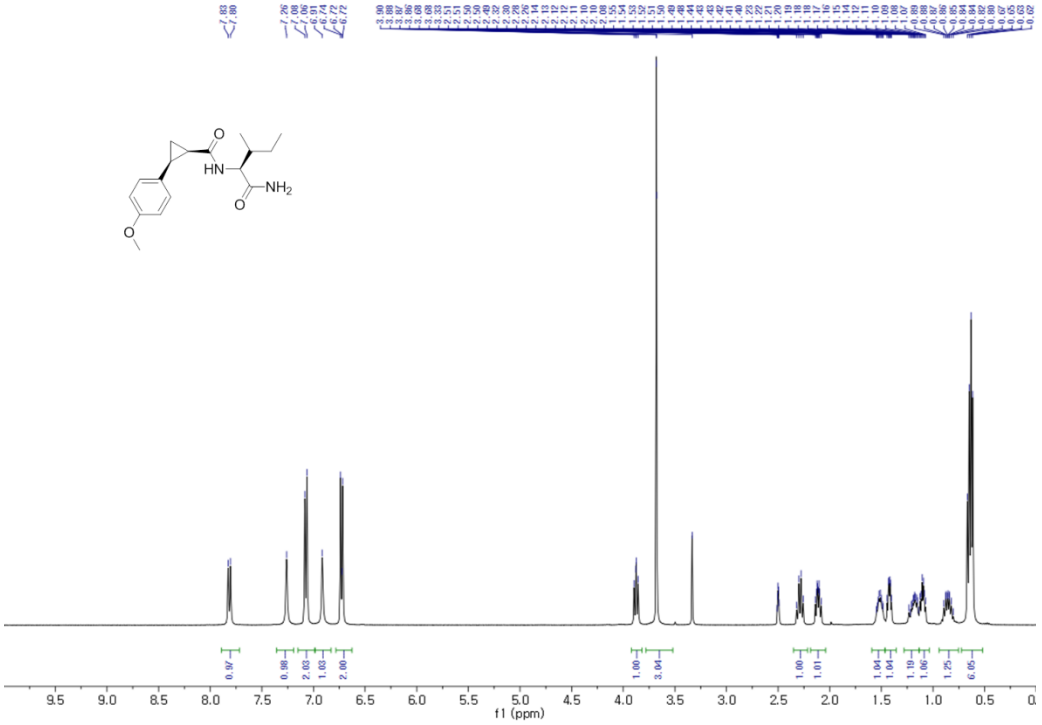

400 MHz, ^{1}H NMR in DMSO-d_6

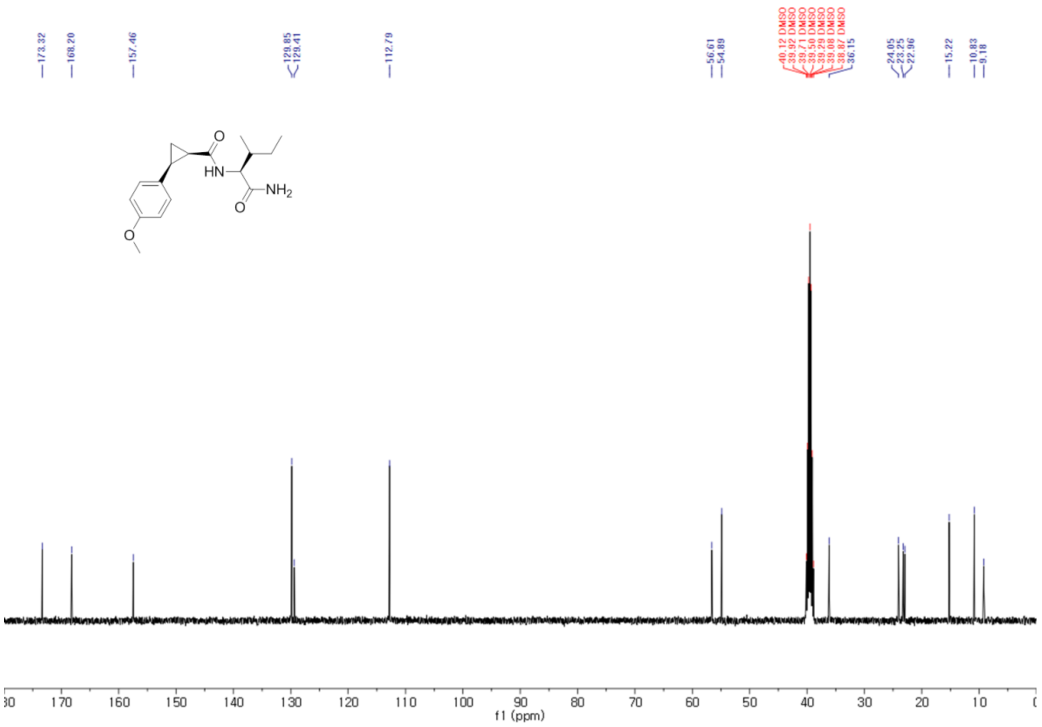

100 MHz, ^{13}C NMR in DMSO-d_6

(1*R*, 2*S*)-*N*-((2*S*, 3*R*)-1-amino-3-methyl-1-oxopentan-2-yl)-2-(3,5-dimethylphenyl)cyclopropanecarboxamide (3k)

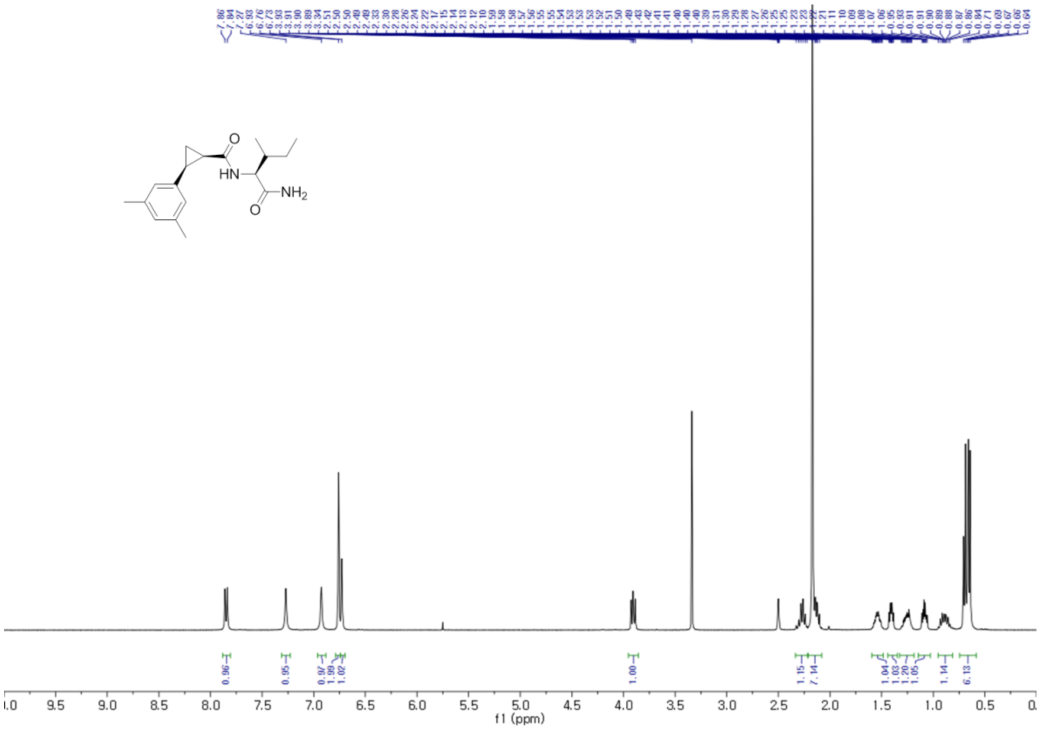

400 MHz, ^{1}H NMR in DMSO-d_6

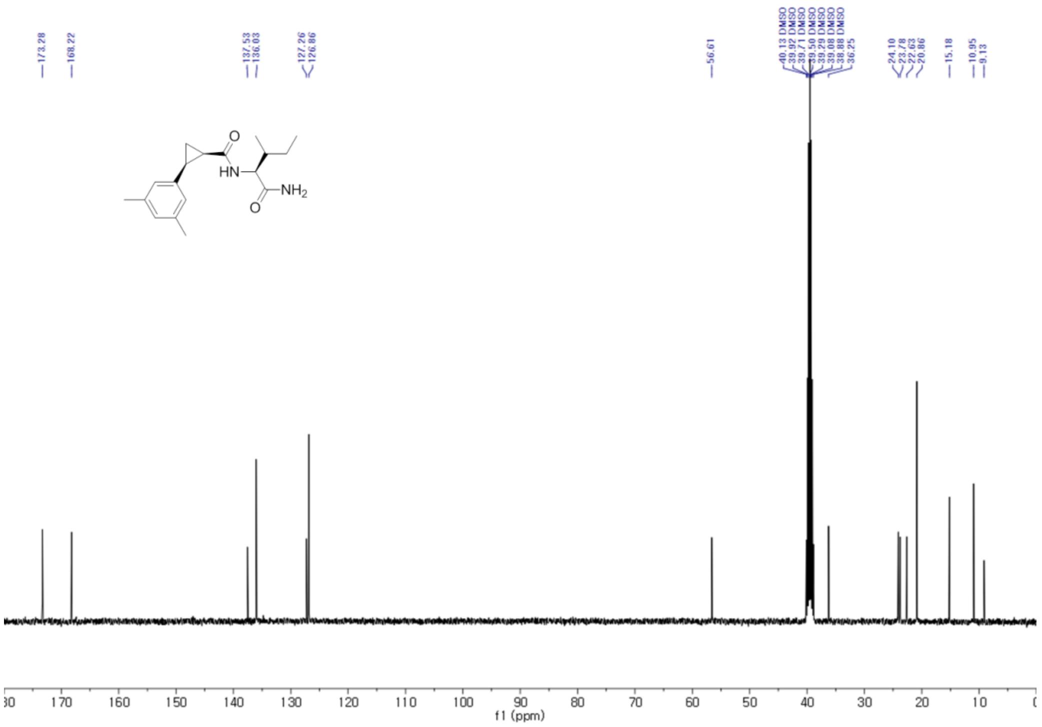

100 MHz, ^{13}C NMR in DMSO-d_6

(1*R*, 2*S*)-*N*-((2*S*, 3*R*)-1-amino-3-methyl-1-oxopentan-2-yl)-2-(3,5-dichlorophenyl)cyclopropanecarboxamide (3l)

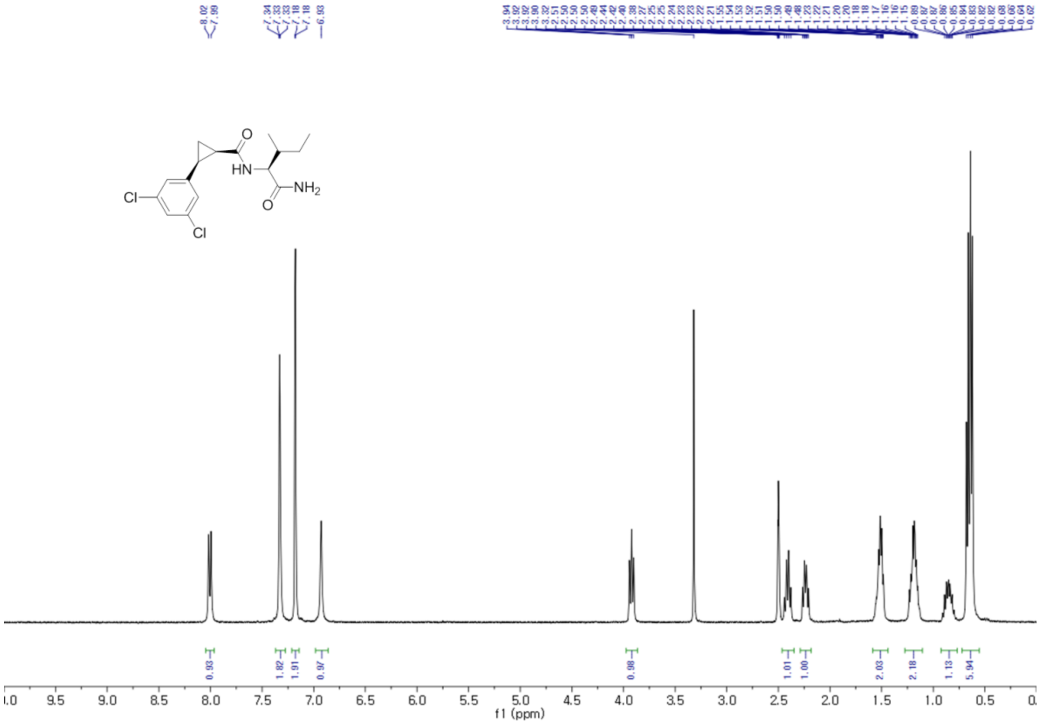

400 MHz, ^{1}H NMR in DMSO-d_6

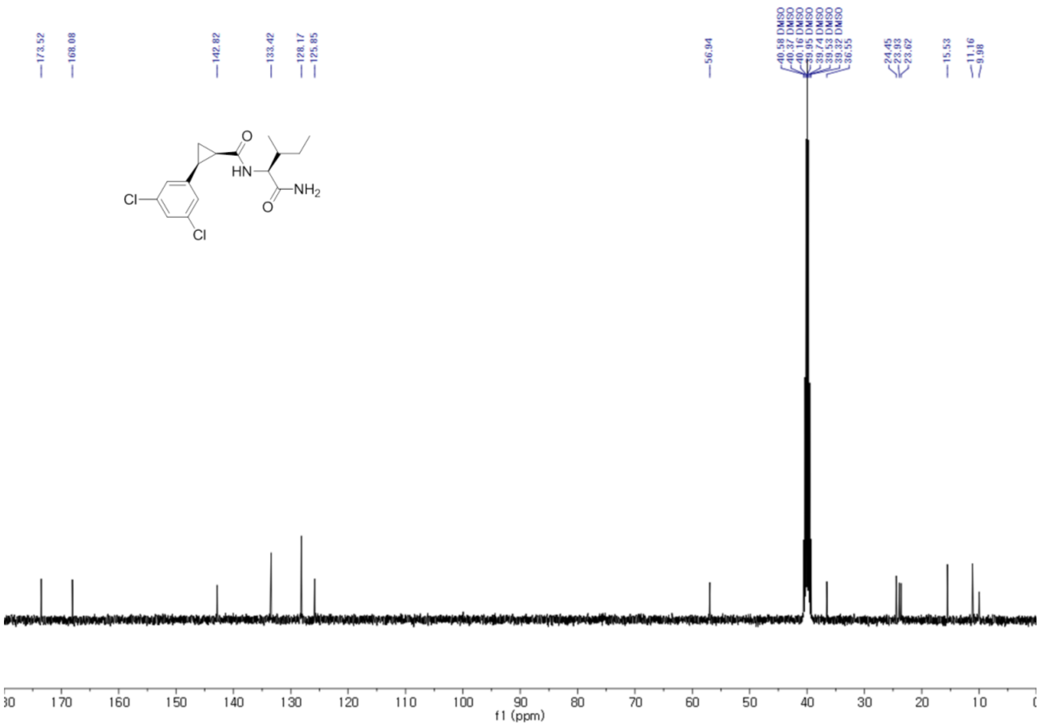

100 MHz, ^{13}C NMR in DMSO-d_6

(1*R*, 2*S*)-*N*-((2*S*, 3*R*)-1-amino-3-methyl-1-oxopentan-2-yl)-2-(3,5-di-*tert*-butylphenyl)cyclopropanecarboxamide (3m)

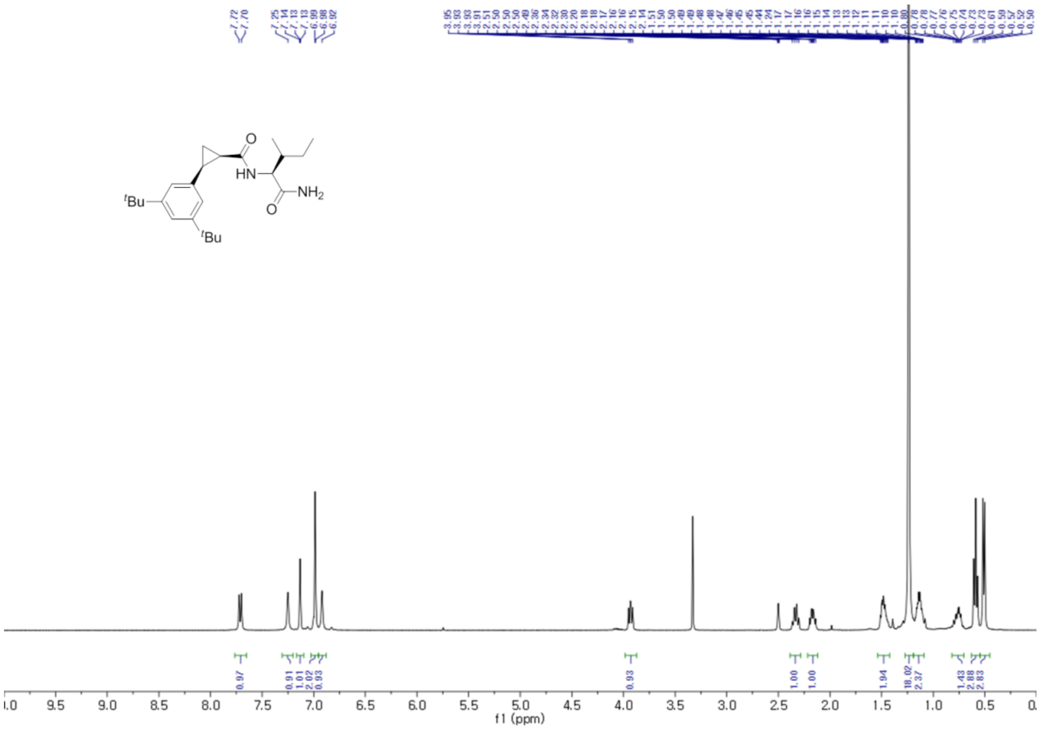

400 MHz, ^{1}H NMR in DMSO-d_6

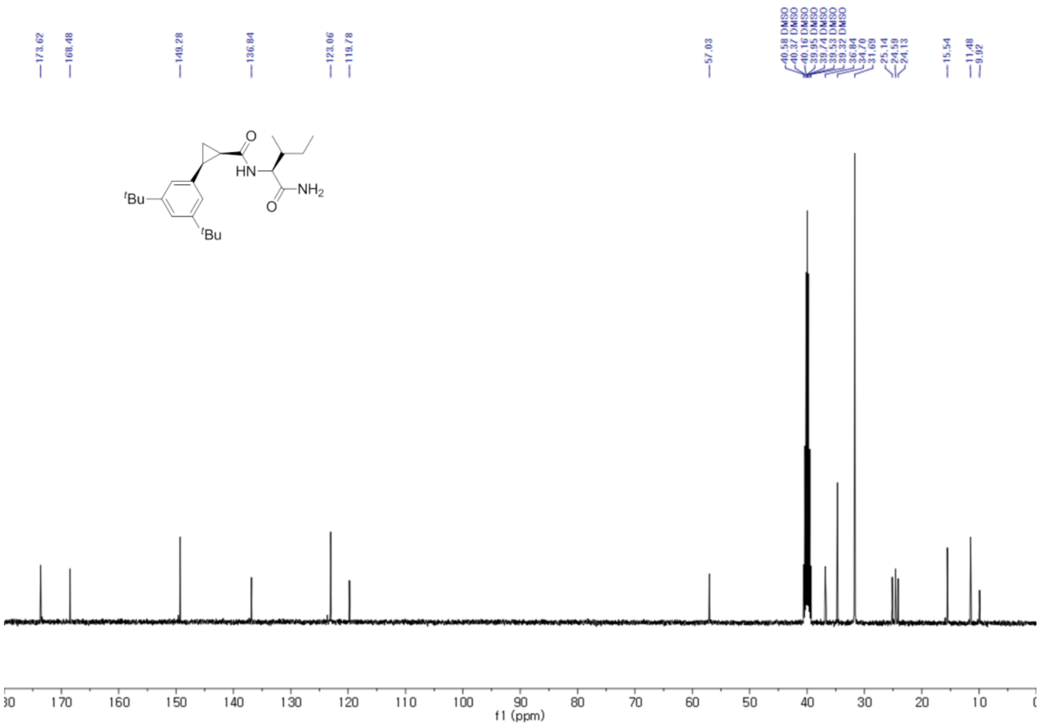

100 MHz, ^{13}C NMR in DMSO-d_6

(1*R*,2*S*)-*N*-((*S*)-1-amino-3-methyl-1-oxopentan-2-yl)-2-(4-bromophenyl)cyclopropanecarboxamide (3n)

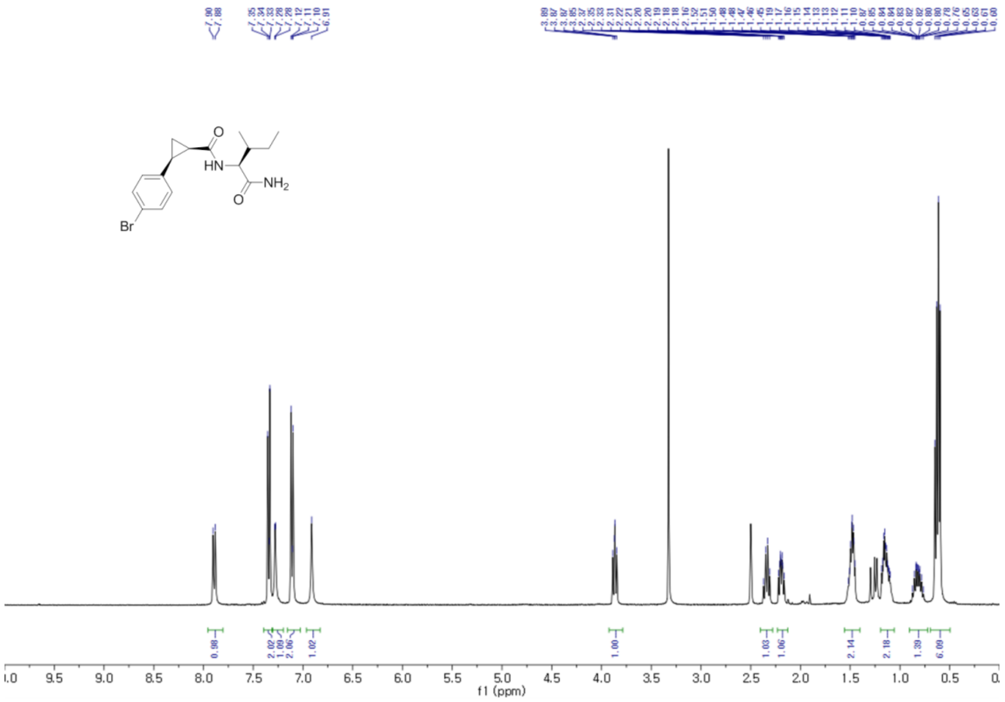

400 MHz, ^{1}H NMR in DMSO-d_6

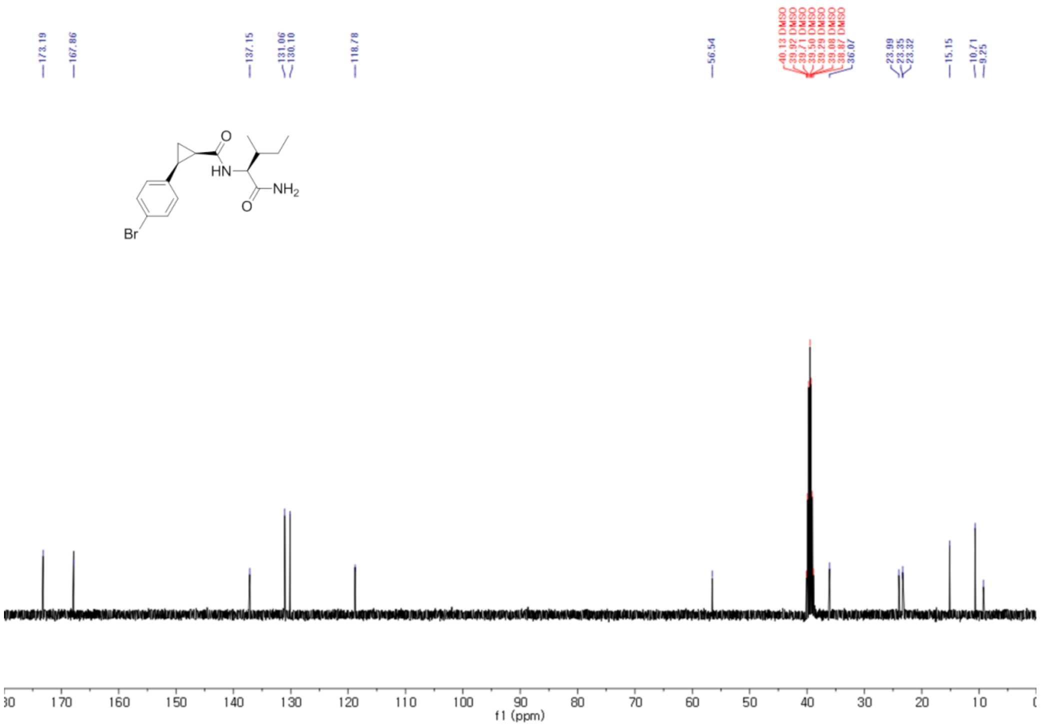

100 MHz, ^{13}C NMR in DMSO-d_6

(1*R*, 2*S*)-*N*-((2*S*, 3*R*)-1-amino-3-methyl-1-oxopentan-2-yl)-2-(4-chlorophenyl)cyclopropanecarboxamide (3o)

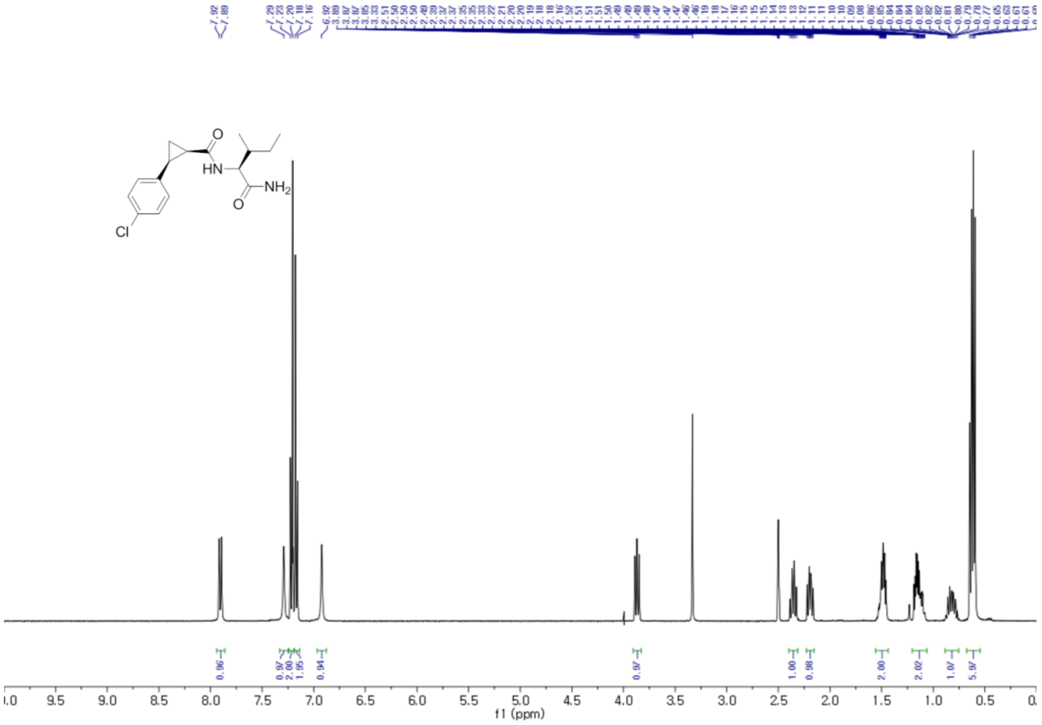

400 MHz, ^{1}H NMR in DMSO-d_6

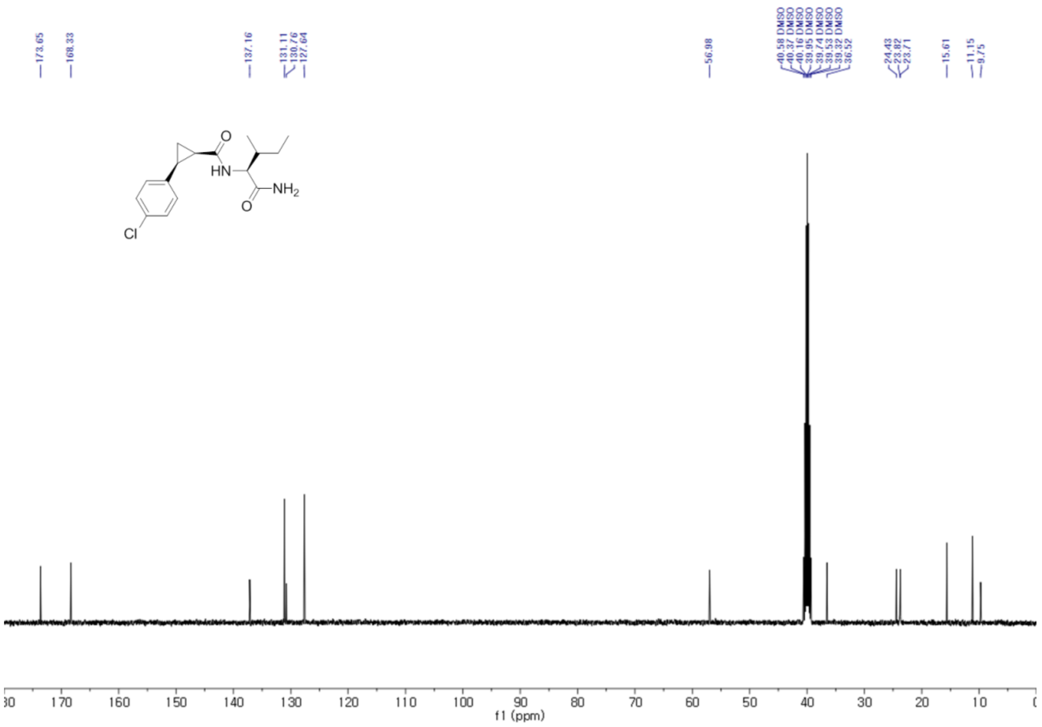

100 MHz, ^{13}C NMR in DMSO-d_6

(1*R*, 2*S*)-N-((2*S*, 3*R*)-1-amino-3-methyl-1-oxopentan-2-yl)-2-(4-fluorophenyl)cyclopropanecarboxamide (3p)

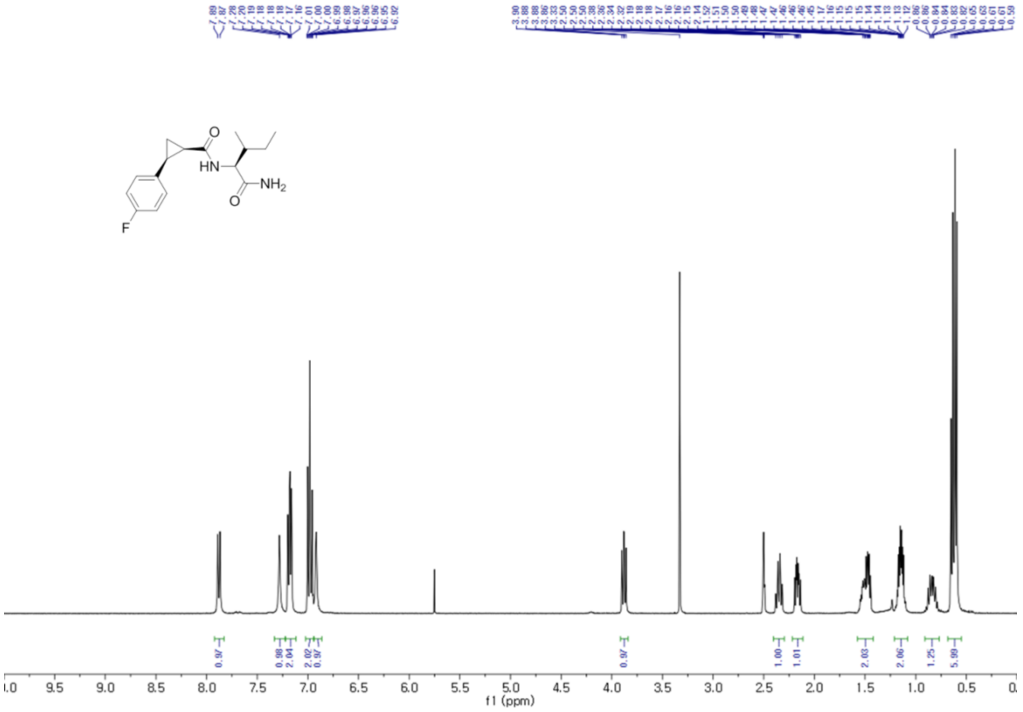

400 MHz, ^{1}H NMR in DMSO-d_6

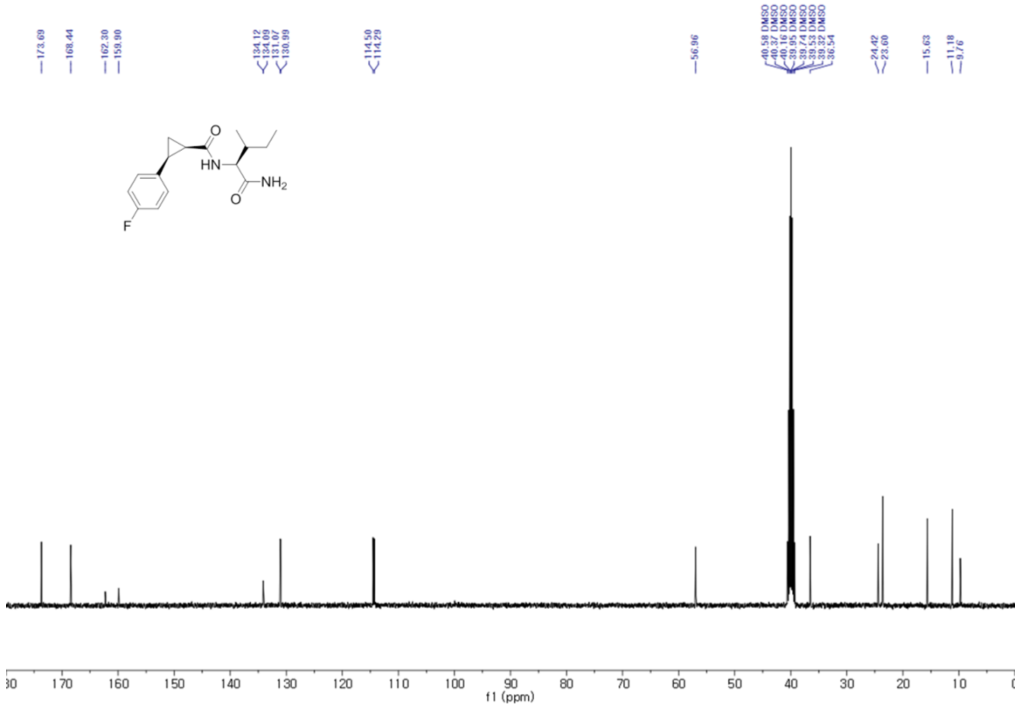

100 MHz, ^{13}C NMR in DMSO-d_6

(1*R*, 2*S*)-*N*-((2*S*, 3*R*)-1-amino-3-methyl-1-oxopentan-2-yl)-2-(4-(trifluoromethyl)phenyl)cyclopropanecarboxamide (3q)

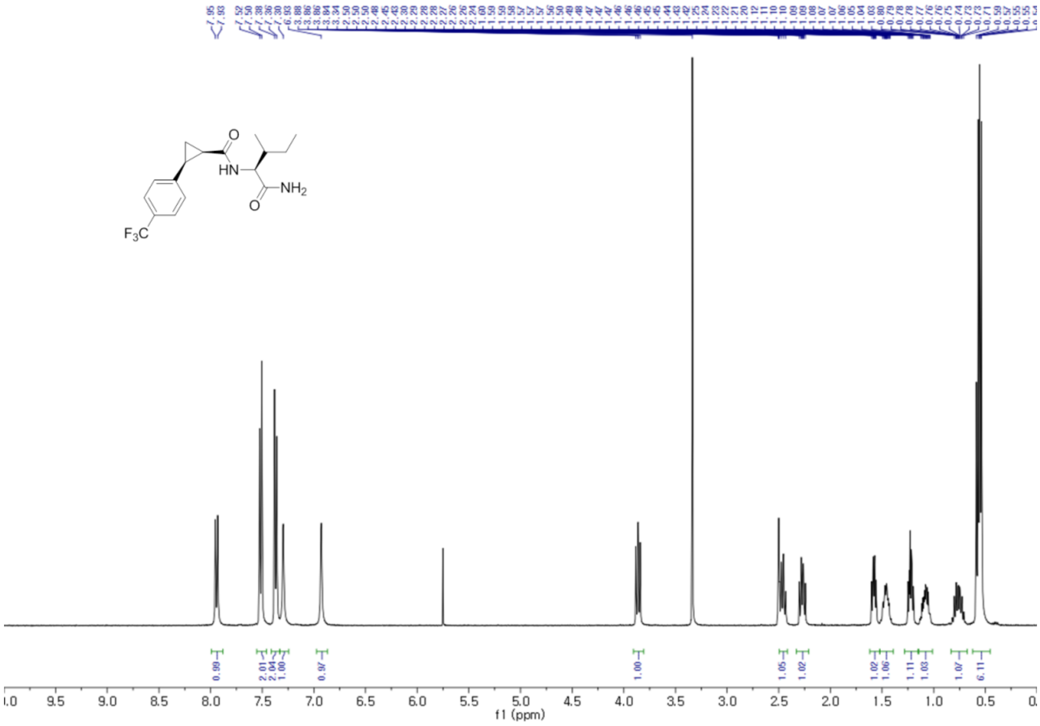

400 MHz, ^{1}H NMR in DMSO-d_6

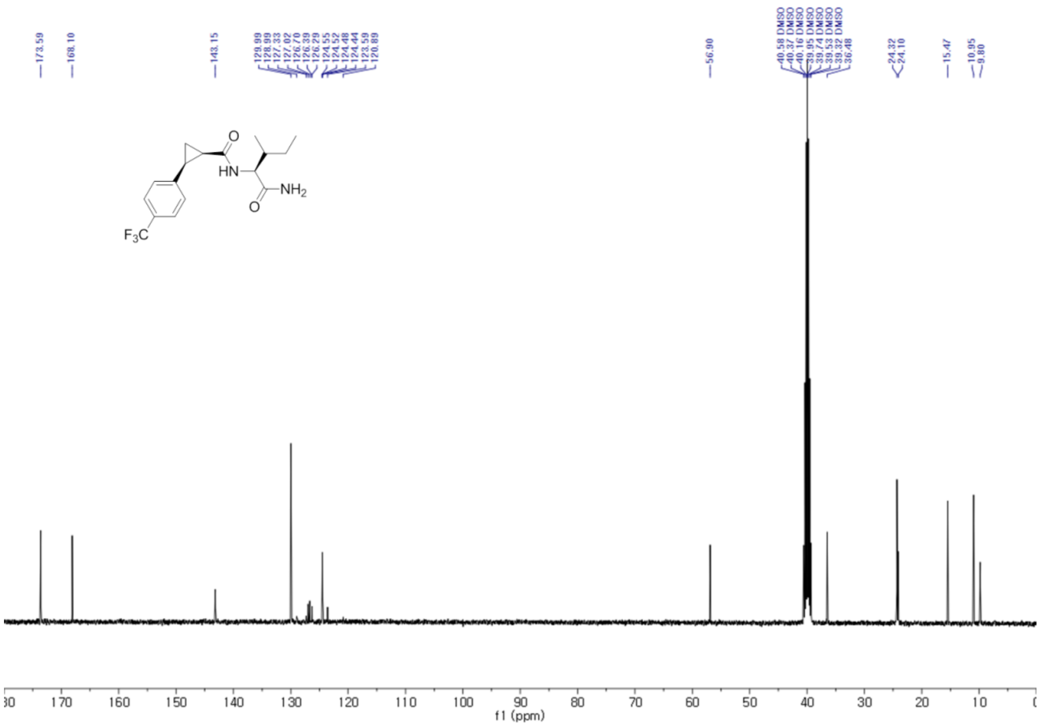

100 MHz, ^{13}C NMR in DMSO-d_6

methyl 4-((1*S*, 2*R*)-2-(((2*S*, 3*R*)-1-amino-3-methyl-1-oxopentan-2-yl)carbamoyl)cyclopropyl)benzoate (3r)

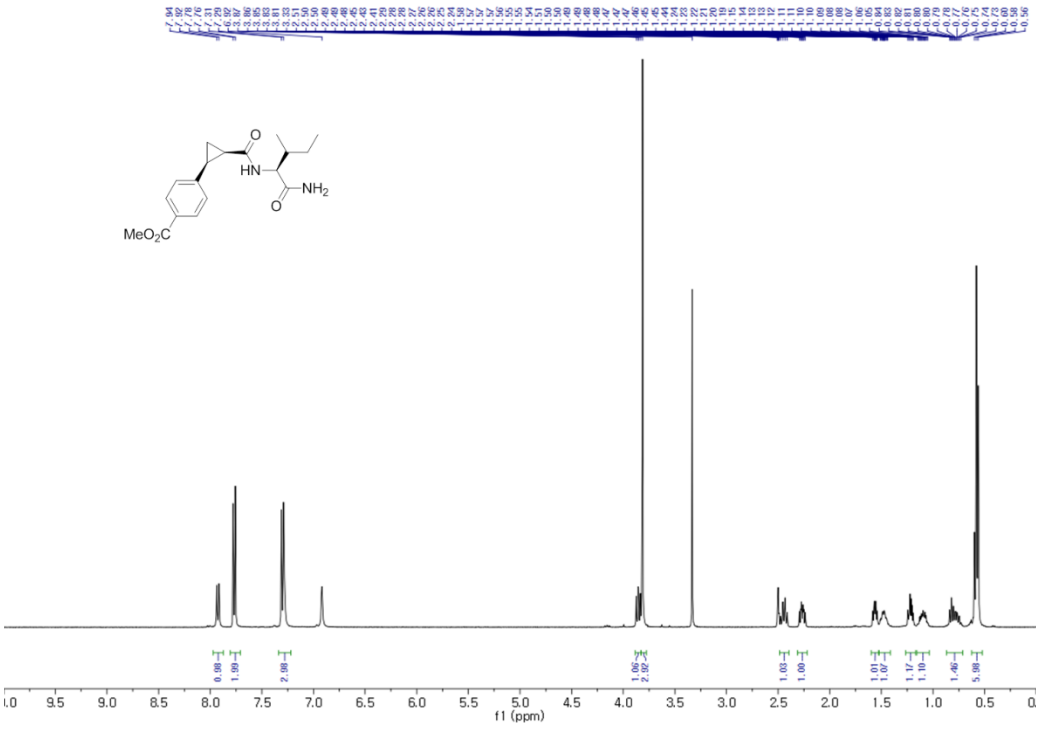

400 MHz, ^{1}H NMR in DMSO-d_6

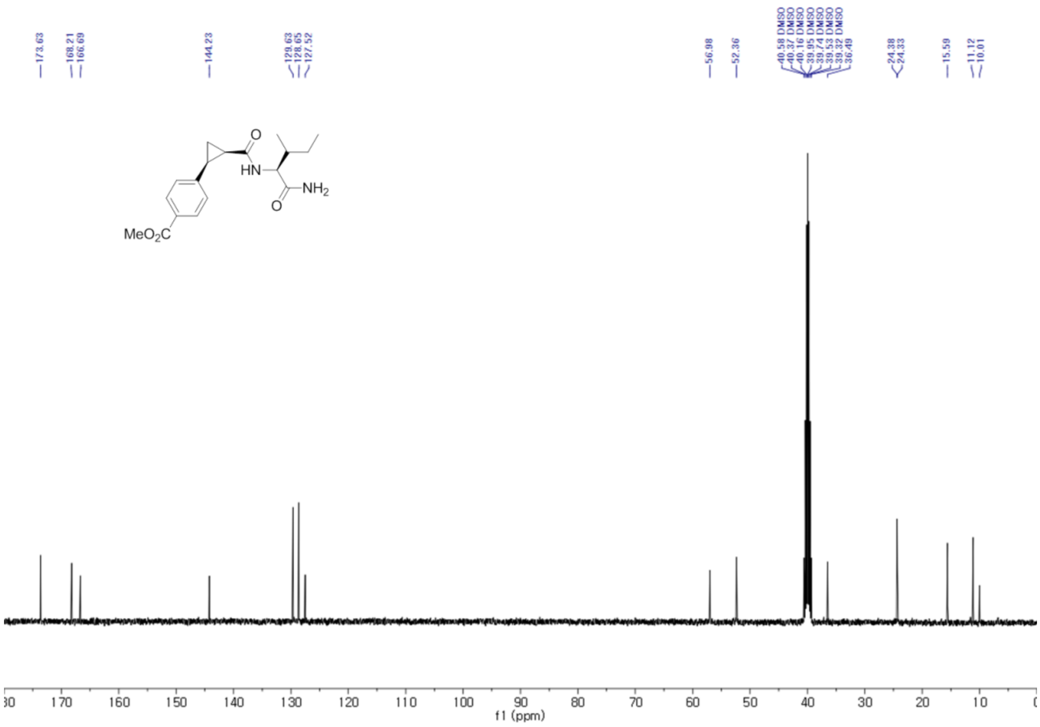

100 MHz, ^{13}C NMR in DMSO-d_6

***cis*-2-(4-acetylphenyl)-*N*-((2*S*, 3*R*)-1-amino-3-methyl-1-oxopentan-2-yl)cyclopropanecarboxamide (3s)**

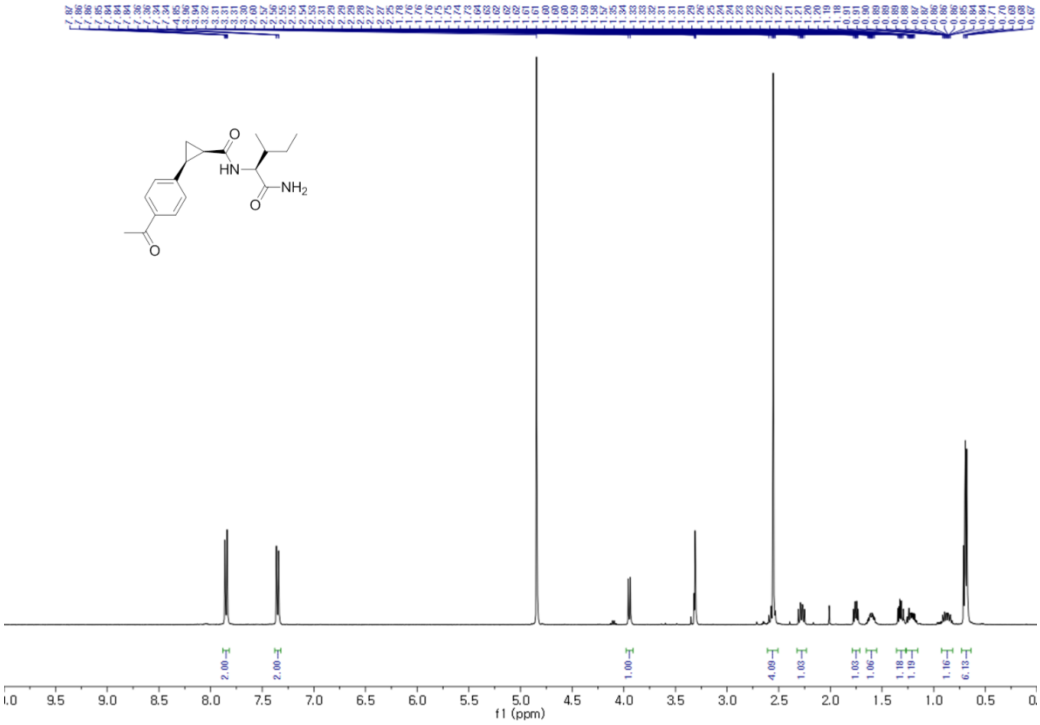

400 MHz, ^{1}H NMR in DMSO-d_6

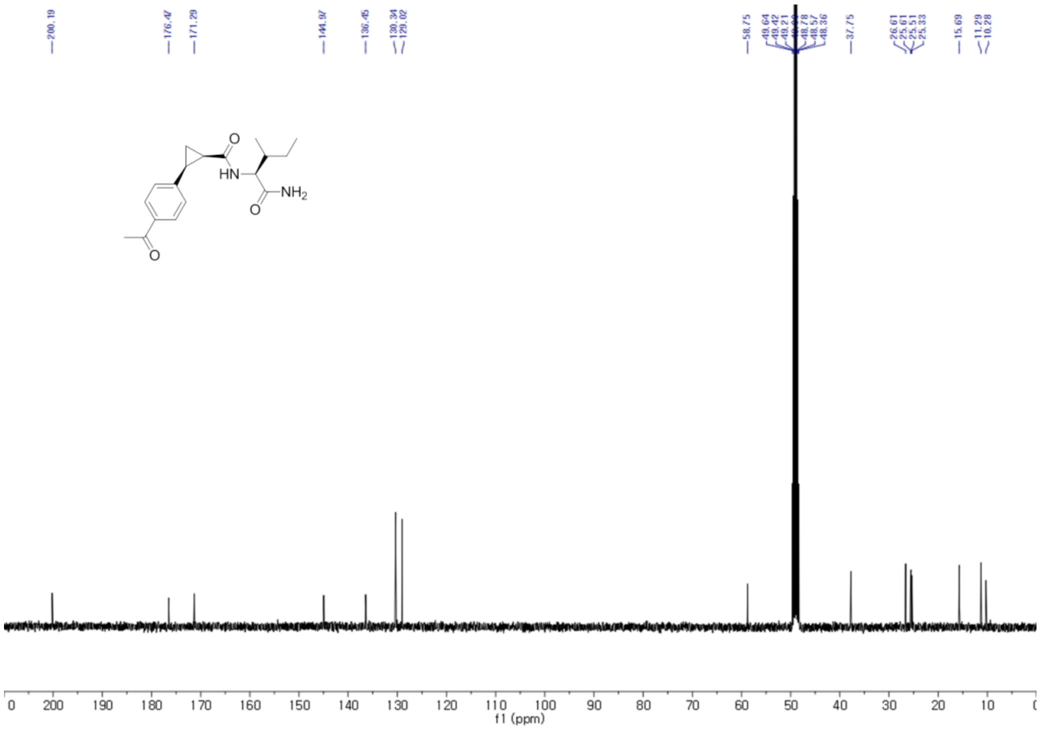

100 MHz, ^{13}C NMR in DMSO-*d*$_6$

(1*R*, 2*S*)-*N*-((2*S*, 3*R*)-1-amino-3-methyl-1-oxopentan-2-yl)-2-(m-tolyl)cyclopropanecarboxamide (3t)

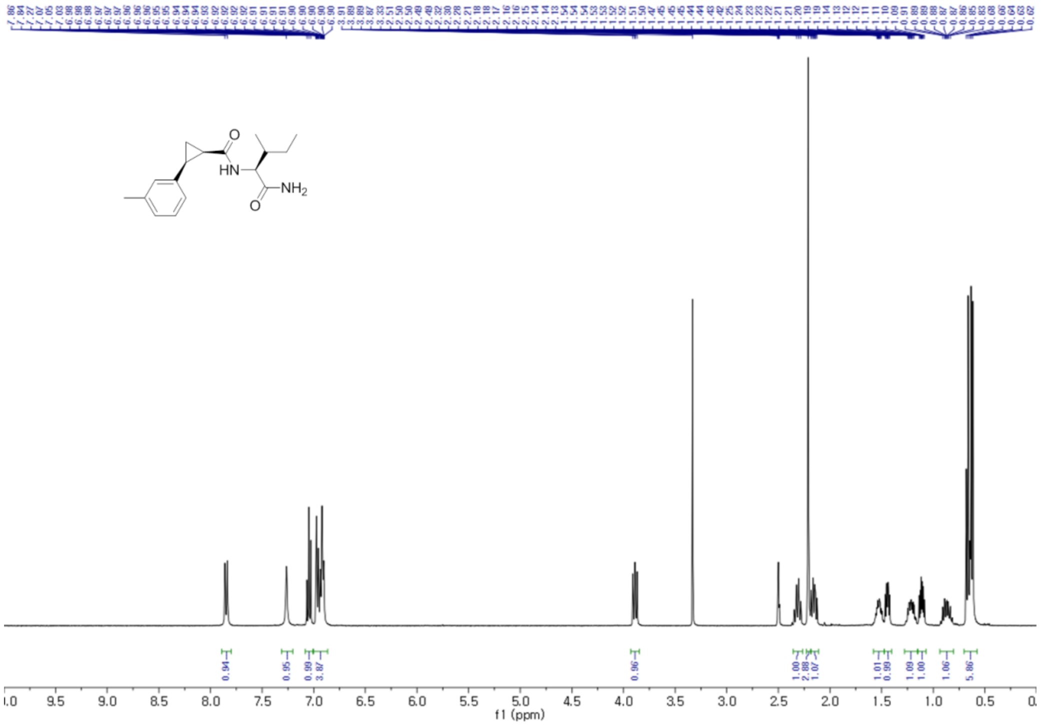

400 MHz, ^{1}H NMR in DMSO-*d*$_6$

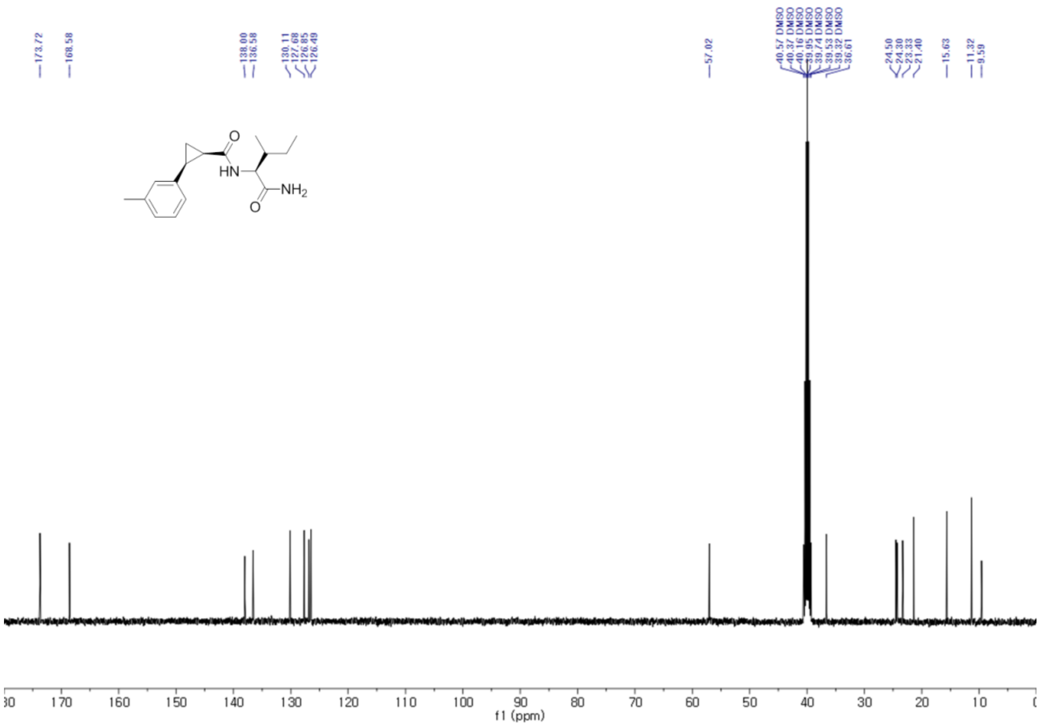

100 MHz, ^{13}C NMR in DMSO-d_6

(1*R*, 2*S*)-*N*-((2*S*, 3*R*)-1-amino-3-methyl-1-oxopentan-2-yl)-2-(3-nitrophenyl)cyclopropanecarboxamide (3u)

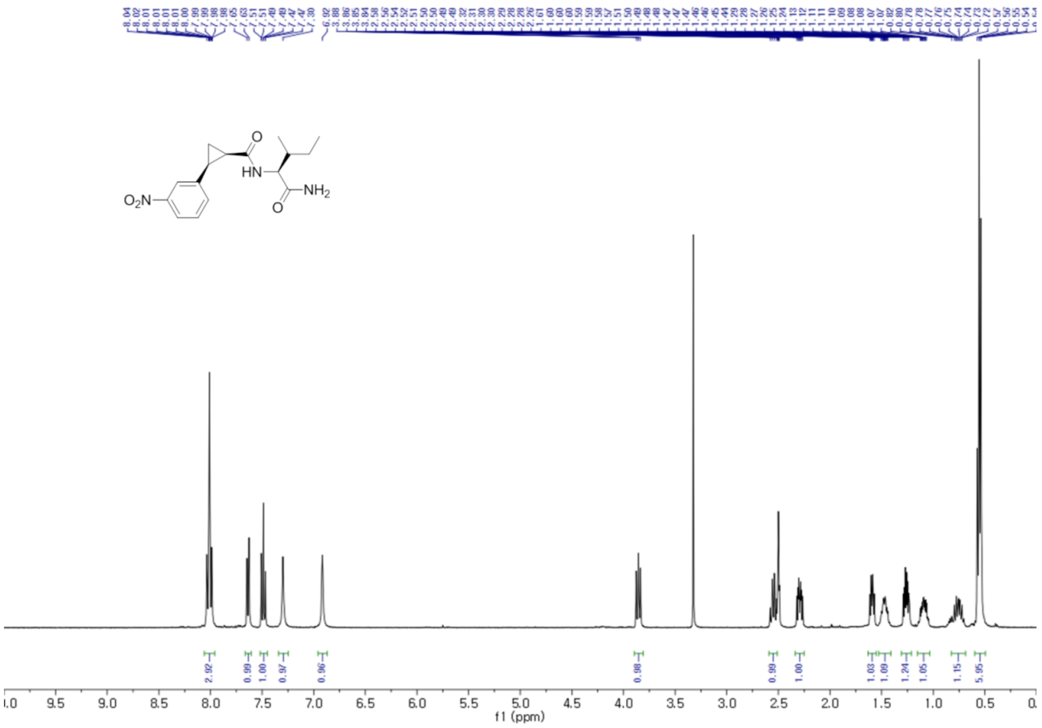

400 MHz, ^{1}H NMR in DMSO-d_6

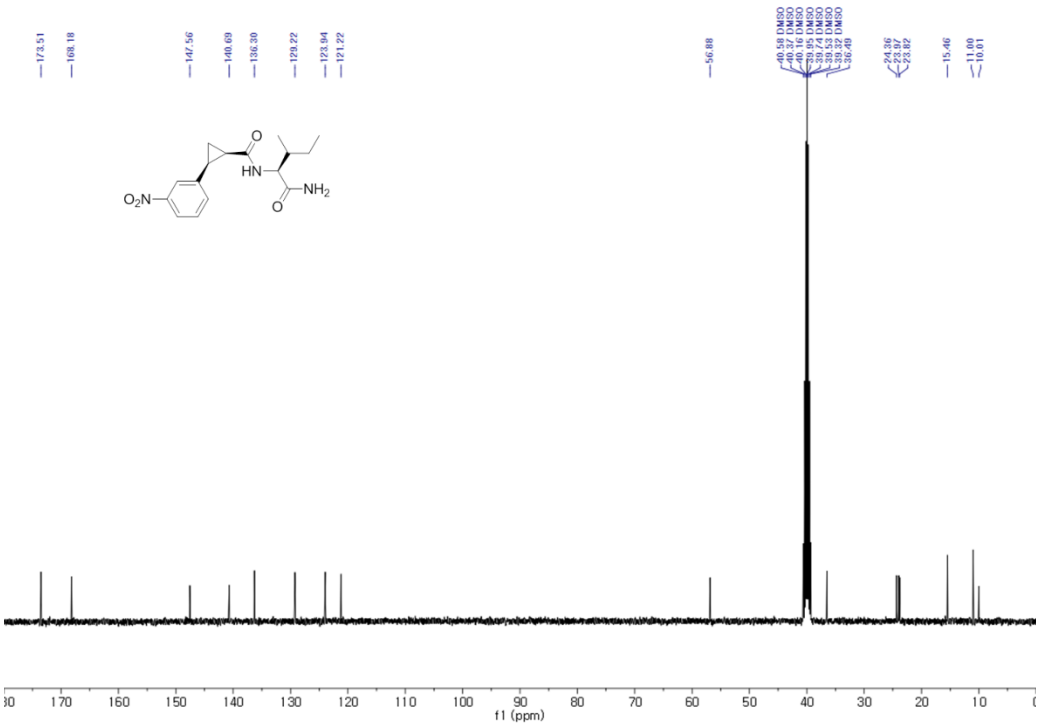

100 MHz, ^{13}C NMR in DMSO-d_6

(1*R*, 2*S*)-*N*-((2*S*, 3*R*)-1-amino-3-methyl-1-oxopentan-2-yl)-2-(4-nitrophenyl)cyclopropanecarboxamide (3v)

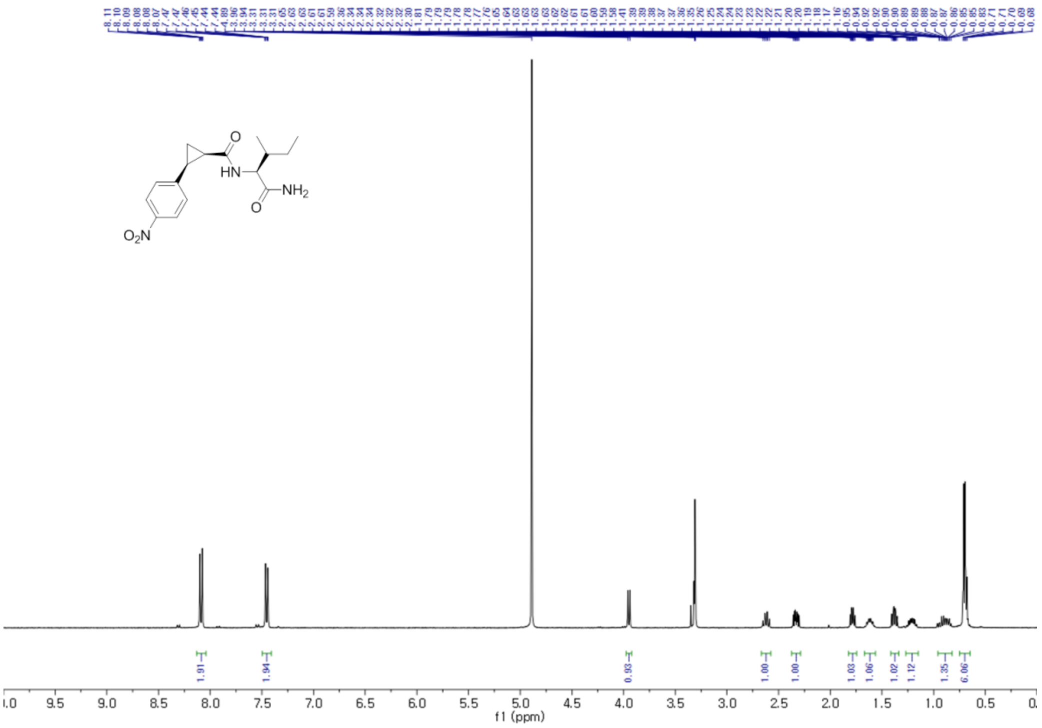

400 MHz, ^{1}H NMR in DMSO-d_6

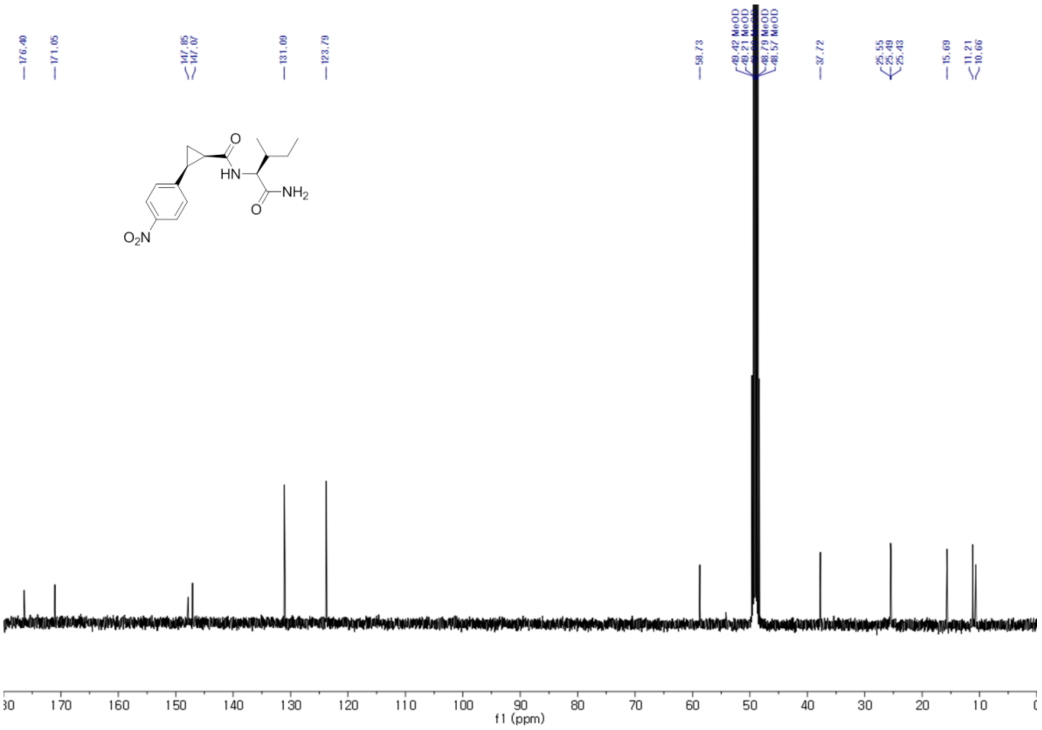

100 MHz, ^{13}C NMR in DMSO-d_6

(1*R*, 2*S*)-*N*-((2*S*, 3*R*)-1-amino-3-methyl-1-oxopentan-2-yl)-2-(2-methoxyphenyl)cyclopropanecarboxamide (3w)

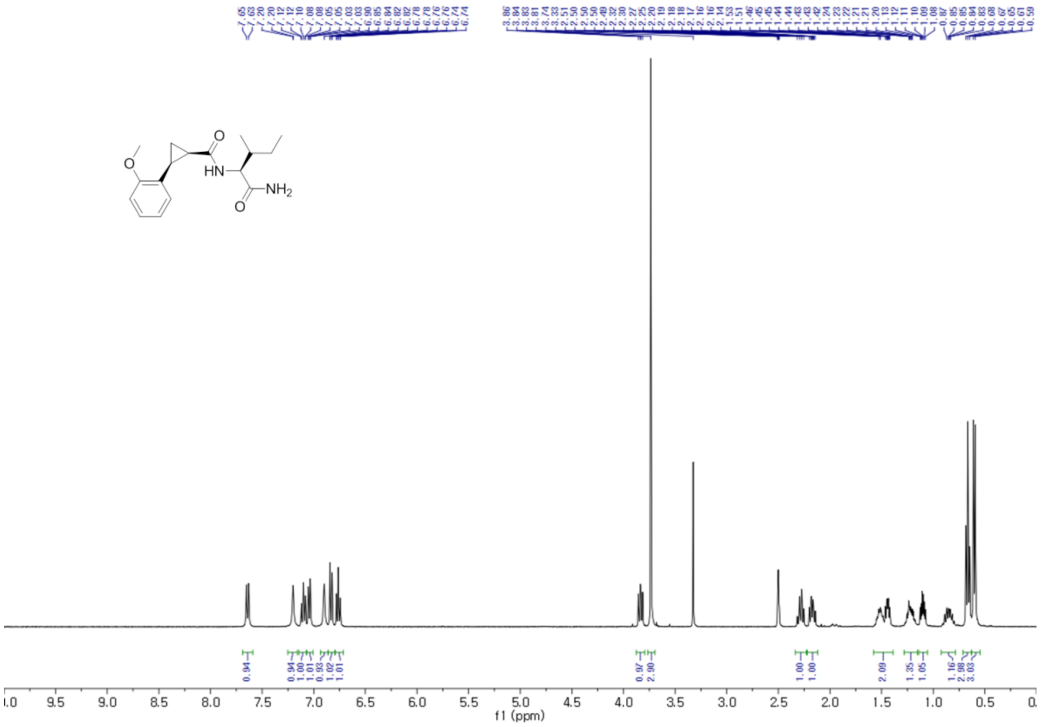

400 MHz, ^{1}H NMR in DMSO-d_6

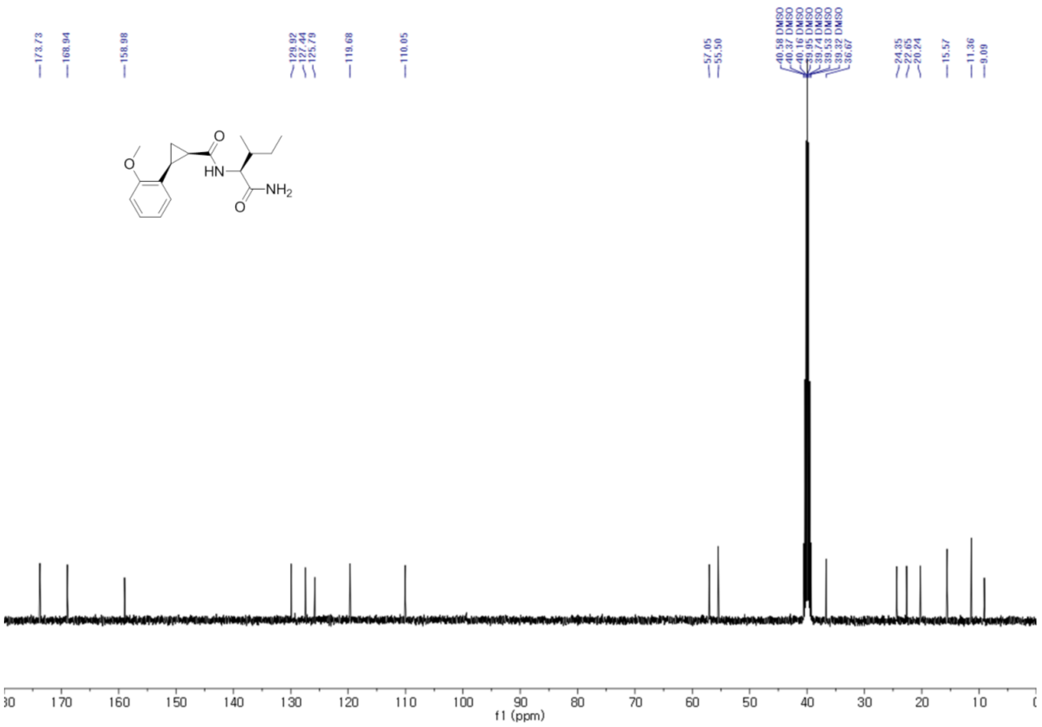

100 MHz, ^{13}C NMR in DMSO-d_6

(1*R*, 2*S*)-*N*-((2*S*, 3*R*)-1-amino-3-methyl-1-oxopentan-2-yl)-2-(2-chlorophenyl)cyclopropanecarboxamide (3x)

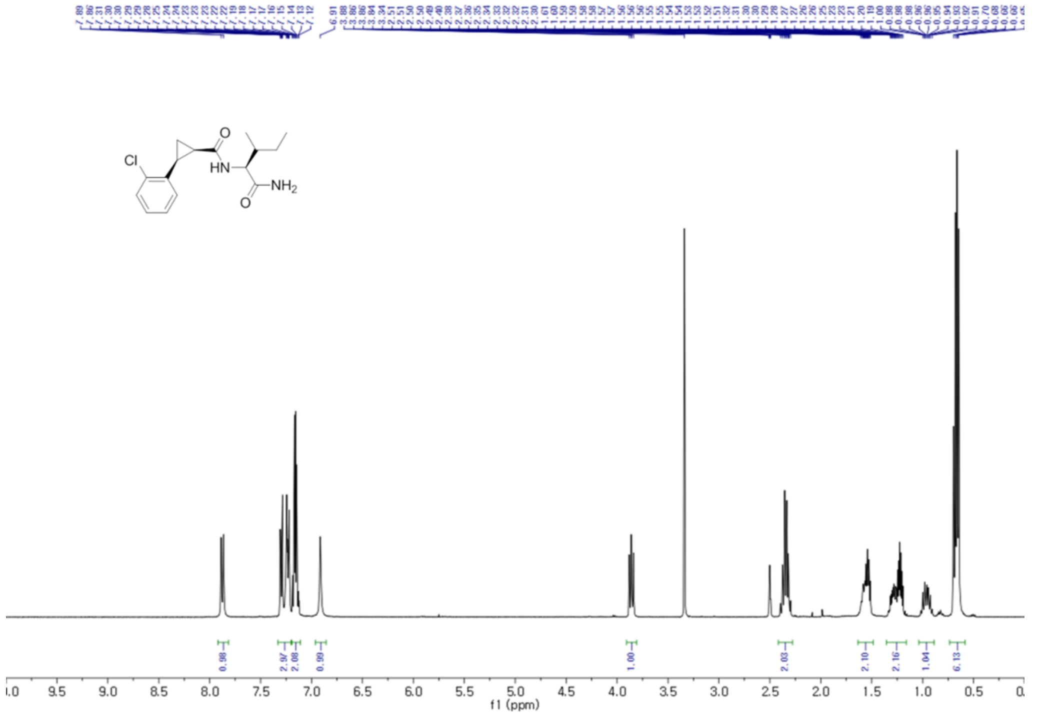

400 MHz, ^{1}H NMR in DMSO-d_6

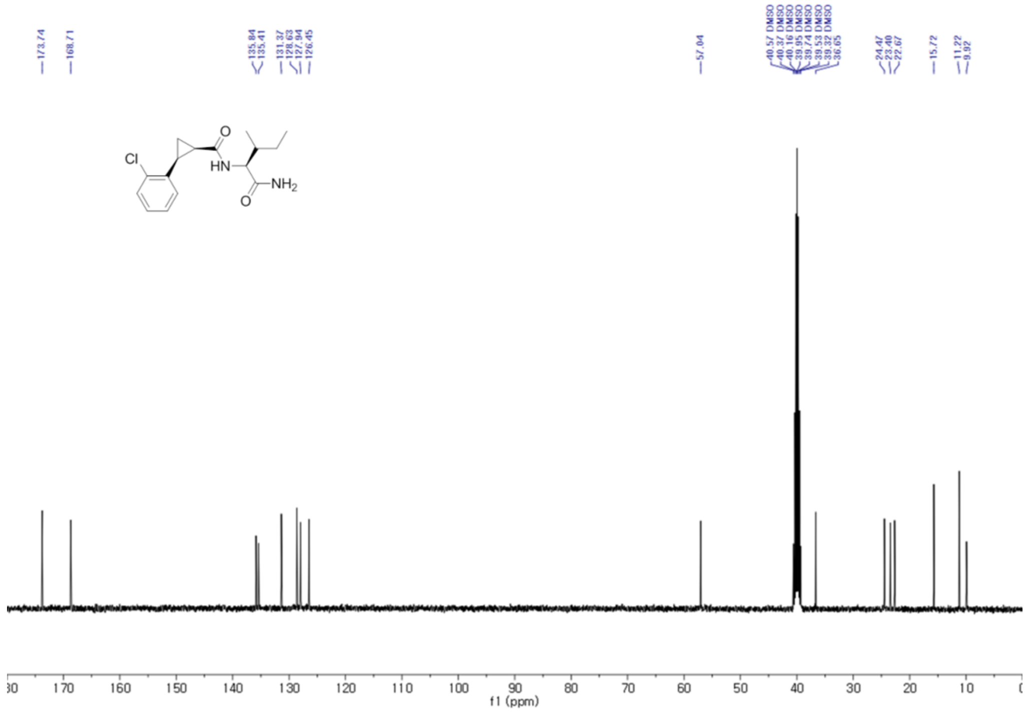

100 MHz, ^{13}C NMR in DMSO-d_6

(1*R*, 2*S*)-*N*-((2*S*, 3*R*)-1-amino-3-methyl-1-oxopentan-2-yl)-2-(2-fluorophenyl)cyclopropanecarboxamide (3y)

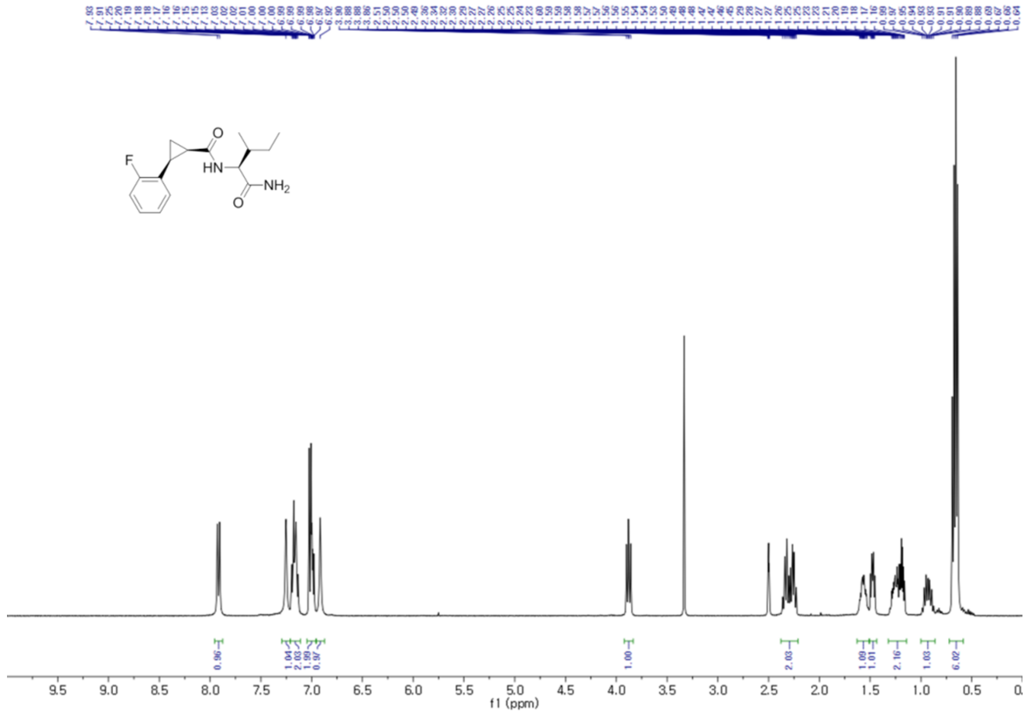

400 MHz, ^{1}H NMR in DMSO-d_6

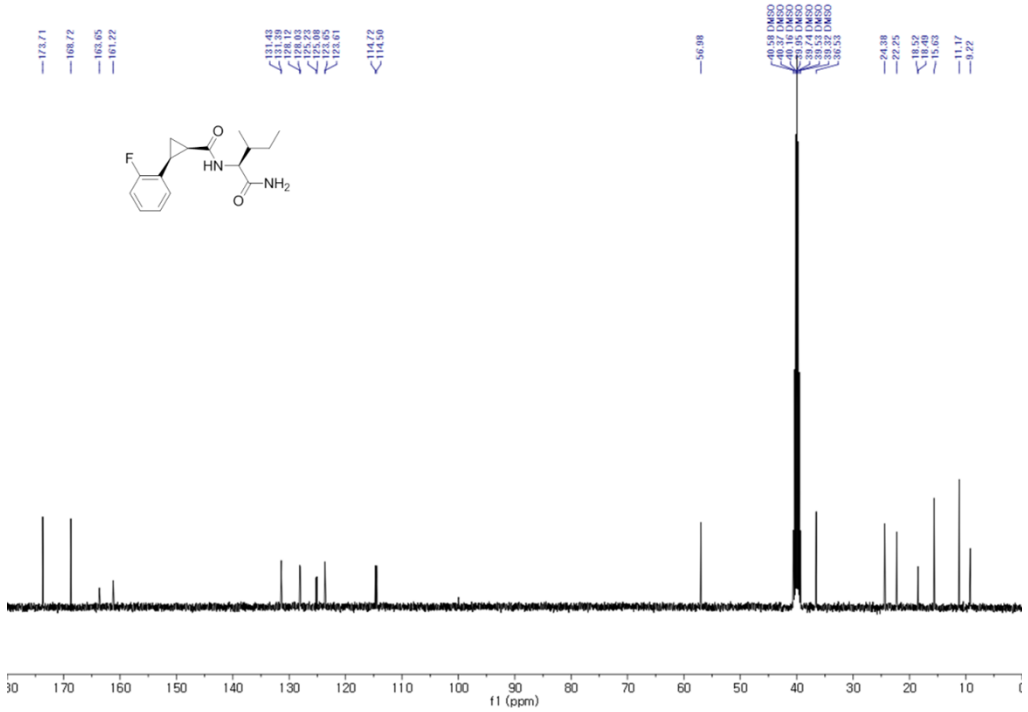

100 MHz, ^{13}C NMR in DMSO-d_6

(1*R*, 2*S*)-*N*-((S)-1-amino-3-methyl-1-oxobutan-2-yl)-2-((*tert*-butyldimethylsilyl)ethynyl)cyclopropane-1-carboxamide (4)

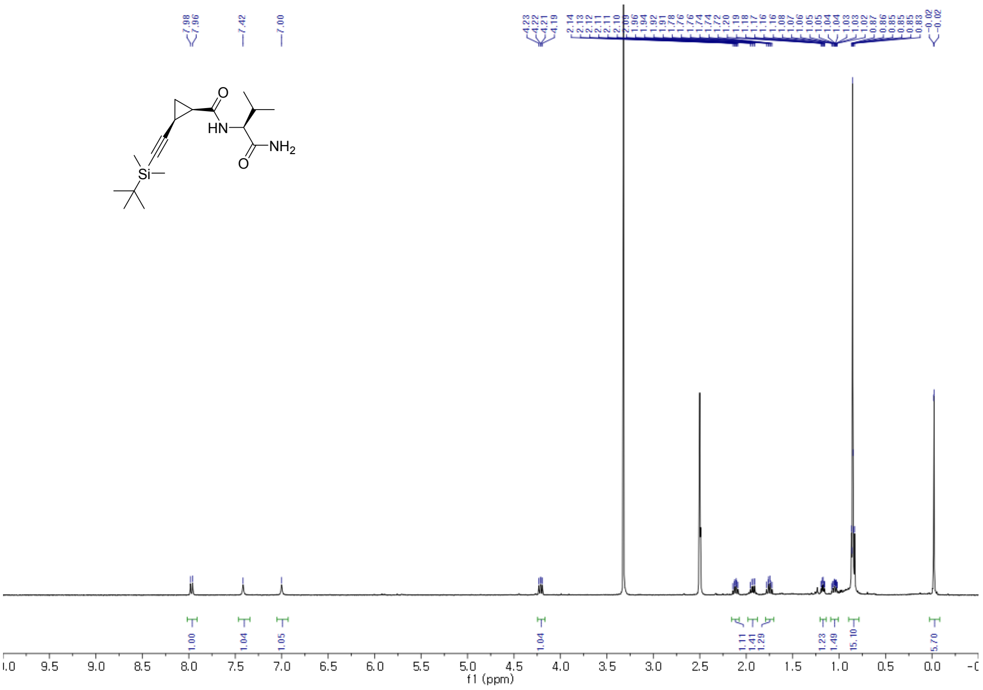

400 MHz, ^{1}H NMR in DMSO-d_6

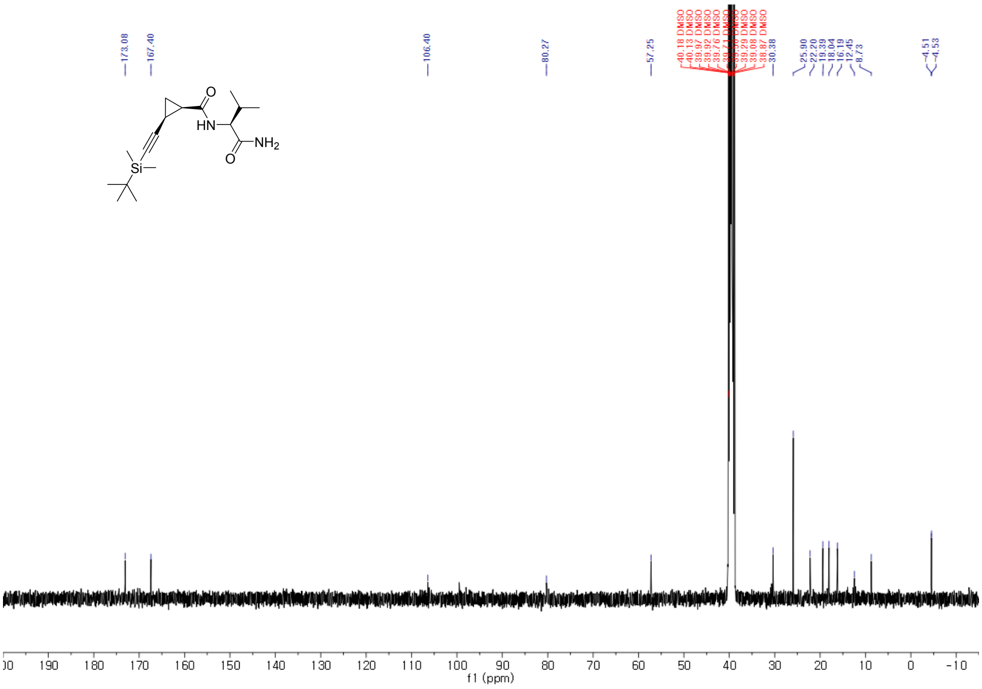

100 MHz, ^{13}C NMR in DMSO-d_6

Crystallographic Data for 2a

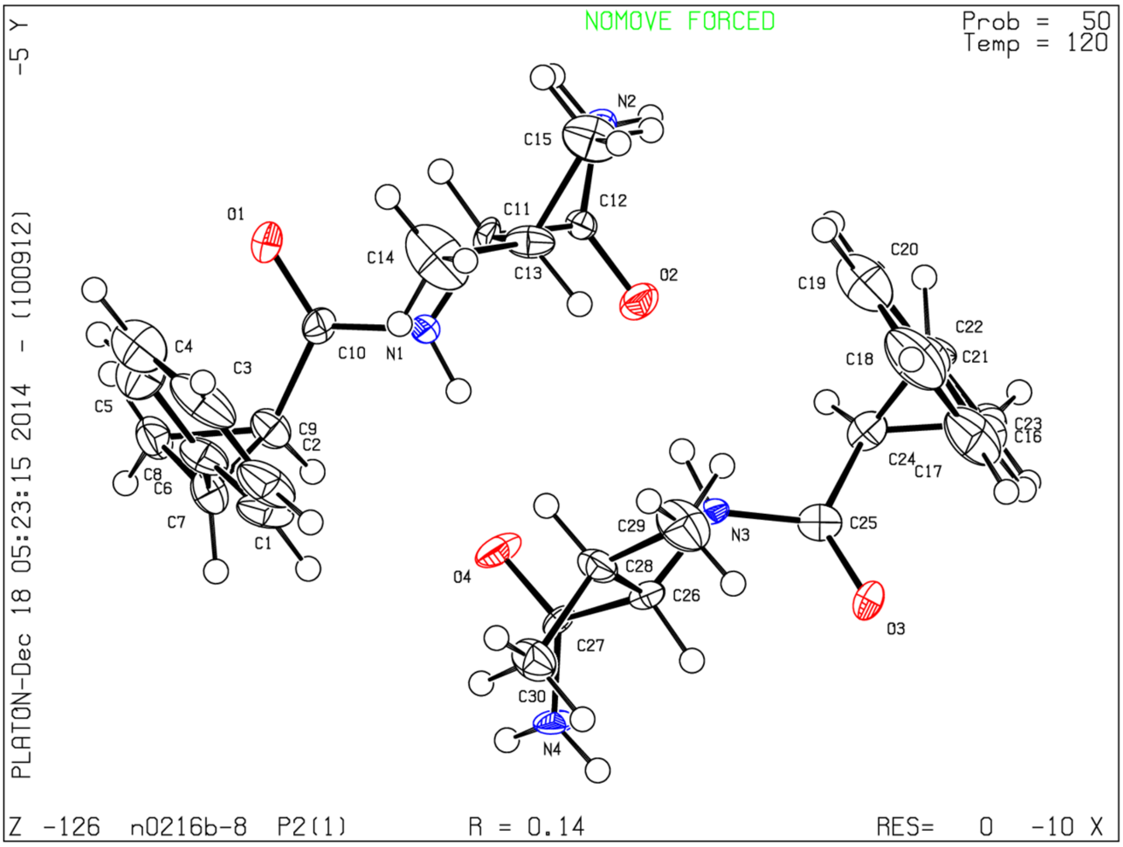

Table S1. Crystal data and structure refinement for **2a**.

Identification code	n0216b-8	
Empirical formula	C15 H20 N2 O2	
Formula weight	260.33	
Temperature	120(2) K	
Wavelength	0.71073 Å	
Crystal system	Monoclinic	
Space group	P2(1)	
Unit cell dimensions	a = 9.4882(18) Å	α= 90°.
	b = 10.3774(19) Å	β= 107.509(6)°.
	c = 15.575(3) Å	γ = 90°.
Volume	1462.5(5) Å^3	
Z	4	
Density (calculated)	1.182 Mg/m^3	
Absorption coefficient	0.079 mm^{-1}	

F(000)	560
Crystal size	0.40 x 0.06 x 0.04 mm^3
Theta range for data collection	2.97 to 28.28°.
Index ranges	-11<=h<=12, -13<=k<=13, -20<=l<=20
Reflections collected	15954
Independent reflections	5733 [R(int) = 0.0651]
Completeness to theta = 25.00°	76.7 %
Absorption correction	Semi-empirical from equivalents
Max. and min. transmission	0.9968 and 0.9691
Refinement method	Full-matrix least-squares on F^2
Data / restraints / parameters	5733 / 337 / 343
Goodness-of-fit on F^2	1.148
Final R indices [I>2sigma(I)]	R1 = 0.1445, wR2 = 0.3846
R indices (all data)	R1 = 0.1687, wR2 = 0.3965
Largest diff. peak and hole	0.491 and -0.559 e.$Å^{-3}$

Table S2. Atomic coordinates (x 10^4) and equivalent isotropic displacement parameters ($Å^2$x 10^3) for **2a**. U(eq) is defined as one third of the trace of the orthogonalized U^{ij} tensor.

	x	y	z	U(eq)
N(1)	8856(7)	7925(6)	6454(4)	17(1)
N(2)	9637(8)	4727(7)	6147(5)	28(2)
O(1)	10970(6)	8865(5)	6388(4)	27(1)
O(2)	7425(6)	5525(6)	6112(4)	26(1)
C(1)	8591(11)	11152(10)	7885(6)	36(2)
C(2)	9195(14)	11173(10)	8797(7)	43(2)
C(3)	10705(15)	11305(10)	9180(6)	48(2)
C(4)	11581(13)	11407(10)	8606(7)	42(2)
C(5)	10946(12)	11357(11)	7675(7)	41(2)
C(6)	9442(10)	11236(8)	7318(5)	27(2)
C(7)	8659(10)	11194(8)	6310(5)	26(2)
C(8)	9415(10)	11183(8)	5605(6)	29(2)
C(9)	8699(10)	10003(8)	5772(5)	25(2)
C(10)	9604(8)	8896(7)	6208(5)	19(1)
C(11)	9551(8)	6757(7)	6882(5)	20(1)
C(12)	8774(8)	5607(7)	6340(5)	16(1)
C(13)	9515(9)	6611(8)	7872(5)	26(2)
C(14)	10240(17)	7772(11)	8428(7)	54(3)
C(15)	10319(12)	5370(10)	8285(6)	37(2)
N(3)	4762(7)	6404(6)	6615(4)	19(1)
N(4)	3331(7)	9247(7)	5646(4)	22(1)
O(3)	2655(6)	5516(6)	6712(4)	25(1)
O(4)	5687(6)	8627(7)	5846(4)	30(1)
C(16)	4140(11)	3602(10)	8300(6)	35(2)
C(17)	4541(15)	3926(10)	9225(7)	47(2)
C(18)	6004(16)	4072(11)	9755(7)	52(2)
C(19)	7066(14)	3934(10)	9353(7)	46(2)
C(20)	6760(10)	3632(8)	8422(7)	35(2)
C(21)	5262(10)	3456(7)	7903(5)	26(2)
C(22)	4979(10)	3205(9)	6931(6)	32(2)

C(23)	3539(9)	2983(8)	6282(6)	28(2)
C(24)	4336(9)	4204(8)	6221(5)	23(1)
C(25)	3810(9)	5404(8)	6537(5)	22(1)
C(26)	4432(8)	7712(7)	6848(5)	18(1)
C(27)	4551(7)	8583(7)	6081(4)	15(1)
C(28)	5437(9)	8209(7)	7744(5)	23(1)
C(29)	5499(13)	7257(10)	8504(7)	41(2)
C(30)	4960(11)	9558(8)	7930(6)	30(2)

Table S3. Bond lengths [Å] and angles [°] for **2a**.

N(1)-C(10)	1.353(9)
N(1)-C(11)	1.444(9)
N(2)-C(12)	1.320(10)
O(1)-C(10)	1.241(9)
O(2)-C(12)	1.225(9)
C(1)-C(2)	1.362(14)
C(1)-C(6)	1.367(13)
C(2)-C(3)	1.383(18)
C(3)-C(4)	1.396(17)
C(4)-C(5)	1.393(14)
C(5)-C(6)	1.372(14)
C(6)-C(7)	1.523(11)
C(7)-C(8)	1.482(11)
C(7)-C(9)	1.500(11)
C(8)-C(9)	1.461(11)
C(9)-C(10)	1.472(11)
C(11)-C(12)	1.518(10)
C(11)-C(13)	1.559(10)
C(13)-C(14)	1.522(13)
C(13)-C(15)	1.536(12)
N(3)-C(25)	1.357(11)
N(3)-C(26)	1.463(9)
N(4)-C(27)	1.342(9)

O(3)-C(25)	1.212(9)
O(4)-C(27)	1.239(8)
C(16)-C(21)	1.392(12)
C(16)-C(17)	1.415(14)
C(17)-C(18)	1.394(19)
C(18)-C(19)	1.345(18)
C(19)-C(20)	1.426(14)
C(20)-C(21)	1.419(12)
C(21)-C(22)	1.478(11)
C(22)-C(23)	1.453(13)
C(22)-C(24)	1.505(12)
C(23)-C(24)	1.494(11)
C(24)-C(25)	1.479(10)
C(26)-C(28)	1.523(10)
C(26)-C(27)	1.529(10)
C(28)-C(30)	1.526(10)
C(28)-C(29)	1.530(12)

C(10)-N(1)-C(11)	123.4(6)
C(2)-C(1)-C(6)	121.8(10)
C(1)-C(2)-C(3)	120.5(9)
C(2)-C(3)-C(4)	118.1(9)
C(5)-C(4)-C(3)	120.6(10)
C(6)-C(5)-C(4)	119.8(9)
C(1)-C(6)-C(5)	119.3(8)
C(1)-C(6)-C(7)	117.6(8)
C(5)-C(6)-C(7)	123.1(8)
C(8)-C(7)-C(9)	58.7(5)
C(8)-C(7)-C(6)	124.7(8)
C(9)-C(7)-C(6)	120.9(7)
C(9)-C(8)-C(7)	61.3(5)
C(8)-C(9)-C(10)	119.9(8)
C(8)-C(9)-C(7)	60.0(5)
C(10)-C(9)-C(7)	119.7(7)
O(1)-C(10)-N(1)	121.2(7)

O(1)-C(10)-C(9)	123.3(7)
N(1)-C(10)-C(9)	115.4(7)
N(1)-C(11)-C(12)	109.0(6)
N(1)-C(11)-C(13)	113.3(6)
C(12)-C(11)-C(13)	108.8(6)
O(2)-C(12)-N(2)	123.5(7)
O(2)-C(12)-C(11)	120.4(6)
N(2)-C(12)-C(11)	116.1(6)
C(14)-C(13)-C(15)	110.0(8)
C(14)-C(13)-C(11)	110.2(8)
C(15)-C(13)-C(11)	109.9(6)
C(25)-N(3)-C(26)	123.0(6)
C(21)-C(16)-C(17)	118.1(9)
C(18)-C(17)-C(16)	122.9(10)
C(19)-C(18)-C(17)	117.7(9)
C(18)-C(19)-C(20)	123.1(10)
C(21)-C(20)-C(19)	118.0(10)
C(16)-C(21)-C(20)	120.1(8)
C(16)-C(21)-C(22)	123.1(8)
C(20)-C(21)-C(22)	116.6(8)
C(23)-C(22)-C(21)	125.7(8)
C(23)-C(22)-C(24)	60.6(5)
C(21)-C(22)-C(24)	122.6(7)
C(22)-C(23)-C(24)	61.4(6)
C(25)-C(24)-C(23)	117.7(6)
C(25)-C(24)-C(22)	116.0(6)
C(23)-C(24)-C(22)	58.0(6)
O(3)-C(25)-N(3)	121.9(7)
O(3)-C(25)-C(24)	125.0(8)
N(3)-C(25)-C(24)	113.1(6)
N(3)-C(26)-C(28)	114.8(6)
N(3)-C(26)-C(27)	106.4(5)
C(28)-C(26)-C(27)	110.0(6)
O(4)-C(27)-N(4)	121.8(6)
O(4)-C(27)-C(26)	121.9(6)

N(4)-C(27)-C(26)	116.3(6)
C(26)-C(28)-C(30)	110.2(7)
C(26)-C(28)-C(29)	111.0(7)
C(30)-C(28)-C(29)	112.9(7)

Symmetry transformations used to generate equivalent atoms:

Table S4. Anisotropic displacement parameters ($Å^2$x 10^3) for **2a**. The anisotropic displacement factor exponent takes the form: $-2\pi^2[h^2 a^{*2}U^{11} + ... + 2 h k a^* b^* U^{12}]$

	U^{11}	U^{22}	U^{33}	U^{23}	U^{13}	U^{12}
N(1)	14(3)	12(2)	26(3)	-2(2)	6(2)	0(2)
N(2)	18(3)	30(4)	34(4)	-8(3)	7(3)	-4(3)
O(1)	22(3)	24(3)	37(3)	5(2)	13(2)	-5(2)
O(2)	19(3)	25(3)	38(3)	-11(2)	13(2)	-3(2)
C(1)	40(4)	35(5)	37(4)	1(4)	17(4)	19(4)
C(2)	70(6)	30(5)	39(4)	6(4)	32(5)	20(5)
C(3)	83(6)	31(4)	21(4)	4(3)	4(4)	21(5)
C(4)	48(5)	38(5)	29(4)	-5(4)	-3(3)	2(4)
C(5)	39(4)	46(5)	35(4)	-2(4)	9(4)	-4(4)
C(6)	35(4)	23(4)	19(3)	1(3)	1(3)	10(3)
C(7)	35(4)	18(3)	25(3)	-3(3)	9(3)	-2(3)
C(8)	36(4)	20(3)	34(4)	6(3)	16(4)	1(3)
C(9)	31(4)	22(3)	20(3)	4(3)	7(3)	3(3)
C(10)	21(3)	22(3)	19(3)	0(2)	13(3)	-1(3)
C(11)	13(3)	21(3)	25(3)	-1(2)	3(3)	-8(3)
C(12)	14(3)	13(3)	18(3)	-2(2)	0(3)	0(2)
C(13)	25(4)	30(4)	21(3)	-5(3)	5(3)	10(3)
C(14)	78(8)	40(5)	23(4)	-6(4)	-16(5)	1(5)
C(15)	45(5)	42(5)	19(3)	6(3)	4(4)	11(4)
N(3)	13(3)	18(3)	28(3)	6(2)	11(2)	4(2)
N(4)	14(3)	29(3)	24(3)	15(2)	7(2)	11(2)
O(3)	20(3)	25(3)	35(3)	4(2)	15(2)	-4(2)

O(4)	14(2)	48(4)	29(3)	19(3)	10(2)	7(2)
C(16)	34(4)	43(5)	32(4)	18(4)	15(3)	7(4)
C(17)	79(6)	36(5)	33(4)	17(4)	25(4)	12(5)
C(18)	87(7)	37(5)	29(4)	12(4)	12(4)	11(5)
C(19)	61(6)	32(5)	30(4)	-3(4)	-7(4)	2(5)
C(20)	28(4)	22(4)	44(4)	1(3)	-5(3)	-1(3)
C(21)	35(4)	16(3)	26(3)	0(3)	10(3)	3(3)
C(22)	32(4)	29(4)	30(3)	-10(3)	4(3)	14(3)
C(23)	29(4)	26(3)	32(4)	0(3)	16(3)	1(3)
C(24)	21(3)	28(3)	20(3)	-8(3)	7(3)	-3(3)
C(25)	24(4)	28(3)	13(3)	-2(3)	4(3)	4(3)
C(26)	12(3)	22(3)	21(3)	-1(2)	5(2)	2(2)
C(27)	11(3)	22(3)	16(3)	-1(2)	9(2)	-1(2)
C(28)	24(4)	16(3)	27(3)	-3(3)	2(3)	4(3)
C(29)	50(6)	38(5)	27(4)	11(4)	-1(4)	6(4)
C(30)	36(5)	22(3)	33(4)	-5(3)	9(4)	2(3)

Table S5. Hydrogen coordinates (x 10^4) and isotropic displacement parameters (Å^{2}x 10 ^{3}) for **2a**.

	x	y	z	U(eq)
H(1B)	7895	8009	6347	21
H(2B)	9247	4036	5839	33
H(2C)	10602	4831	6327	33
H(1A)	7549	11077	7636	43
H(2A)	8575	11096	9172	52
H(3A)	11133	11326	9815	57
H(4A)	12620	11511	8852	50
H(5A)	11552	11407	7289	49
H(7A)	7683	11647	6126	31
H(8A)	8941	11656	5039	34
H(8B)	10508	11205	5803	34

H(9A)	7750	9781	5305	30
H(11A)	10605	6756	6883	24
H(13A)	8462	6558	7871	31
H(14A)	10209	7670	9048	81
H(14B)	9707	8557	8168	81
H(14C)	11270	7836	8428	81
H(15A)	10282	5279	8905	55
H(15B)	11351	5412	8287	55
H(15C)	9836	4627	7929	55
H(3B)	5617	6259	6521	22
H(4B)	3324	9730	5180	27
H(4C)	2538	9199	5826	27
H(16A)	3133	3487	7960	42
H(17A)	3778	4048	9496	57
H(18A)	6243	4263	10379	63
H(19A)	8067	4042	9706	55
H(20A)	7536	3550	8156	42
H(22A)	5780	2687	6801	38
H(23A)	2662	3041	6500	33
H(23B)	3469	2322	5813	33
H(24A)	4779	4281	5717	27
H(26A)	3388	7734	6868	22
H(28A)	6457	8278	7688	28
H(29A)	5798	6407	8347	62
H(29B)	4521	7194	8592	62
H(29C)	6217	7559	9062	62
H(30A)	4922	10122	7419	46
H(30B)	5672	9903	8475	46
H(30C)	3979	9516	8016	46

Table S6. Hydrogen bonds for N0216B-8 [Å and °].

D-H...A	d(D-H)	d(H...A)	d(D...A)	<(DHA)

N(1)-H(1B)...O(4)	0.88	2.10	2.959(8)	163.6
N(3)-H(3B)...O(2)	0.88	2.14	3.003(8)	166.3
N(2)-H(2C)...O(3)#1	0.88	1.99	2.851(9)	166.1
N(4)-H(4B)...O(2)#2	0.88	2.09	2.931(8)	159.0

Symmetry transformations used to generate equivalent atoms:

#1 x+1,y,z #2 -x+1,y+1/2,-z+1
